# Training Schedule Affects Operant Responding Independent of Motivation in the Neuroligin‐3 R451C Mouse Model of Autism

**DOI:** 10.1111/gbb.70032

**Published:** 2025-08-15

**Authors:** Riki Dingwall, Carlos May, Jackson A. McDonald, Thomas Hill, Robyn Brown, Andrew J. Lawrence, Anthony J. Hannan, Emma L. Burrows

**Affiliations:** ^1^ The Florey Institute of Neuroscience and Mental Health, University of Melbourne Parkville, Melbourne Australia; ^2^ Faculty of Medicine, Dentistry & Health Sciences University of Melbourne Parkville Victoria Australia; ^3^ Department of Biochemistry & Pharmacology University of Melbourne Parkville Victoria Australia

**Keywords:** atomoxetine, autism, cocaine, methylphenidate, motivation, neuroligin‐3, R451C, study design, touchscreen

## Abstract

Autism affects ~1 in 100 people and arises from the interplay between rare genetic changes and the environment. Diagnosis is based on social and communication difficulties, as well as the presence of restricted and repetitive behaviours. Autism aetiology is complex. However, the social motivation hypothesis proposes that an imbalance in the salience of social over non‐social stimuli contributes over time to the autism phenotype. Accordingly, motivational dysfunction in autism is widespread, and human imaging data has identified broad impairments to reward processing. The R451C mutation of the neuroligin‐3 gene is one such rare genetic change. Knock‐in mice harbouring this mutation (NL3) exhibit a range of autism‐related phenotypes, including impaired sociability and social motivation. However, no prior report has directly probed non‐social motivation. Here, we explore conflicting results from the progressive ratio (PR) and conditioned place preference tasks of non‐social motivation. Initial PR results were inconsistent, suggesting reduced, unaltered, and elevated non‐social motivation, respectively. Utilising several experimental designs, we probed a range of confounders likely to influence task performance. Overall, reduced PR responding by NL3s likely arose from a combination of their superior ability to withhold responding during prior training and a short PR training schedule. Meanwhile, increased PR responding by NL3s was attributable to their heightened degree of habitual responding. The NL3 mouse model therefore likely best represents autistic individuals with intact non‐social motivation but altered behavioural updating. Finally, we discuss the benefits and limitations of using heterogenous experimental designs to probe behavioural phenotypes and offer some general recommendations for PR.

## Graphical abstract

Motivation may contribute to the autism phenotype. We however found no overall evidence of motivational dysfunction in a mouse model of autism. Instead, differences were more readily explained by training schedules that encouraged the formation of habitual behaviours. Our findings offer considerations for experimental designs to parse motivation.

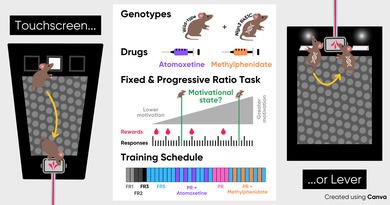

**Abbreviations**
ATO: atomoxetine
CPP: conditioned place preference
DA: dopamine
FFW: free‐feeding weight
FR: fixed ratio
GLM: general linear model
GLMM: general linear mixed model
i.p.: intraperitoneal
MPH: methylphenidate
NE: norepinephrine
NL3: neuroligin‐3 R451C mouse model
NLGN3: neuroligin‐3 gene
PR: progressive ratio
R451C: arginine‐to‐cysteine substitution at position 451
rCPT: rodent‐adapted continuous performance test
WT: wild type

## 1 Introduction

Autism is a diagnostic construct defined by the presence of social and communication difficulties, as well as restricted and repetitive behaviours [1]. Conservative estimates suggest an average prevalence of ~1 in every 100 people [2]. Though impact varies greatly between individuals, most autistic people require additional support during at least one life stage [3]. The aetiology of autism is accordingly complex. However, high heritability in family and twin studies suggests a predominant role of genetics. Over 1000 genes have contemporarily been associated to varying degrees of confidence with relative autism risk [4]. Several environmental contributors have also been proposed, but none have to date been conclusively linked to autism [5]. The mechanism by which these putative genetic and environmental factors contribute combinatorially to the overarching autism phenotype remains unclear [6, 7]. However, the social motivation hypothesis proposes that an imbalance in engagement with non‐social over social stimuli manifests over time as the core autism characteristics [8]. Reports of motivational dysfunction in autism are common but not ubiquitous, in keeping with the established heterogeneity of autism‐related phenotypes. Some report that motivational impairments are most pronounced when the reward is social [9], though altered neural processing of both social and non‐social rewards is widespread in the autism population [8]. In the present study, we examine the neuroligin‐3 (NLGN3) R451C mouse model (NL3) of autism during the performance of non‐social tasks of reward motivation.

NLGN3 is one such high‐confidence candidate gene for autism [10]. The neuroligins are a family of transmembrane cell adhesion proteins involved in synapse formation and maintenance [11]. Neuroligins 1–3 are highly conserved in mammals with 96%–98% amino acid homology from humans to mice. During neurodevelopment, the neuroligins induce synapse differentiation, cluster the postsynaptic machinery, and hold the presynapse in close apposition through their association with neurexins and other putative presynaptic binding partners [12]. Arginine‐to‐cysteine substitution at amino acid position 451 (R451C) of NLGN3 was first identified in two brothers with non‐syndromic autism, inherited from their non‐autistic heterozygotic mother [10]. This mutation occurs in the extracellular domain near the site of neuroligin dimerisation, but does not appear to directly interfere with it [13]. Instead, only ~10% of the translated protein successfully reaches the plasma membrane, with the remaining retained in the endoplasmic reticulum and eventually degraded by the proteasome [14, 15]. At the synapse, R451C‐*Nlgn3* exhibits reduced binding to its presynaptic binding partners, β‐neurexins and PTP‐δ, and enhances excitatory spine turnover [14, 16, 17]. Behavioural assessment of the NLGN3 R451C knock‐in mouse (NL3) has revealed a mild phenotype, with decreased sociability, increased aggression, improved spatial learning, altered reversal learning, and conservative task responding [18, 19, 20, 21, 22, 23, 24, 25, 26, 27].

No report to date has directly probed non‐social motivation in the NL3 mouse model. This is despite indications of an altered motivational state, including social interaction deficits and increased task response times [18, 19, 21, 22, 25, 27]. We therefore hypothesised that NL3 mice would exhibit impaired performance in the progressive ratio (PR) and conditioned place preference (CPP) tasks of non‐social reward motivation. Further, NL3 mice exhibit evidence of altered impulsivity, including reduced anticipatory responses and an improved ability to withhold responding [20, 24]. As impulsivity accounts for a portion of total responses in operant tasks [28, 29], we also explored the interaction between measures of motivation and pharmacological interventions for impulsivity in NL3 mice. Methylphenidate (MPH) and atomoxetine (ATO) are two widely prescribed drugs for the treatment of ADHD, though they are increasingly prescribed to those with autism to manage attentional challenges [30]. MPH targets the monoamine system and prevents the reuptake of dopamine (DA) and norepinephrine (NE) from the synaptic cleft [31]. Conversely, ATO selectively inhibits the reuptake of NE [32]. In humans, MPH and ATO reduce impulsive behaviours, permitting the individual greater executive control over their actions [33, 34, 35]. However, in mice, MPH promotes impulsive action, while ATO reduces it [36]. We therefore hypothesised that MPH would improve the performance of NL3 mice in PR by increasing overall interaction with the task. Conversely, we hypothesised that ATO would improve the performance of NL3 mice by facilitating greater attentional control and therefore targeted engagement with the PR task.

Six independent cohorts of NL3 mice underwent PR, though in varied experimental contexts and utilising various protocols. A touchscreen‐based PR protocol was initially utilised to assess motivation in the same environment as our extensive cognitive testing in the NL3 model [37]. A more widely‐used standard lever protocol was then performed to validate our touchscreen findings [38]. Lastly, CPP was performed as an alternative non‐operant measure of conditioned reinforcement to remove the potential confound of habitual instrumental responding [39]. We report them together in the present paper to explore how these different iterations of PR converge to develop our understanding of the NL3 mouse model. We also discuss how the experimental designs and results differ, considering how the training schedule used likely shaped the outcome of our behavioural experiments. Ultimately, we discuss the benefits and limitations of using heterogenous experimental designs to probe behavioural phenotypes and offer some general recommendations for the design of PR experiments. These suggestions are particularly relevant for mouse models of autism, which often exhibit secondary behaviours that affect task performance [40].

## 2 Materials and Methods

### 2.1 Animals

B6;129‐Nlgn3tm1Sud/J mice were acquired (Jackson Laboratories, Bar Harbor, Maine USA; #Jax 008475) and backcrossed for > 10 generations onto the C57BL/6J mouse strain. Heterozygotic females (X/X‐*Nlgn3* ^R451C^) were bred with WT males to generate WT (Y/X) and NL3 (Y/X‐*Nlgn3* ^R451C^) males. The NLGN3 gene is present on the X chromosome, and the mother of the two autistic brothers within whom the R451C mutation was first described was a heterozygotic R451C carrier but not autistic [27]. Experiments were therefore performed solely in male mice. Genotyping was performed by Transnetyx (Cordova, Tennessee, USA) using tail biopsies at 2 weeks. At 4 weeks, mice were weaned and housed in mixed‐genotype groups with 4 males per individually ventilated cage. Food and water were provided ad libitum. At 7–8 weeks, the male mice were single housed in open top cages (at 22°C ± 1°C) due to a known aggression phenotype linked to the knock‐in mutation [19]. Mice were adjusted to a reversed day–night cycle (7 AM–7 PM dark) and maintained on this light cycle throughout testing. At 8 weeks, total body weight was averaged over 3–5 days to calculate an individual free‐feeding weight (FFW). Thereafter, mice were food restricted and maintained at 85% FFW with ad libitum access to water for at least 1 week prior to testing [20]. All experiments were approved under applications #17‐055, #19‐022 and #22‐087 by The Florey Institute of Neuroscience and Mental Health Animal Ethics Committees. In total, 81 WT and 85 NL3 mice were used.

### 2.2 Touchscreen Chambers

The automated touchscreen apparatus (Campden Instruments Ltd. & Lafayette Instrument Co., 2016; [41]) consists of a black trapezoid‐shaped acrylic chamber inside a sound‐attenuated box. At one end, an infrared touchscreen displayed stimuli and recorded responses. A three‐hole mask was placed in front of the touchscreen to guide responses and reduce the interference of unintentional touches. At the other end, a metal tray delivered reward inside a recessed magazine. The rewards used were 5% w/v sucrose or strawberry milk (Nippy's Iced Strawberry Milk; Knispel Brothers Pty Ltd), experimental details for which are contained in Table 1. Three infrared beams tracked movement using timestamped beam breaks at distinct regions of the chamber: (i) the front beam in front of the touchscreen, (ii) the back beam prior to the reward tray, and (iii) the tray beam inside the reward magazine. A bright house light was positioned above the stage, and there was a dim tray light in the reward magazine.

**TABLE 1:** Logistical details of the fixed and progressive ratio experiments.

Details	FR/PR 1	FR/PR 2	FR/PR 3	FR/PR 4	FR/PR 5	FR/PR 6
Experimenter	TH	CM	JM	JM	RD	CM
System	Touchscreen	Touchscreen	Operant	Operant	Operant	Touchscreen
Food restricted	Yes	Yes	Yes	Yes	Yes	Yes
Housing	Individual	Individual	Individual	Individual	Individual	Individual
PR schedule	Linear (+4)	Linear (+4)	Exponential then linear (+12)	Exponential then linear (+12)	Exponential then linear (+12)	Linear (+4)
Reward	Milkshake	Milkshake	Sucrose	Sucrose	Milkshake	Sucrose
FR max rewards	30	30	—	—	—	30
FR session length	60 min	60 min	45 min	45 min	45 min	60 min
PR session length	60 min	60 min	120 min	120 min	120 min or 360 min	60 min
Timeout	5 min	5 min	—	—	—	5 min
Progression criterion	# of sessions and > 3:1 target‐to‐blank touches	# of sessions and > 3:1 target‐to‐blank touches	# of sessions and > 3:1 target‐to‐blank touches	# of sessions and > 3:1 target‐to‐blank touches	< 20% variation in at least four sessions	# of sessions and > 3:1 target‐to‐blank touches
WT N	12	15	8	15	10	12
NL3 N	10	16	10	14	10	15
# of PR Sessions	6 consecutive	4 consecutive	15 staggered	6 staggered	3 staggered	6 consecutive
Age at testing	30–36 weeks	27–35 weeks	10–27 weeks	9–18 weeks	10–18 weeks	22–25 weeks
Progression	Whole cohort	Whole cohort	Whole cohort	Whole cohort	Individual	Whole cohort
Prior training	rCPT	rCPT	Naïve	Naïve	Naïve	Naïve
Data in figures	1 and 2	3 and 4	5	6	8	9 and 10

*Note:* Details of the various fixed ratio (FR)/progressive ratio (PR) experiments in the order they appear in the paper. Timeout represents the early termination of the session triggered by the absence of responses or head entries to the reward port in the presence of a stimulus for the stated time.

Abbreviations: FR, fixed ratio; NL3, neuroligin‐3 R451C mouse model; PR, progressive ratio; rCPT, rodent‐adapted continuous performance test; WT, wild type.

### 2.3 Touchscreen Pre‐Training

At 9–10 weeks, naïve mice were habituated to the touchscreen chambers for 20 min by delivering a free reward every 10 s. Subsequently, mice were trained to respond to a white square stimulus presented centrally on the screen to elicit a 3× reward delivery (21 μL). Alternatively, if no response was elicited 30 s post‐stimulus presentation, a 1× reward was delivered (7 μL). Mice were given 60 min to complete a maximum of 30 trials, and mice progressed once all trials elicited a response. Mice with prior experience of touchscreen testing skipped pre‐training but were untested for at least 1 week before switching tasks, details of which are provided in Table 1.

### 2.4 Touchscreen Fixed Ratio

A white square stimulus was illuminated within the central touchscreen window. Mice were required to touch the stimulus to elicit a 20 μL reward. The number of touches required to elicit a single reward (the ratio) was set by the schedule, with FR1 requiring one response per reward, FR3 requiring three responses per reward, and so on. The schedule composition of each experiment is outlined in Table 1, and the timelines are depicted in their respective figures. Following intervening touches, the stimulus was removed for a 0.5 s inter‐trial interval (ITI) and elicited a 3 Hz tone for 10 ms. On completion of each ratio (e.g., on the fifth response of an FR5 schedule), the final touch instead triggered the removal of the stimulus, a 3 Hz tone for 1 s, 1× reward delivery, and dim illumination of the reward tray. Following collection of the reward and a subsequent 4.5 s delay, the schedule would then enter a 0.5 s ITI, and the next trial would begin. Mice were given up to 60 min to earn a maximum of 30 rewards. The progression criterion for touchscreen FR involved completing a predetermined number of sessions for each stage [42] and demonstrating a > 3:1 discrimination ratio between stimulus to blank screen touches.

### 2.5 Touchscreen Progressive Ratio

Touchscreen PR utilised a linear +4 PR schedule, with the first ratio = 1 and each subsequent ratio requiring +4 responses [37]. Mice were given the full 60 min to earn unlimited rewards. However, if no responses or entries to the reward magazine were made in the presence of a stimulus for a period of 5 min, mice were timed out and the schedule was terminated. Breakpoint was therefore determined as either the last ratio a mouse completed (i) prior to timeout or (ii) after 60 min.

### 2.6 Lever Chambers

The automated lever apparatus (Med Associates Inc., Virginia, USA) consists of a rectangular box (53.34 cm × 34.93 cm × 27.31 cm) composed of two metal and two clear acrylic walls inside a sound‐attenuated container. On one metallic wall of the chamber were two lever modules equidistant from the midpoint that extended a small operant lever when activated. Above each lever was a dim cue light. A metal reward tray delivered 20 μL of either 5% w/v sucrose or strawberry milk inside a recessed magazine, experimental details for which are outlined in Table 1. For most lever experiments, the reward tray was situated in the center of the chamber wall between the levers. In FR/PR Cohort 5, however, mice were randomly assigned to either levers beside the reward tray (beside) or on the opposing chamber wall (opposite). These data are presented separated into opposite and beside conditions.

### 2.7 Lever Fixed Ratio

In the initial stage of lever FR, one of the two levers was designated active and extended throughout the session. Mice were randomly assigned within genotypes to either a right or a left active lever location and then trained to press the lever to receive a 20 μL reward during a 45‐min session. In the next stage, the second lever was designated inactive, and both levers were extended throughout the session, but only active lever presses led to reward delivery. In subsequent FR stages, the number of active lever presses required to elicit a single reward was increased (Figures 5A, 6A and 8A). Mice in FR/PR Cohort 3 and Cohort 4 were progressed through FR stages after completing a predetermined number of sessions, while mice in FR/PR Cohort 5 were progressed when individual active lever responses were stable (< 20% variation) over four consecutive sessions (Table 1). FR data for FR/PR Cohort 5 are represented as the four sessions per mouse that satisfied the progression criterion.

### 2.8 Lever Satiation Probes

Mice were provided unlimited access to 5% w/v sucrose for 1 h prior to testing. During the subsequent 45‐min test session, both levers were inactive and did not elicit reward delivery.

### 2.9 Lever Progressive Ratio

In lever PR, the number of active lever responses required to elicit a single reward increased at an exponential rate for the first 22 rewards in a session, followed by increments of 12 for all subsequent rewards ([38]). Sessions were either 2 or 6 h long (Table 1). An automatic timeout was not included during lever PR.

### 2.10 Progressive Ratio Drug Probes

Prior to the drug probes, all mice received 3 days of saline (10 mL/kg) delivered i.p. to acclimatise them to restraint and i.p. injection. Mice were then pseudo‐randomly assigned within genotypes to an equal number of control and test days across drug probe sessions. Mice assigned to the test group for that probe day received 1 mg/kg ATO, 3 mg/kg ATO (Sigma Aldrich, Missouri, USA) or 3 mg/kg MPH (Tocris, Bristol, UK) administered intraperitoneally (i.p.), immediately prior to being placed in the experimental chamber. Mice assigned to the control group similarly received an identical volume of saline (10 mL/kg) delivered i.p. immediately prior to testing. All mice were then tested using the PR schedule.

### 2.11 Three‐Compartment Chamber and Group‐Assigned Pairings

The CPP apparatus (Lafayette Instruments, Indiana, USA) consisted of a three‐compartment chamber: two dimly lit main chambers (80 lx) separated by a small bright central chamber (380 lx). The main chambers were distinguishable by their high‐contrast wall patterns: spirals or vertical stripes. The location of these patterns was randomly assigned in a counterbalanced manner within genotypes to either the left or the right chamber. Mice were first placed into the central chamber and allowed 30 min unrestricted access to habituate. Mice that exhibited an initial preference (> 55% time spent) for one chamber were assigned the opposite chamber for cocaine pairing. However, mice that did not display a preference for either chamber (45%–55% total time spent in each chamber) were pseudo‐randomly assigned a wall pattern for cocaine pairing to counterbalance across genotypes. Infrared beams tracked the movement of the mouse within the chambers using the MotorMonitor software (Kinder Scientific, CA, USA).

### 2.12 Cocaine‐Induced Conditioned Place Preference

During conditioning, mice were administered alternating i.p. injections of saline (Sessions 2, 4, 6 and 8) or 20 mg/kg cocaine (Sessions 3, 5, 7 and 9; Johnson Matthey, Edinburgh, UK), as previously described [39]. Mice were then immediately confined to either their saline‐assigned or cocaine‐assigned chamber, respectively, for 30 min. On the test day (Session 10), mice were given unrestricted access to the full apparatus for 5 min. Mice that demonstrated greater than 20% place preference for their cocaine‐paired chamber were progressed to the extinction phase.

### 2.13 Extinction and Reinstatement of Place Preference

Mice were administered saline i.p. for four consecutive sessions immediately before being confined to their previously cocaine‐paired chamber for 30 min. On the fifth session, mice were given unrestricted access to all three chambers for 5 min to probe for an extinguished place preference. The CPP was deemed extinguished if mice spent < 20% more time in the prior cocaine‐paired chamber compared to the previously saline‐paired. Mice that successfully demonstrated extinction progressed to reinstatement, while those that retained their preference were given two further saline‐paired sessions followed by another test session until their CPP was successfully extinguished.

During reinstatement, mice were administered a priming dose of cocaine (10 mg/kg i.p.). Mice were then immediately placed into the central chamber and allowed 5 min to explore all three chambers. Mice were deemed to have reinstated if they spent > 20% more time in the previously cocaine‐paired chamber than in the previously saline‐paired chamber. Mice that underwent reinstatement were then retired from the study.

### 2.14 Task Metrics

For lever experiments, the total number of active and inactive lever presses over the session were analysed. Similarly, in touchscreen experiments, target touches and blank touches were extracted. The discrimination ratio, or ratio of blank over total touches, was also calculated and represented as a percentage of incorrect responding. The time between reward delivery and reward retrieval is termed the reward collection latency, while magazine entries refer to the insertion of the mouse's head into the reward tray (or ‘magazine’) of the touchscreen chamber. Reward collection latencies were non‐parametric, and thus medians were used to represent each mouse, before being averaged across groups. Due to the timeout and maximum reward cap specific to the touchscreen schedule, these metrics, along with total magazine entries, were divided by the total task time for each mouse and analysed as rates (count per second). Lastly, breakpoint (as defined above) was extracted for all touchscreen PR sessions. In the CPP experiment, the total distance traveled and the place preference were plotted. The place preference was calculated by dividing the time spent in the cocaine‐paired chamber by the time spent in the saline‐paired chamber. The number of extinction trials required to successfully extinguish place preference across genotypes was also plotted.

### 2.15 Exclusion

Two mice were excluded from the study. One WT mouse failed to acquire a place preference following cocaine conditioning and was excluded from subsequent extinction (Figure 7). Another WT mouse was excluded during FR5 training following complications from i.p. injections (Figure 6). All data generated prior to their exclusion was included for statistical analysis and visualisation. Further, some of the lever chambers used to generate Figure 6 contained inactive levers that remained in the decompressed position after being extended. Erroneous maximum responses were therefore recorded within the first 5 min of the session. These inactive lever presses were removed prior to analysis and visualisation.

### 2.16 Statistical Analysis

Statistical analyses were performed using RStudio 2023.03.1 and the following packages: lme4 version 1.1.35.1, lmerTest version 3.1.3, dplyr version 1.1.4, glmmTMB version 1.1.9, multcomp version 1.4.25, multcompView version 0.1.10, gamlss version 5.4.22, survival version 3.5.5, survminer version 0.4.9, and emmeans version 1.10.0.

Data containing repeated measures were analysed using generalised linear mixed models (GLMMs) clustered by subject, while single‐measure data were analysed using generalised linear models (GLMs). The GLM/GLMM distribution family was selected based on the type and distribution of the dependent variable, as well as the overall experimental design. Post hoc testing was corrected for multiple comparisons using Tukey's honest significant difference test. No outliers were removed.

For count and discrete data, Poisson's GLM/GLMMs were first performed. This included active lever presses, inactive lever presses, and the number of extinction trials. Dispersion (an assumption of the Poisson model) was subsequently calculated and, where overdispersion was identified, negative binomial distribution models were performed instead. For rate and proportion data bounded between 0 and 1, beta GLM/GLMMs were performed. Where the data contained zeros, the beta models were adjusted for zero inflation. This included blank touch rates, target touch rates, magazine entry rates, and discrimination ratio. As described in the literature [43], response time data were right‐skewed. Inverse Gaussian GLM/GLMMs with log transformations were therefore performed to analyse reward collection latencies. Lastly, for normally distributed continuous data, Gaussian GLM/GLMMs were performed. This included place preference and total distance. Normality was determined using the Shapiro–Wilk test.

### 2.17 Figures

All figures were generated in Prism 10.2.1. Behavioural apparatus diagrams were generated using Canva (CPP) and BioRender.com (lever and touchscreen; [24]; https://BioRender.com/l81m903).

## 3 Results

### 3.1 FR/PR Cohort 1: Touchscreen Chambers With High‐Effort FR

**FIGURE 1:**
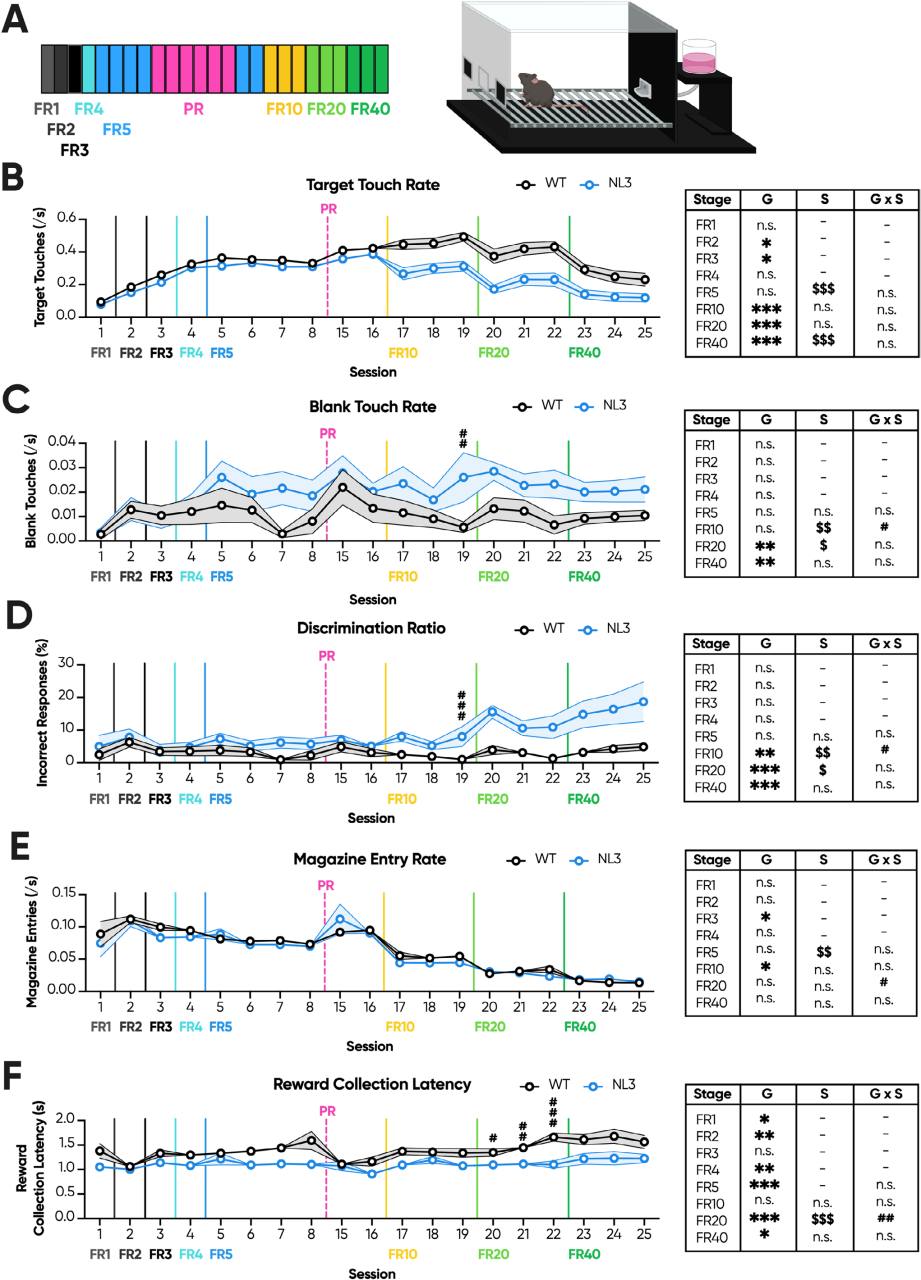
NL3 mice with prior training exhibit reduced responding during higher effort touchscreen fixed ratio. NL3 (*n* = 10) and WT (*n* = 12) mice underwent touchscreen FR, with expedited training due to their prior experience with the task apparatus. (A) This paradigm consisted of 25 sessions total with one FR1, one FR2, one FR3, one FR4 and four FR5 sessions prior to PR. PR then consisted of 6 consecutive sessions, followed by two baseline FR5 sessions, as well as three FR10, three FR20 and three FR40 sessions. (B) NL3 mice exhibited reduced target touch rates across most FR stages, though it was most pronounced at higher effort stages. (C) Similarly, NL3 mice had an elevated blank touch rate at higher effort FR stages, and (D) their discrimination ratio was accordingly impaired. (E) Meanwhile, there were small and inconsistent increases in magazine entry rate in NL3 mice. (F) However, NL3 mice exhibited comparable or even reduced collection latencies compared to WT mice. Solid vertical lines represent the transition from one FR stage to the next. Dashed vertical lines represent PR sessions that are represented elsewhere. All graphs are represented as mean ± SEM. ‘*’ denotes a significant genotype effect, ‘$’ denotes a significant session effect, and ‘#’ denotes a significant genotype by session interaction effect. FR, fixed ratio; G, genotype effect; G × S, genotype by session interaction; NL3, neuroligin‐3 R451C mouse model; PR, progressive ratio; S, session effect; WT, wild type. *p < 0.05, **p < 0.01, ***p < 0.001, ^$^ *p* < 0.05, ^$$^ *p* < 0.01, ^$$$^ *p* < 0.001, ^#^ *p* < 0.05, ^##^ *p* < 0.01, ^###^ *p* < 0.001, n.s. = not significant.

FR/PR Cohort 1 were trained and assessed using the touchscreen PR task. The overall task spanned 25 sessions, with motivation primarily assessed during six consecutive PR sessions and blocks of high‐effort fixed ratio (FR; Figure 1A). This cohort underwent prior training on a touchscreen task of sustained attention [20]. As such, all mice acquired the FR training rapidly (Figure S1) and were progressed to PR after eight sessions. As achieving maximum rewards resulted in early termination of the FR session, we explored interactions with the conditioned stimulus (hereby target touches) as a function of the total time in session (hereby rate) to quantify individual performance. Prior to PR, NL3 mice exhibited reduced target touch rates during FR2 (Figure 1B; genotype effect, *p* < 0.05) and FR3 (Figure 1B; genotype effect, *p* < 0.05). However, this was not observed during FR1, FR4 or FR5 (Figure 1B). Comparatively, touch rates to the unconditioned regions of the screen (hereby blank touch rates) were not significantly different between genotypes across the early stages of FR training (Figure 1C). Accordingly, the proportion of blank touches to total touches (hereby discrimination ratio) between genotypes did not significantly differ throughout FR training (Figure 1D).

**FIGURE 2:**
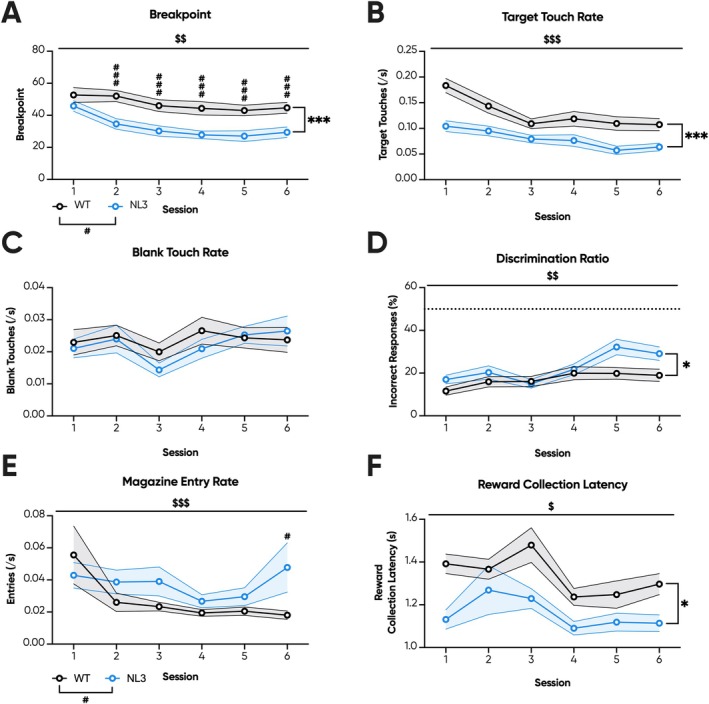
NL3 mice with prior training exhibit reduced responding during consecutive touchscreen progressive ratio. NL3 (*n* = 10) and WT (*n* = 12) mice underwent six consecutive sessions of PR. (A) From Session 2 onwards, NL3 mice exhibited reduced breakpoints, defined as the last ratio performed either prior to timeout for 5 min of inactivity or after 1 h of testing. Throughout PR, NL3 mice had reduced (B) target touch rates, but comparable (C) blank touch rates to WT mice. (D) Their discrimination ratio was accordingly also impaired. NL3 mice exhibited broadly comparable (E) magazine entry rates and reduced (F) reward collection latencies. All graphs are represented as mean ± SEM. ‘*’ denotes a significant genotype effect, ‘$’ denotes a significant session effect, and ‘#’ denotes a significant genotype by session interaction effect. NL3, neuroligin‐3 R451C mouse model; WT, wild type.*p < 0.05, ***p < 0.001, ^$^ *p* < 0.05, ^$$^ *p* < 0.01, ^$$$^ *p* < 0.001, ^#^ *p* < 0.05, ^###^ *p* < 0.001, n.s. = not significant.

Subsequently, mice were progressed to +4 linear PR. NL3 mice exhibited an unaltered breakpoint in the first PR session, but from the second session onwards expressed reduced breakpoints compared to WT mice (Figure 2A; genotype × session interaction, *p* < 0.05; pairwise comparison: Session 1 *p* = 0.26, Session 2 *p* < 0.001, Session 3 *p* < 0.001, Session 4 *p* < 0.001, Session 5 *p* < 0.001, Session 6 *p* < 0.001). Interestingly, there was also a main effect of session on breakpoint regardless of genotype (Figure 2A; session effect, *p* < 0.01), indicating that consecutive PR sessions to some extent deconditions the conditioned stimulus though more readily in NL3 mice. Despite inconsistent breakpoint differences, NL3 mice exhibited significantly reduced target touch rates across all PR sessions (Figure 2B; genotype effect, *p* < 0.001). The transition to PR, however, eliminated genotype differences in blank touch rate (Figure 2C). Thus, the discrimination ratio was also impaired during PR in NL3 mice (Figure 2D; genotype effect, *p* < 0.05).

Following PR, mice were baselined for a further two FR5 sessions, then successively progressed through high‐effort FR in three‐session blocks. These high‐effort conditions also address the cost–benefit decision‐making of mice, though without the ramping demand elasticity of PR. Here, NL3 mice exhibited significantly reduced target touch rates across FR10 (Figure 1B; genotype effect, *p* < 0.001), FR20 (Figure 1B; genotype effect, *p* < 0.001) and FR40 (Figure 1B; genotype effect, *p* < 0.001), with similarly increased blank touch rates across FR20 (Figure 1C; genotype effect, *p* < 0.01) and FR40 (Figure 1C; genotype effect, *p* < 0.01). Accordingly, their discrimination ratio was also increased across FR10 (Figure 1D; genotype effect, *p* < 0.01), FR20 (Figure 1D; genotype effect, *p* < 0.001) and FR40 (Figure 1D; genotype effect, *p* < 0.001), with NL3 mice diverging from WT mice greatest at the higher effort stages.

Interest in the site of reward delivery can also be indicative of interest in the reward itself [37]. Accordingly, NL3 mice exhibited reduced magazine entry rates in two of the FR stages: FR3 (Figure 1E; genotype effect, *p* < 0.05) and FR10 (Figure 1E; genotype effect, *p* < 0.05). No genotype effects were, however, observed in all other FR stages (Figure 1E). Lastly, reward collection times can quantify interest in the reward itself [44]. NL3 mice demonstrated significantly reduced reward collection latencies during FR1 (Figure 1F; genotype effect, *p* < 0.05), FR2 (Figure 1F; genotype effect, *p* < 0.01), FR4 (Figure 1F; genotype effect, *p* < 0.01), FR5 (Figure 1F; genotype effect, *p* < 0.001), FR20 (Figure 1F; genotype effect, *p* < 0.001) and FR40 (Figure 1F; genotype effect, *p* < 0.05) but were indistinguishable from WT mice during FR3 and FR10 (Figure 1F). Meanwhile, in PR, NL3 mice exhibited elevated magazine entry rates only during Session 6 (Figure 2E; genotype × session interaction, *p* < 0.05; pairwise comparison: Session 1 *p* = 0.62, Session 2 *p* = 0.17, Session 3 *p* = 0.14, Session 4 *p* = 0.35, Session 5 *p* = 0.2, Session 6 *p* < 0.05). Similarly, we observed consistently reduced reward collection latencies in NL3 mice throughout PR (Figure 2F; genotype effect, *p* < 0.05). Overall, the PR results in FR/PR Cohort 1 suggest reduced reward motivation and accelerated deconditioning in the NL3 mouse model. However, curiously, consistently reduced reward collection latencies and unaltered or even increased magazine entry rates were at odds with this interpretation.

### 3.2 FR/PR Cohort 2: Touchscreen Chambers With Atomoxetine and Methylphenidate

**FIGURE 3:**
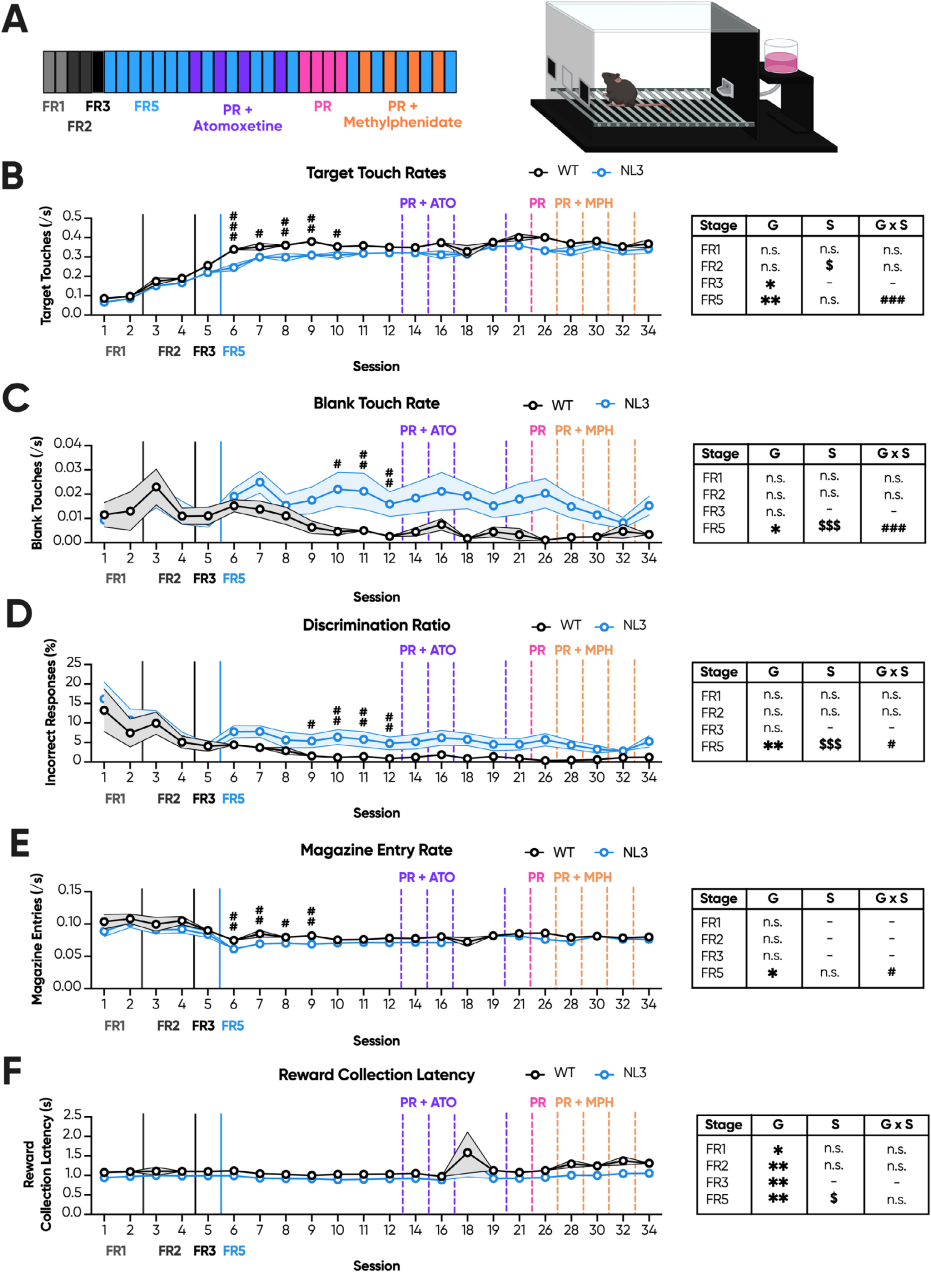
Extending fixed ratio overcame reduced responding in NL3 mice with prior training during higher effort fixed ratio by increasing general responsivity. NL3 (*n* = 16) and WT (*n* = 15) mice rapidly acquired the touchscreen FR/PR experiment due to their prior touchscreen training. However, training was extended to permit stabilisation of conditioned responses prior to progression to PR. (A) This iteration consisted of 34 sessions total with two FR1, two FR2, one FR3, and seven FR5 sessions prior to the first ATO drug probe. Four ATO drug probes were performed with interspersed FR5 sessions to allow for washout of the compound. Following the ATO probes and another FR5 washout session, mice underwent four consecutive PR sessions, before being baselined for one FR5 session and progressed to similarly interspersed PR probes with MPH. (B) NL3 mice exhibited reduced target touch rates compared to WT mice during early FR5 training, but this was attenuated by extending FR5 training. (C) Conversely, blank touch rates were elevated in NL3 mice during the later FR5 stages, (D) coinciding with a reduction in the discrimination ratio. (E) NL3 mice also exhibited reduced magazine entry rates early in FR training and (F) a consistent reduction in reward collection latency across FR training. Solid vertical lines represent the transition from one FR stage to the next. Dashed vertical lines represent PR sessions that are represented elsewhere. All graphs are represented as mean ± SEM. ‘*’ denotes a significant genotype effect, ‘$’ denotes a significant session effect, and ‘#’ denotes a significant genotype by session interaction effect. FR, fixed ratio; G, genotype effect; G × S, genotype by session interaction; NL3, neuroligin‐3 R451C mouse model; PR, progressive ratio; S, session effect; WT, wild type. *p < 0.05, **p < 0.01, ^$^ *p* < 0.05, ^$$$^ *p* < 0.001, ^#^ *p* < 0.05, ^##^ *p* < 0.01, ^###^ *p* < 0.001, n.s. = not significant.

Having identified evidence of reduced motivation, we next sought to ameliorate this deficit using two pharmacological interventions: ATO and MPH. We therefore progressed a second cohort of mice through the touchscreen FR/PR protocol. This iteration spanned 45 sessions in total with 4 consecutive PR sessions, 4 ATO probes and 4 MPH probes (Figure 3A). FR/PR Cohort 2 were similarly trained prior in a touchscreen task of sustained attention [24]. Accordingly, they also readily acquired the training stages of the PR touchscreen task (Figure S1). However, given the significant session effect observed during FR5 in the prior cohort (Figure 1B; session effect, *p* < 0.001), the FR5 training stage was extended until target touch rates stabilised across genotypes. As observed in the prior cohort, target touch rate was intermittently reduced in NL3 mice across FR training stages, including FR3 (Figure 3B; genotype effect, *p* < 0.05) and FR5 (Figure 3B; genotype effect, *p* < 0.01) but not FR1 or FR2 (Figure 3B). During the extended FR5 training, a genotype by session interaction effect emerged, with NL3 mice exhibiting reduced target touch rates only in the first five sessions (Figure 3B; genotype × session interaction, *p* < 0.001; pairwise comparison: Session 1 *p* < 0.001, Session 2 *p* < 0.05, Session 3 *p* < 0.01, Session 4 *p* < 0.01, Session 5 *p* < 0.05, Session 6 *p* = 0.06, Session 7 *p* = 0.19). In the final two FR5 sessions prior to progression to PR, this genotype effect was absent as target touch rates stabilised. Similarly, the blank touch rate in NL3 mice remained elevated across FR3 (Figure 3C; genotype effect, *p* < 0.05) and intermittently throughout FR5 prior to progression to PR (Figure 3C; genotype × session interaction, *p* < 0.05; pairwise comparison: Session 1 *p* = 0.8, Session 2 *p* = 0.12, Session 3 *p* = 0.95, Session 4 *p* = 0.08, Session 5 *p* < 0.05, Session 6 *p* < 0.01, Session 7 *p* < 0.01). A genotype effect was not, however, observed during FR1 or FR2 (Figure 3C). Accordingly, and in line with the prior cohort, NL3 mice exhibited no differences in their discrimination ratio during FR1, FR2, or FR3 (Figure 3D). However, a genotype by session interaction effect was observed during FR5, with significant impairment in NL3 mice only in the last four FR5 sessions, coinciding with the increase in blank touch responses and the amelioration of differences in target touches (Figure 3D; genotype × session interaction, *p* < 0.05; pairwise comparison: Session 1 *p* = 0.27, Session 2 *p* = 0.05, Session 3 *p* = 0.34, Session 4 *p* < 0.05, Session 5 *p* < 0.01, Session 6 *p* < 0.01, Session 7 *p* < 0.01). NL3 mice also intermittently exhibited reduced magazine entry rates for the first 4 sessions of FR5 (Figure 3E; genotype × session interaction, *p* < 0.05; pairwise comparison: Session 1 *p* < 0.01, Session 2 *p* < 0.01, Session 3 *p* < 0.05, Session 4 *p* < 0.01, Session 5 *p* = 0.24, Session 6 *p* = 0.29, Session 7 *p* = 0.12), but otherwise were comparable to WT mice across FR1, FR2, and FR3 (Figure 3E). Overall, these results suggest that NL3 mice take longer to stabilise their FR5 performance and do so by increasing overall responsivity. Meanwhile, reward collection latency was again significantly reduced in NL3 mice across all FR stages, including FR1 (Figure 3E; genotype effect, *p* < 0.05), FR2 (Figure 3E; genotype effect, *p* < 0.01), FR3 (Figure 3E; genotype effect, *p* < 0.01), and FR5 (Figure 3E; genotype effect, *p* < 0.01).

**FIGURE 4:**
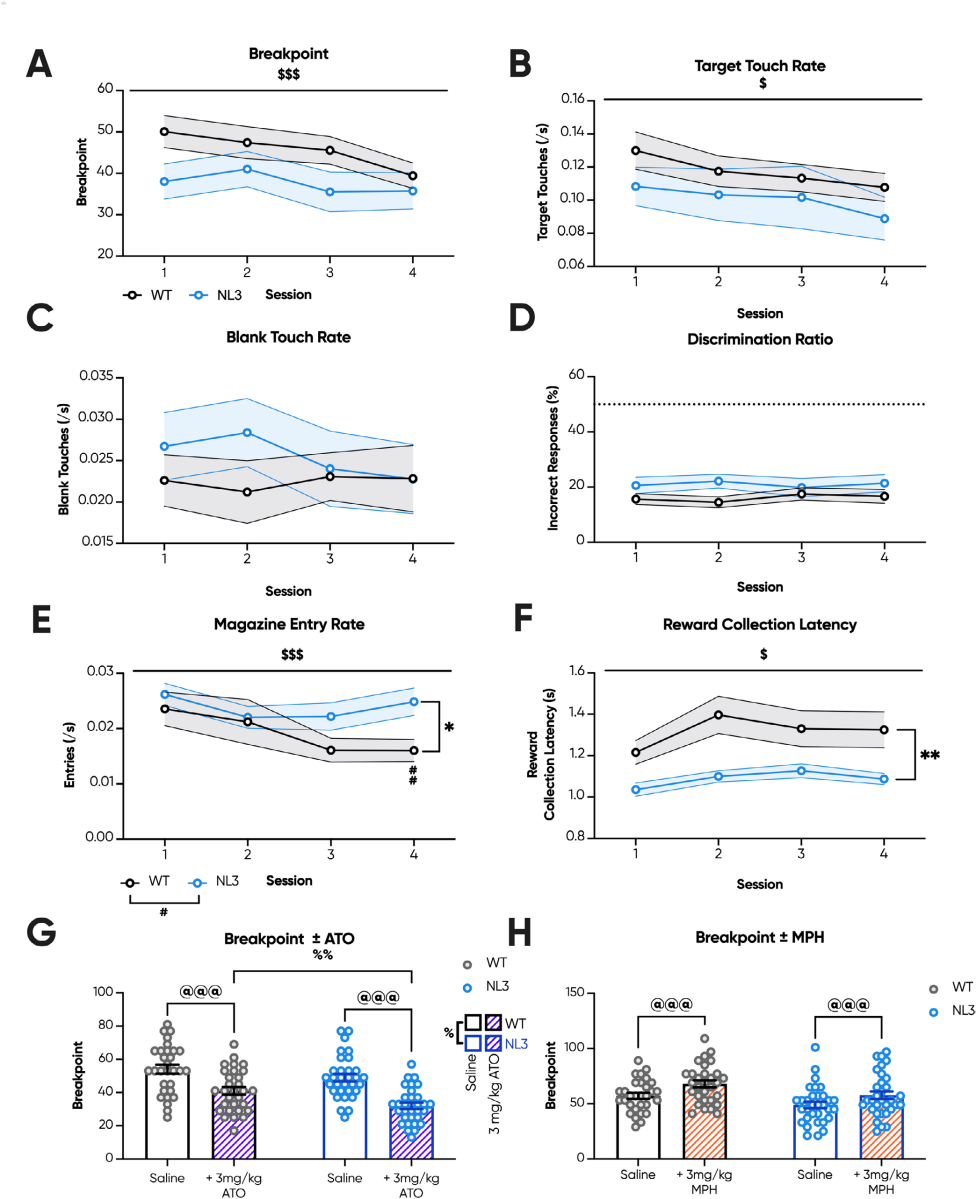
Extending fixed ratio training in NL3 mice with prior training attenuated progressive ratio performance. NL3 (*n* = 16) and WT (*n* = 15) mice underwent four consecutive sessions of PR, as well as PR probes with intraperitoneal injections with saline and either 3 mg/kg ATO or 3 mg/kg MPH. There were no genotype differences in (A) breakpoint, (B) target touch rate, (C) blank touch rate, and (D) discrimination ratio between NL3 and WT mice. Meanwhile, (E) magazine entry rates were elevated in the final session, whereas (F) reward collection latencies were reduced in the NL3 mouse model throughout PR. (G) 3 mg/kg ATO reduced breakpoint across genotypes, though to a lesser degree in NL3 mice compared to WT mice, whereas (H) 3 mg/kg MPH increased breakpoint to a comparable degree in both genotypes. All graphs are represented as mean ± SEM. ‘*’ denotes a significant genotype effect, ‘$’ denotes a significant session effect, ‘#’ denotes a significant genotype by session interaction effect, ‘@’ denotes a significant drug effect, and ‘%’ denotes a significant genotype by drug interaction effect. ATO, atomoxetine; MPH, methylphenidate; NL3, neuroligin‐3 R451C mouse model; WT, wild type. *p < 0.05, **p < 0.01, ^$^ *p* < 0.05, ^$$$^ *p* < 0.001, ^#^ *p* < 0.05, ^##^ *p* < 0.01, ^@@@^ *p* < 0.001, ^%^ *p* < 0.05, ^%%^ *p* < 0.01.

Strikingly, unlike the prior cohort, NL3 mice were comparable to WT mice during all four consecutive PR sessions across breakpoint (Figure 4A), target touch rate (Figure 4B), blank touch rate (Figure 4C) and discrimination ratio (Figure 4D). Magazine entry rates were elevated in NL3 mice only during the final PR session (Figure 4E; genotype × session interaction, *p* < 0.05; pairwise comparison: Session 1 *p* = 0.3, Session 2 *p* = 0.49, Session 3 *p* = 0.05, Session 4 *p* < 0.01), while there was a significant reduction in reward collection latency throughout PR (Figure 4F; genotype effect, *p* < 0.01). Similarly, during both drug trials, no genotype effect was observed on breakpoint between the saline‐treated groups (Figure 4G,H). Both genotypes, however, experienced significant drug effects, with 3 mg/kg ATO decreasing breakpoint (Figure 4G; drug effect, *p* < 0.001; pairwise comparison: WT saline—WT ATO *p* < 0.001, NL3 saline—NL3 ATO *p* < 0.001) and 3 mg/kg MPH increasing breakpoint (Figure 4H; drug effect, *p* < 0.001; pairwise comparison: WT saline—WT MPH *p* < 0.001, NL3 saline—NL3 MPH *p* < 0.001). Intriguingly, the effect of 3 mg/kg ATO at reducing breakpoint was significantly greater in NL3 mice than in WT mice (Figure 4H; genotype × drug interaction, *p* < 0.05; pairwise comparison: saline WT—KI *p* = 0.3, ATO WT—KI *p* < 0.01).

### 3.3 FR/PR Cohort 3: Lever Chambers With Atomoxetine and High‐Effort FR

Given the conflicting results from FR/PR Cohorts 1 and 2, we next sought to limit the influence of potential confounding variables. We opted therefore to use a naïve cohort of mice and to follow a more widely used lever chamber protocol [38]. We retained the delivery of ATO given its predictive validity in modifying response patterns across humans and mice [33, 36].

**FIGURE 5:**
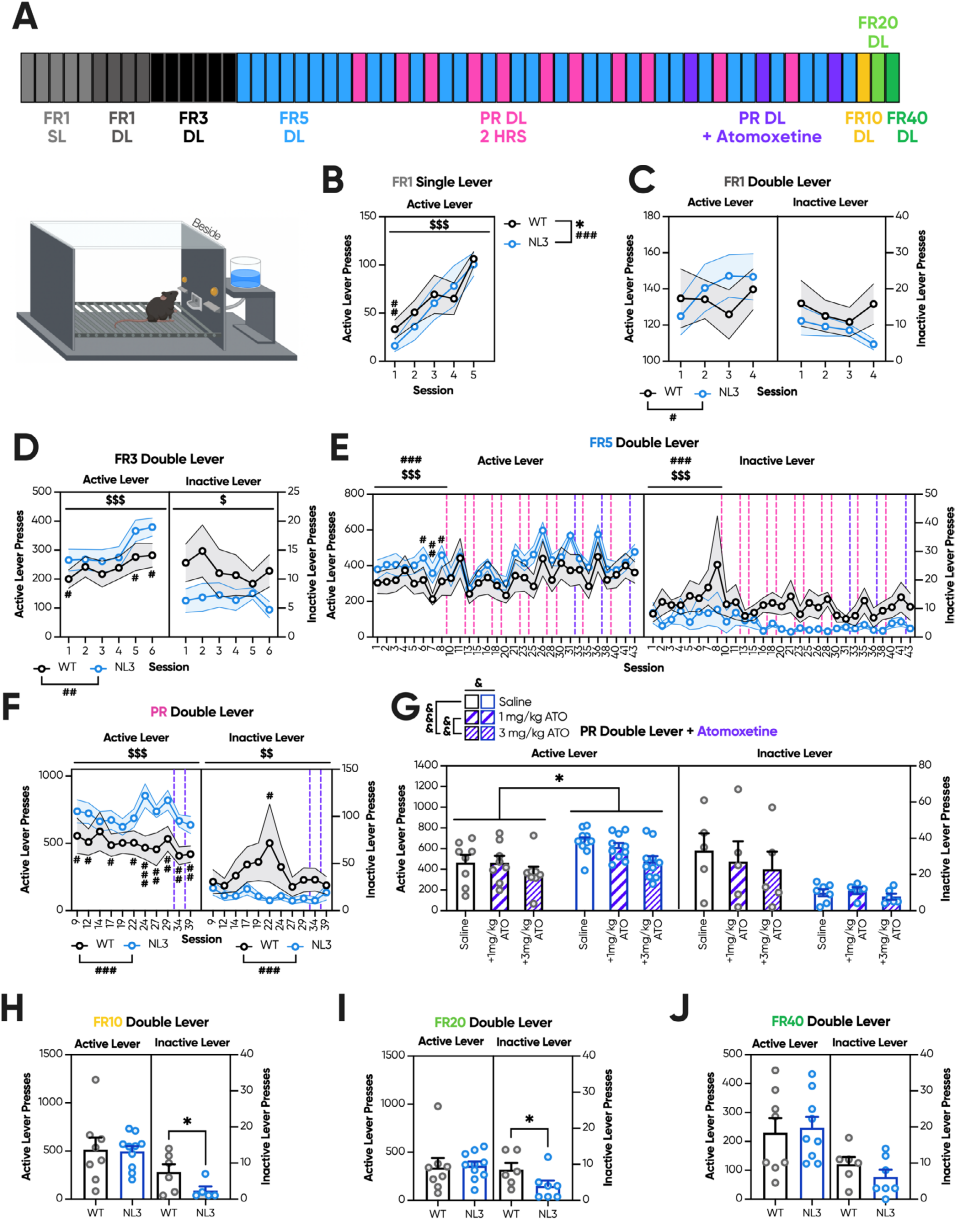
Naïve NL3 mice exhibit elevated fixed and progressive ratio responding following lever‐based operant training. Naïve NL3 (*n* = 10) and WT (*n* = 8) mice underwent a lever FR/PR schedule. (A) This paradigm consisted of 64 sessions in total with five FR1 SL, four FR1 DL, six FR3 and eight FR5 sessions prior to the first PR session. Thereafter, mice were progressed to a total of 16 PR sessions, four of which involved the intraperitoneal delivery of saline, 1 mg/kg ATO or 3 mg/kg ATO. These injection groups were counterbalanced across genotype and session. Following the final ATO drug probe, mice were assessed in a series of ramping high‐effort FR sessions: one FR10, one FR20 and one FR40. The operant apparatus was set up in the standard configuration with the active and inactive levers positioned either side of the reward magazine. (B) During FR1 SL, NL3 mice interacted less with the active lever during the first session, but otherwise demonstrated similar learning trajectories. (C) In FR1 DL, there were no genotype effects at the active or inactive lever, with task performance stabilising across the FR1 stages. (D) NL3 mice performed elevated active lever presses in the initial and two last sessions of FR3 training, though inactive lever presses remained rare and unaffected by genotype. (E) In FR5, prior to PR, there were no genotype effects in active lever presses for the first six sessions. However, NL3 mice exhibited elevated active lever responding for the final two sessions prior to the first PR session. (F) This elevated responding was carried forward into PR with intermittently increased active lever presses in NL3 mice during the initial PR sessions. Starting from the seventh PR session, NL3 mice experienced a spike in active lever responding, producing a larger genotype effect during the subsequent PR trials. (G) As such, mice progressed to the ATO probes at this late stage exhibited a strong genotype difference in active and inactive lever responding, even within the saline‐injected controls. Despite this, there was still a dose‐dependent drug effect, with 3 mg/kg ATO but not 1 mg/kg ATO reducing active lever responding. NL3 mice exhibited reduced inactive lever presses during (H) FR10 and (I) FR20 but no such effect was observed during (J) FR40. Dashed vertical lines represent PR sessions that are represented elsewhere in the figure. All graphs are represented as mean ± SEM. ‘*’ denotes a significant genotype effect, ‘$’ denotes a significant session effect, ‘#’ denotes a significant genotype by session interaction effect, and ‘&’ denotes a significant dose effect. ATO, atomoxetine; FR, fixed ratio; HRS, hours; NL3, neuroligin‐3 R451C mouse model; PR, progressive ratio; WT, wild type. *p < 0.05, ^$^ *p* < 0.05, ^$$^ *p* < 0.01, ^$$$^ *p* < 0.001, ^#^ *p* < 0.05, ^##^ *p* < 0.01, ^###^ *p* < 0.001, ^&^ *p* < 0.05, ^&&^ *p* < 0.01, ^&&&^ *p* < 0.001, n.s. = not significant.

In this PR iteration, all mice were trained for five FR1 sessions with a single active lever (SL), four FR1 sessions with a double lever (DL; one active and one inactive lever), six FR3 sessions, and eight FR5 sessions prior to progression to 16 interspersed PR sessions (Figure 5A). Four of the PR sessions involved the additional delivery of saline, 1 mg/kg ATO or 3 mg/kg ATO. The final three sessions performed by this cohort were a series of ramping high‐effort FRs. Unlike the prior touchscreen experiments, sessions were consistent in length for all mice; thus, lever presses have not been transformed to rates.

In FR1 SL training, NL3 mice initially interacted with the lever less in the first session but achieved performance similar to WT mice by the second session (Figure 5B; genotype × session interaction, *p* < 0.001; pairwise comparison: Session 1 *p* < 0.01, Session 2 *p* = 0.13, Session 3 *p* = 0.38, Session 4 *p* = 0.91, Session 5 *p* = 0.53). In both genotypes, there was a steep learning curve across sessions (Figure 5B; session effect, *p* < 0.001). During FR1 DL, active lever responses were stable across sessions, and there was no effect of genotype (Figure 5C). Mice in both genotypes explored the inactive lever to a similar degree, though inactive lever pressing was low overall (Figure 5C). In FR3, there was a significant genotype by session interaction, with higher active lever presses in NL3 mice in the first and two last sessions (Figure 5D; genotype × session interaction, *p* < 0.01; pairwise comparison: Session 1 *p* < 0.05, Session 2 *p* = 0.28, Session 3 *p* = 0.17, Session 4 *p* = 0.27, Session 5 *p* < 0.05, Session 6 *p* < 0.05). Inactive lever presses, however, did not differ across genotypes (Figure 5D). In FR5, prior to progression to PR, a similar pattern emerged. NL3 mice initially were no different from WT mice in active lever presses but exhibited elevated lever pressing in the final three FR5 sessions (Figure 5E; genotype × session interaction, *p* < 0.001; pairwise comparison: Session 1 *p* = 0.13, Session 2 *p* = 0.08, Session 3 *p* = 0.11, Session 4 *p* = 0.57, Session 5 *p* = 0.06, Session 6 *p* < 0.05, Session 7 *p* < 0.01, Session 8 *p* < 0.05). Conversely, inactive lever presses during FR5 identified no genotype effects that survived correction for multiple comparisons (Figure 5E).

During lever PR, no main effect of genotype was observed (Figure 5F); however, a nuanced interaction between genotype and session emerged. Specifically, in PR Sessions 1, 2 and 4, NL3 mice demonstrated significantly higher active lever pressing. This effect reached its peak during PR Session 7, where a marked surge in active lever pressing was recorded within the NL3 mice (Figure 5F; genotype × session interaction, *p* < 0.001; pairwise comparison: Session 1 *p* < 0.05, Session 2 *p* < 0.05, Session 3 *p* = 0.25, session 4 *p* < 0.05, Session 5 *p* = 0.1, Session 6 *p* < 0.05, Session 7 *p* < 0.001, Session 8 *p* < 0.01, Session 9 *p* < 0.01, Session 10 *p* < 0.01, Session 11 *p* < 0.01). Inactive lever presses were, however, largely unaltered, though they were significantly elevated in NL3 mice during Session 6 (Figure 5F; genotype × session interaction, *p* < 0.001; pairwise comparison: Session 1 *p* = 0.77, Session 2 *p* = 0.92, Session 3 *p* = 0.17, Session 4 *p* = 0.85, Session 5 *p* = 0.25, Session 6 *p* < 0.05, Session 7 *p* = 0.27, Session 8 *p* = 0.25, Session 9 *p* = 0.25, Session 10 *p* = 0.13, Session 11 *p* = 0.98). Notably, during the ATO probe sessions that fell within this period of heightened active lever responding, genotype exhibited the strongest impact on active lever presses (Figure 5G; *p* < 0.05), though there was no such effect on inactive lever presses (Figure 5G). Despite the absence of an overall drug effect (Figure 5G), there was a dose‐dependent effect on active lever presses (Figure 5G; dose effect, *p* < 0.05; pairwise comparison: saline—1 mg/kg ATO *p* = 0.97, saline—3 mg/kg ATO *p* < 0.001, 1 mg/kg ATO—3 mg/kg ATO *p* < 0.001) with 1 mg/kg ATO producing no effect, whereas 3 mg/kg ATO reduced active lever presses in both genotypes. In contrast, neither drug nor dose had any impact on inactive lever presses (Figure 5G). Subsequently, during FR10 (Figure 5H; *p* < 0.05) and FR20 (Figure 5I; *p* < 0.05), NL3 mice exhibited lower inactive lever presses compared to WT mice, but this effect was absent in FR40 (Figure 5J). No main effect of genotype was, however, evident in active lever presses across the high‐effort FR stages, including FR10 (Figure 5H), FR20 (Figure 5I), and FR40 (Figure 5J). The lever FR/PR results in naïve mice, therefore, support a third contradictory conclusion: increased motivation in the NL3 mouse model.

### 3.4 FR/PR Cohort 4: Lever Chambers With Satiation Probes and Atomoxetine

Having conflicting PR data to support decreased, unaltered, and increased non‐social motivation in the NL3 mouse model, we next considered variables that may be influencing task performance across cohorts. We thus sought to probe these potential confounders to aid the interpretation of the underlying motivational state. First, we considered that elevated responding by NL3 mice in FR/PR Cohort 3 grew in magnitude over repeated sessions and hypothesised that this may arise from a greater propensity for habitual responding over training. We therefore introduced satiation probes in a fourth cohort to assess responding during outcome devaluation.

**FIGURE 6:**
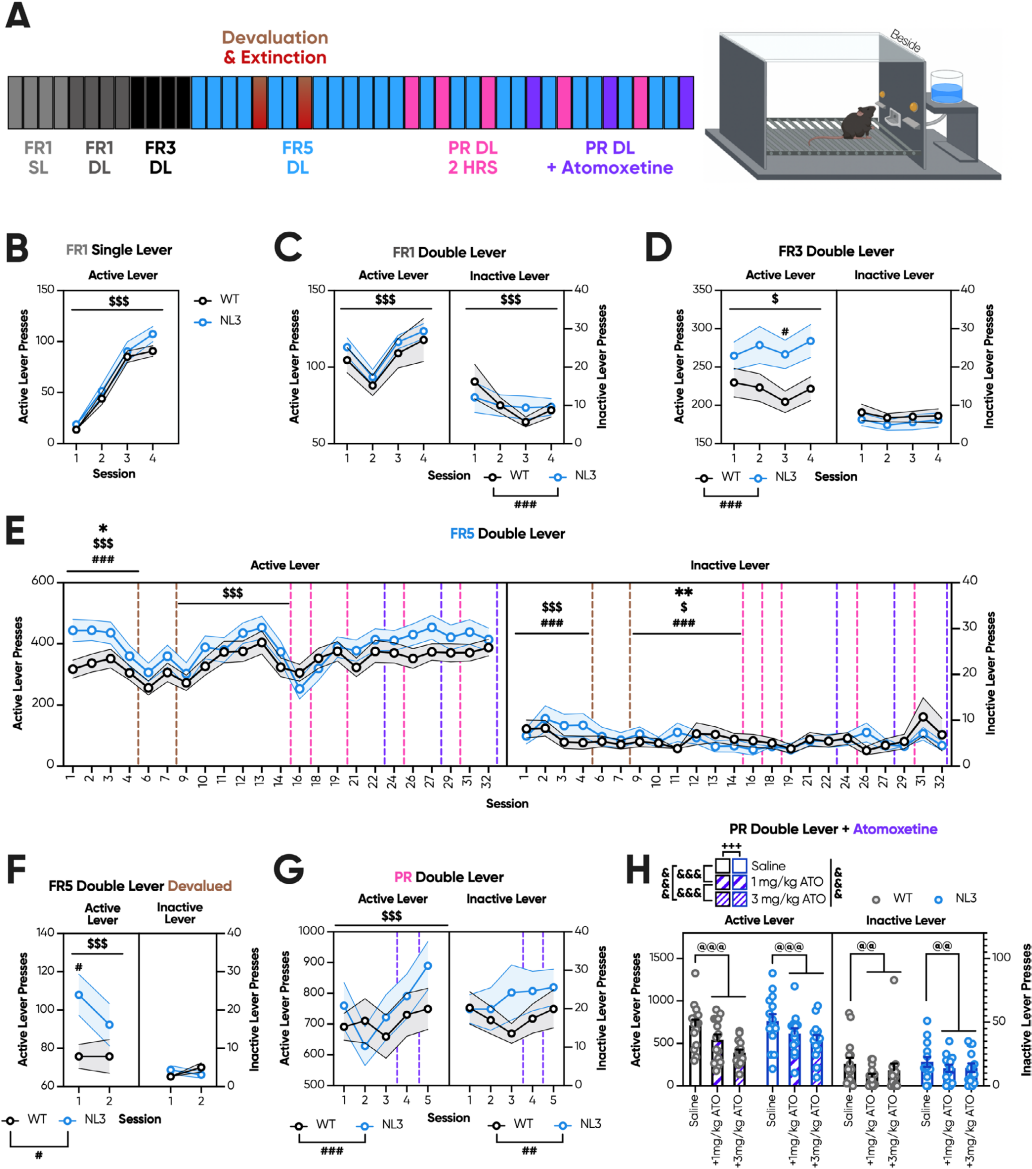
Satiation probes during training and a limited number of sessions prevent habitual lever responding in NL3 mice. Naïve NL3 (*n* = 14) and WT (*n* = 15) mice underwent lever FR/PR testing. (A) In this experiment, mice were trained over 45 sessions, with four FR1 SL, four FR1 DL, four FR3, and four FR5 sessions prior to two satiation probes, with two FR5 sessions in‐between. Mice were then progressed to six sessions of FR5 to baseline before undergoing eight interspersed sessions of PR: Three of which involved intraperitoneal injections of saline, 1 mg/kg ATO or 3 mg/kg ATO. Drug groups were counterbalanced across genotypes and sessions. The lever apparatus was in a standard configuration with the active and inactive levers positioned either side of the reward magazine. (B) In FR1 SL, there were no genotype differences in active lever responding. (C) Similarly, in FR1 DL, active lever responses were unaffected by genotype, but the two genotypes reduced their inactive lever presses at moderately different rates across sessions. (D) During FR3, inactive lever presses were rare and did not exhibit genotype effects, while NL3 mice performed increased active lever presses in the latter portion of FR3 training. (E) This period of elevated active lever presses continued into the first few sessions of FR5, before attenuating prior to the satiation probes. Subsequent FR5 responses were negatively impacted by the satiation probes, but mice were successfully baselined with additional FR5 sessions. (F) During the satiation probes, both genotypes greatly reduced their interaction with the conditioned lever. However, NL3 mice exhibited elevated conditioned lever responding during the first satiation session, though this was absent in the second. (G) During PR, NL3 mice descriptively increased active and inactive lever responses over sessions, though overall genotype effects were absent across the reduced number of PR sessions. (H) Both 1 mg/kg ATO and 3 mg/kg ATO reduced active lever responding, though 3 mg/kg ATO produced larger effect sizes. These drug effects were observed in both WT and NL3 mice. Similarly, both 1 mg/kg ATO and 3 mg/kg ATO reduced inactive lever responding in both genotypes. Dashed vertical lines represent intervening PR sessions that are represented elsewhere in the figure. All graphs are represented as mean ± SEM. ‘*’ denotes a significant genotype effect, ‘$’ denotes a significant session effect, ‘#’ denotes a significant genotype by session interaction effect, ‘&’ denotes a significant dose effect, ‘@’ denotes a significant drug effect, and ‘+’ denotes a significant genotype by dose interaction effect. ATO, atomoxetine; DL, double lever; FR, fixed ratio; HRS, hours; NL3, neuroligin‐3 R451C mouse model; PR, progressive ratio; WT, wild type. *p < 0.05, **p < 0.01, ^$^ *p* < 0.05, ^$$$^ *p* < 0.001, ^#^ *p* < 0.05, ^##^ *p* < 0.01, ^###^ *p* < 0.001, ^&&&^ *p* < 0.001, ^@@^ *p* < 0.01, ^@@@^ *p* < 0.001, ^+++^ *p* < 0.001.

Naïve mice in Cohort 4 were thus trained for a total of 45 sessions, with four FR1 SL, four FR1 DL, four FR3, and four FR5 FR training sessions, followed by two satiation probes. Mice were subsequently baselined at FR5 to mitigate the impact of the satiation sessions on lever conditioning, before being progressed to interspersed PR and ATO drug probes (Figure 6A). No genotype effects at the active or inactive lever were observed over FR1 SL (Figure 6B) or FR1 DL (Figure 6C). During FR3, NL3 mice exhibited increased responding solely in Session 3 (Figure 6D; genotype × session interaction, *p* < 0.001; pairwise comparison: Session 1 *p* = 0.31, Session 2 *p* = 0.09, Session 3 *p* < 0.05, Session 4 *p* = 0.06). No genotype effects were observed at the inactive lever during FR3 or FR5 (Figure 6D,E). Despite complex main and interaction effects, no genotype differences during active lever presses during early FR5 survived correction for multiple comparisons (Figure 6E). During the satiation probes, NL3 mice consumed a comparable quantity of reward to WT mice (data not shown) and exhibited greater conditioned lever responding in Session 1 but not in the subsequent session (Figure 6F; genotype × session interaction, *p* < 0.001; pairwise comparison: Session 1 *p* < 0.05, Session 2 *p* = 0.31). Events at the unconditioned lever during the satiation probes were extremely rare. As such, no genotype effects upon unconditioned lever presses survived multiple comparison correction (Figure 6F; genotype × session interaction, *p* < 0.001; pairwise comparison: Session 1 *p* = 0.2, Session 2 *p* = 0.17). FR5 responding following the satiation probes was reduced in both genotypes. An additional six FR5 sessions were performed to re‐establish a stable active lever association and baseline all mice before progression to PR. During FR5 between devaluation and progression, there were no genotype effects at the active lever (Figure 6E). Once again, the rarity of events at the inactive lever meant all genotype effects did not survive post hoc correction (Figure 6E; session × genotype interaction, *p* < 0.001; pairwise comparison: Session 1 *p* = 0.39, Session 2 *p* = 0.83, Session 3 *p* = 0.06, Session 4 *p* = 0.83, Session 5 *p* = 0.25, Session 6 *p* = 0.54).

During PR, there was a significant genotype by session interaction effect in active lever (Figure 6G; genotype × session interaction, *p* < 0.001; pairwise comparison: Session 1 *p* = 0.54, Session 2 *p* = 0.28, Session 3 *p* = 0.55, Session 4 *p* = 0.62, Session 5 *p* = 0.22) and inactive lever (Figure 6G; genotype × session interaction, *p* < 0.01; pairwise comparison: Session 1 *p* = 0.93, Session 2 *p* = 0.68, Session 3 *p* = 0.1, Session 4 *p* = 0.33, Session 5 *p* = 0.5) responding, though no session survived multiple comparison correction. Descriptively, active lever responding by WT mice remained relatively stable, while NL3 mice exhibited a general upward trend across PR sessions. The number of sessions was intentionally capped at fewer than six in an attempt to avoid the sudden emergence of the strongest genotype effects observed in FR/PR Cohort 3 (Figure 5F). Under these conditions, we identified a significant drug (Figure 6H; drug effect, *p* < 0.001) and dose effect, with 3 mg/kg ATO stronger at reducing active lever responding than 1 mg/kg ATO (Figure 6H; dose effect, *p* < 0.001; pairwise comparison: saline—1 mg/kg ATO *p* < 0.001, saline—3 mg/kg ATO *p* < 0.001, 1 mg/kg ATO—3 mg/kg ATO *p* < 0.001). ATO similarly reduced inactive lever responding (Figure 6H; drug effect, *p* < 0.01), though there were no dose or genotype by drug effects (Figure 6H). Curiously, unlike FR/PR Cohort 3, there was no main effect of genotype at either the active or inactive levers (Figure 6H).

### 3.5 Cocaine‐Conditioned Place Preference

**FIGURE 7:**
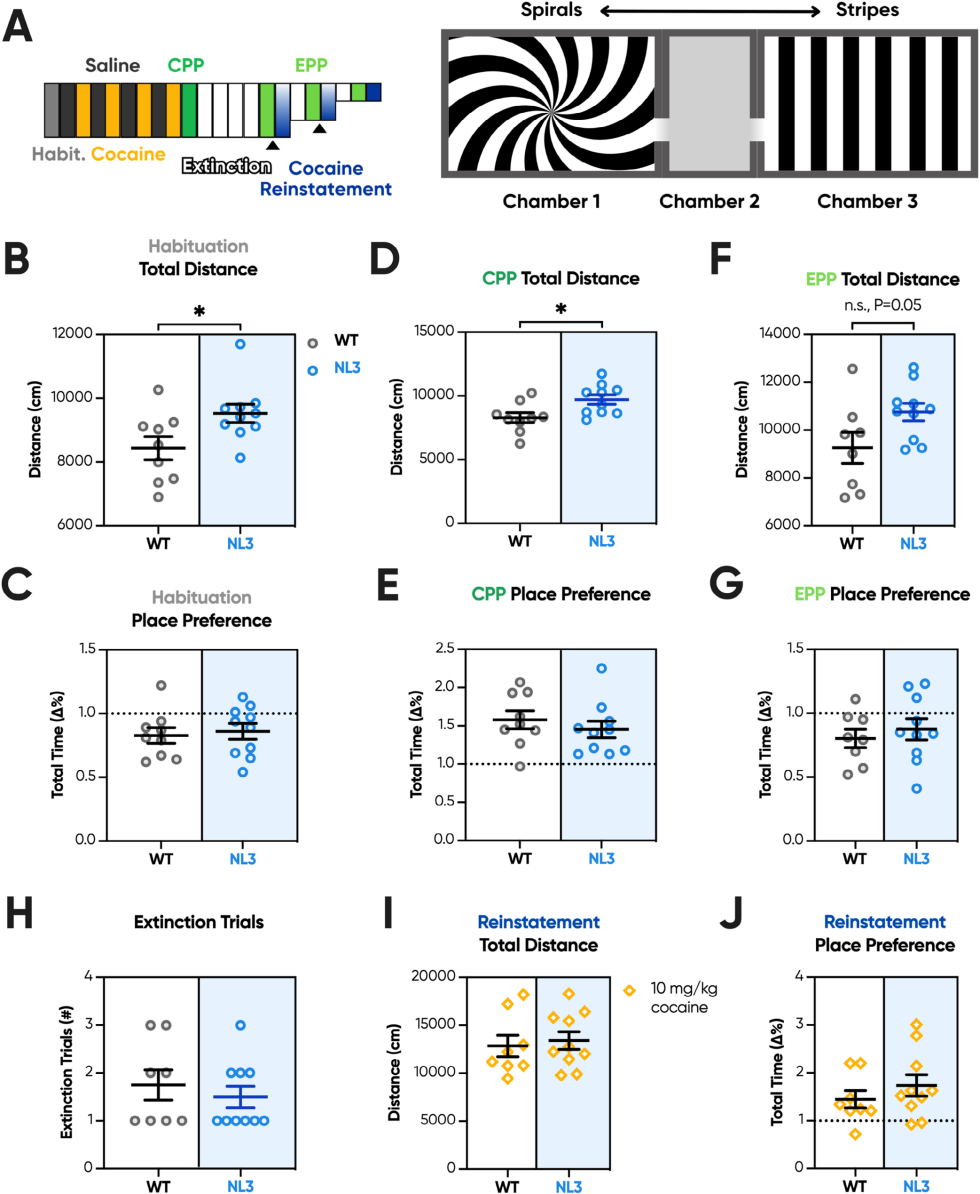
NL3 mice successfully develop a place preference following cocaine conditioning but exhibit hyperactivity. NL3 (*n* = 10) and WT (*n* = 8) mice successfully acquired a conditioned place preference. Mice were pseudo‐randomly assigned within genotypes to a reward‐associated chamber and tested for a minimum of 16 sessions. (A) These sessions included habituation, eight conditioning sessions, a conditioned place preference test, four extinction sessions, a place preference test following extinction, and a reinstatement probe. If mice continued to show a preference for their reward‐associated chamber during the place preference test following extinction, instead of progressing to reinstatement, mice would instead undergo two further extinction sessions before another place preference probe. This cycle occurred twice before all mice in the cohort successfully extinguished their conditioned place preference. The experimental apparatus consisted of a central chamber flanked by two larger chambers, each uniquely marked with high‐contrast patterns: spirals and vertical stripes. NL3 mice were hyperactive across (B) habituation and (D) CPP, with a trend suggesting hyperactivity during (F) the place preference session following extinction as well. (C) However, neither genotype displayed a chamber preference during habituation. (E) Both genotypes also successfully developed a comparable degree of conditioned place preference. (G) Similarly, both genotypes successfully extinguished their place preference and (H) underwent a similar number of extinction trials. (I) When i.p. injected with 10 mg/kg cocaine during reinstatement, both genotypes experienced cocaine‐induced hyperactivity. (J) Both genotypes also reinstated their conditioned place preference after receiving the previously associated reward. All graphs are represented as mean ± SEM. ‘*’ denotes a significant genotype effect. CPP, conditioned place preference; EPP, extinguished place preference; habit., habituation; NL3, neuroligin‐3 R451C mouse model; WT, wild type. *p < 0.05, n.s. = not significant.

Results from FR/PR Cohort 4 therefore supported our hypothesis that NL3 mice more readily form habitual operant responses. As such, we next sought to probe motivation while removing the operant component of the task. A naïve cohort was pseudo‐randomly assigned a wall pattern and a reward‐paired chamber to undergo cocaine CPP (Figure 7A). Notably, in this paradigm, mice are not required to perform an action to elicit the reward. Instead, the proportion of time spent in a reward‐paired chamber versus an unpaired chamber quantifies reward‐seeking behaviour [39]. Overall activity within the task apparatus is plotted alongside place preference to assess general exploratory behaviour.

During habituation, NL3 mice were more active than WT mice (Figure 7B; genotype effect, *p* < 0.05), but like WT mice, did not display a preference for either chamber pattern (Figure 7C). NL3 mice also experienced a similar degree of cocaine‐induced hyperactivity to WT mice during early conditioning sessions but were curiously one session faster to habituate to its locomotor effects (Figure S2). During the CPP test session, mice were assessed for their place preference, and NL3 mice were again hyperactive (Figure 7D; genotype effect, *p* < 0.05), but exhibited a comparable preference for the cocaine‐paired chamber to WT mice (Figure 7E). Following extinction, NL3 mice exhibited a trend towards hyperactivity (Figure 7F; genotype effect, *p* = 0.05), though no difference in their chamber preference (Figure 7G) or the number of trials required to extinguish their place preference (Figure 7H). Mice subsequently underwent a reinstatement probe, where both genotypes experienced drug‐induced hyperactivity (Figure 7I). Similarly, both genotypes re‐established a preference for the previously reward‐associated chamber, suggesting a similar degree of long‐term associative reward memory (Figure 7J).

### 3.6 FR/PR Cohort 5: Lever Chambers With Inverse Response Site

Next, we considered whether physical differences between the touchscreen and lever chambers influence task performance. In conditioning tasks, subjects track cues in their environment and form associations when they predict reward. In our experimental design, both the reward delivery location and the site of the conditioned response exhibit motivational draw, and subjects differ in the ratio to which they find them incentivising. Goal‐trackers, for example, exhibit a bias towards the site of reward delivery, while sign‐trackers fixate on the location of the conditioned stimulus [45]. In the standard touchscreen setup, the reward magazine is located at the opposite end of the chamber to the touchscreen. Meanwhile, in the standard configuration of the lever chamber, levers are located on either side of the reward magazine. Curiously, NL3 mice produced elevated magazine entry rates across touchscreen FR/PR in Cohorts 1 and 2 (Figures 2E and 4E). If NL3 mice exhibit excess goal‐tracking, their proximity to the reward magazine could draw them away from the touchscreen and thus reduce responding. Alternatively, this same phenotype would place NL3 mice in proximity to the levers in the standard lever chamber configuration, potentially driving responding. Despite lacking a reward magazine tracker, we utilised the modularity of the lever chambers to assess goal‐ versus sign‐tracking. Half of the mice in each genotype were trained in a chamber with levers positioned on either side of the reward magazine, while the remaining mice were trained with levers located at the opposite end of the chamber.

**FIGURE 8:**
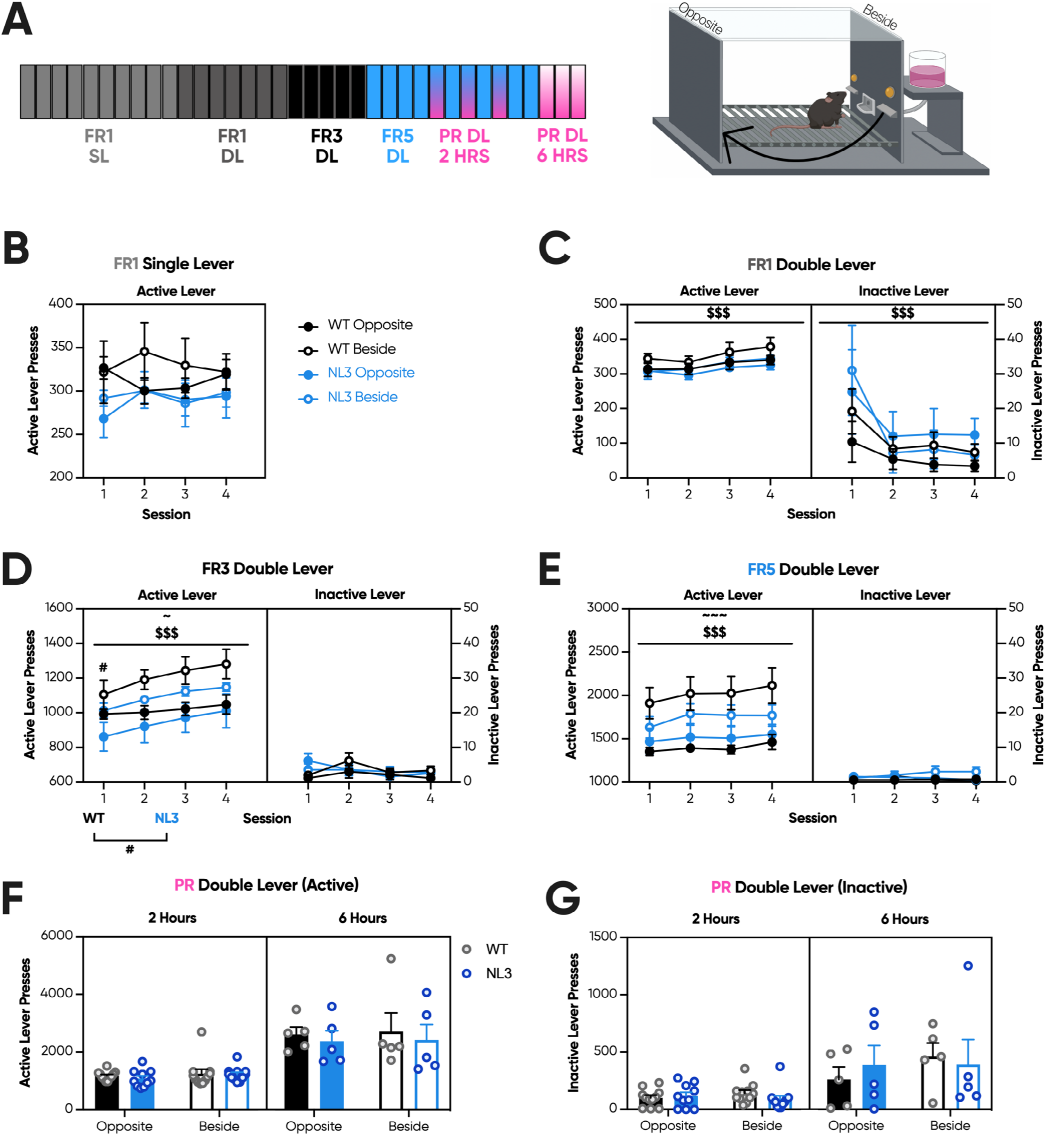
Position of the response site does not alter the responding of NL3 mice during progressive ratio. NL3 (*n* = 5 beside, 5 opposite) and WT (*n* = 5 beside, 5 opposite) mice underwent a lever FR/PR task. (A) In this experiment, mice were trained for a maximum of 34 sessions, with 10 FR1 SL, seven FR1 DL, five FR3, and four FR5 sessions prior to the first PR session. Mice were then individually progressed to up to three 2‐h PR sessions interspersed with FR5 sessions. The final session was a 6‐h PR schedule. Each mouse underwent 6‐h PR only once, though they were spread across mice over 3 days. The operant apparatus was set up in both the standard and inverse configurations, meaning the levers were either beside the reward magazine or opposite the reward magazine, respectively. At progression, there were no genotype or lever location effects on active or inactive lever presses across (B) FR1 SL or (C) FR1 DL. (D) However, during FR3, NL3 mice performed fewer active lever presses during the first but not the subsequent three progression sessions. Mice in both genotypes exhibited more active lever presses when levers were in the standard configuration. (E) Despite this, no genotype effects were observed during subsequent FR5 training. Again, mice in the standard configuration exhibited elevated active lever responding. (F) There were no effects of genotype or lever location on active lever presses during either 2 or 6‐h PR. (G) Similarly, there was no effect of genotype or lever location on inactive lever presses during 2 or 6‐h PR. Dashed vertical lines represent PR sessions that are represented elsewhere in the figure. All graphs are represented as mean ± SEM. ‘*’ denotes a significant genotype effect, ‘$’ denotes a significant session effect, ‘#’ denotes a significant genotype by session interaction effect, and ‘~’ denotes a significant genotype by lever location interaction effect. FR, fixed ratio; HRS, hours; NL3, neuroligin‐3 R451C mouse model; PR, progressive ratio; WT, wild type. ^$$$^ *p* < 0.001, ^#^ *p* < 0.05, ^~^ *p* < 0.05, ^~~~^ *p* < 0.01, n.s. = not significant.

Furthermore, in FR/PR Cohort 5, mice were progressed individually through the FR training stages to mitigate the previously identified degree of habitual responding. Mice that exhibited < 20% variation in active lever presses across four sessions were progressed to the next training stage. Despite this, there were no genotype differences in the number of sessions required at any FR stages (Figure S3). Plotted in Figure 8 are the four sessions immediately prior to progression for each mouse, grouped by genotype and lever location. Data plots ordered by session number are available in Figure S4.

Naïve mice underwent lever FR training prior to being progressed to PR (Figure 8A). In FR1 SL and DL, there were no genotype or lever location effects at the active lever (Figure 8B,C). Similarly, there were no genotype or lever location effects at the inactive lever during FR1 DL (Figure 8B,C). However, in FR3, NL3 mice exhibited reduced active lever responding during the first progression session (Figure 8D; genotype × session interaction, *p* < 0.05; pairwise comparison: Session 1 *p* < 0.05, Session 2 *p* = 0.08, Session 3 *p* = 0.14, Session 4 *p* = 0.16). Both genotypes exhibited fewer active lever presses when the levers were positioned opposite the reward magazine as opposed to beside during FR3 (Figure 8D; lever location effect, *p* < 0.05) and FR5 (Figure 8E; lever location effect, *p* < 0.001). There were, however, no genotype or lever location effects on inactive lever presses in FR3 or FR5 (Figure 8D,E). Similarly, NL3 mice exhibited a comparable amount of active lever responses to WT mice during FR5 (Figure 8E).

Unlike touchscreens, the standard breakpoint in lever PR is defined as the last reward ratio achieved prior to a period of at least 1 h without reward delivery [46]. However, in the 2‐h lever PR from Cohorts 3 and 4, no mice achieved this threshold. As such, in Cohort 5, we ran additional 6‐h PR sessions hoping to attain a standard breakpoint. Despite this, no mice across either genotype or lever location satisfied this criterion. The 6‐h PR results are therefore also depicted as total active and inactive lever presses. Regardless, there were no genotype or lever location effects for active lever presses across both the 2 and 6‐h PR sessions (Figure 8F). Similarly, there were no genotype or lever location effects on inactive lever presses (Figure 8G).

### 3.7 FR/PR Cohort 6: Touchscreen Chambers With Naïve Mice

**FIGURE 9:**
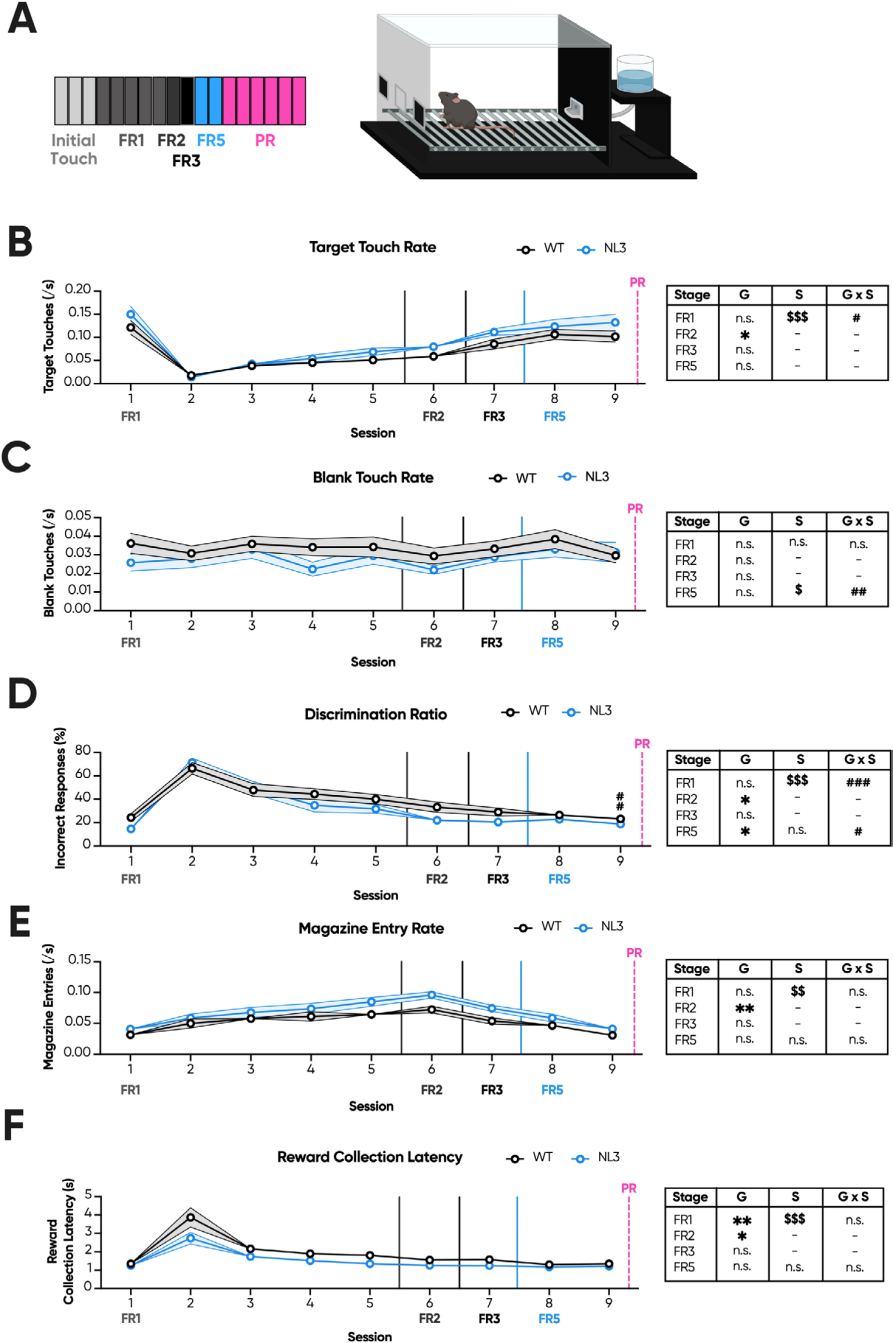
Naïve NL3 mice exhibit similar response patterns to wild‐type mice during touchscreen fixed‐ratio training. NL3 (*n* = 15) and WT (*n* = 12) mice underwent touchscreen FR/PR. (A) This paradigm consisted of 3 pre‐training sessions and 15 training sessions with five FR1, one FR2, one FR3, and two FR5 sessions. Mice were then progressed to six consecutive sessions of PR. (B) NL3 mice exhibited increased target touch rates only during FR2 and (C) comparable blank touch rates to WT mice throughout the entirety of FR training. Accordingly, (D) the discrimination ratio was largely unaltered across genotypes, though NL3 mice were improved in FR2 and one session of FR5. Meanwhile, (E) magazine entry rates were increased in the NL3 mice during FR2, but otherwise indistinguishable from WT mice. (F) Reward collection latencies were decreased in NL3 mice during early FR training, but this was attenuated by late FR training. Solid vertical lines represent the transition from one FR stage to the next. Dashed vertical lines represent PR sessions that are represented elsewhere. All graphs are represented as mean ± SEM. ‘*’ denotes a significant genotype effect, ‘$’ denotes a significant session effect, and ‘#’ denotes a significant genotype by session interaction effect. FR, fixed ratio; G, genotype effect; G × S, genotype by session interaction; NL3, neuroligin‐3 R451C mouse model; PR, progressive ratio; S, session effect; WT, wild type. *p < 0.05, **p < 0.01, ^$^ *p* < 0.05, ^$$^ *p* < 0.01, ^$$$^ *p* < 0.001, ^#^ *p* < 0.05, ^##^ *p* < 0.01, ^###^ *p* < 0.001, n.s. = not significant.

The location of the levers in relation to the site of reward delivery did not impact the PR outcome in Cohort 5, regardless of genotype or session length. Though animal numbers were relatively small due to breeding limitations, goal‐tracking is unlikely to account for the large and consistent reduction in PR responding observed in FR/PR Cohort 1. As such, we returned to the touchscreen chambers with a naïve cohort, hypothesizing that prior experience in a touchscreen task might better explain the reduced responding of Cohort 1. Initial touch training and an extended FR1 were required to compensate for inexperience before mice were progressed through FR2, FR3, and two sessions of FR5 before PR (Figure 9A).

There was a genotype by session interaction effect on target touch rates during FR1 (Figure 9B; genotype × session interaction, *p* < 0.05; pairwise comparison: Session 1 *p* = 0.28, Session 2 *p* = 0.84, Session 3 *p* = 0.68, Session 4 *p* = 0.27, Session 5 *p* = 0.25), though this did not survive multiple comparison correction. Further, during FR2, NL3 mice exhibited significantly greater target touches (Figure 9B; genotype effect, *p* < 0.05), though this was absent during FR3 and FR5 (Figure 9B). Conversely, NL3 mice exhibited no differences in blank touch rates compared to WT mice throughout FR1, FR2, FR3 and FR5 (Figure 9C). These results were contrary to our prior touchscreen results wherein reduced target touch rates and altered blank touch rates were observed throughout training (Figures 1 and 3). NL3 mice exhibited improved discrimination ratios across FR2 (Figure 9D; genotype effect, *p* < 0.05) and one session of FR5 (Figure 9D; genotype × session interaction, *p* < 0.05; pairwise comparison: Session 1 *p* = 0.22, Session 2 *p* < 0.001), with comparable levels to WT mice during FR1 (Figure 9D; genotype × session interaction, *p* < 0.001; pairwise comparison: Session 1 *p* = 0.44, Session 2 *p* = 0.64, Session 3 *p* = 0.19, Session 4 *p* = 0.18, Session 5 *p* = 0.05) and FR3 (Figure 9D). Curiously, NL3 mice exhibited elevated magazine entry rates during the single FR2 session (Figure 9E; genotype effect, *p* < 0.01) but were otherwise indistinguishable from WT rates during FR1, FR3 and FR5 (Figure 9E). Similarly, NL3 mice exhibited reduced collection latencies during FR1 (Figure 9F; genotype effect, *p* < 0.01) and FR2 (Figure 9F; genotype effect, *p* < 0.05), which was not observed during FR3 or FR5 (Figure 9F).

**FIGURE 10:**
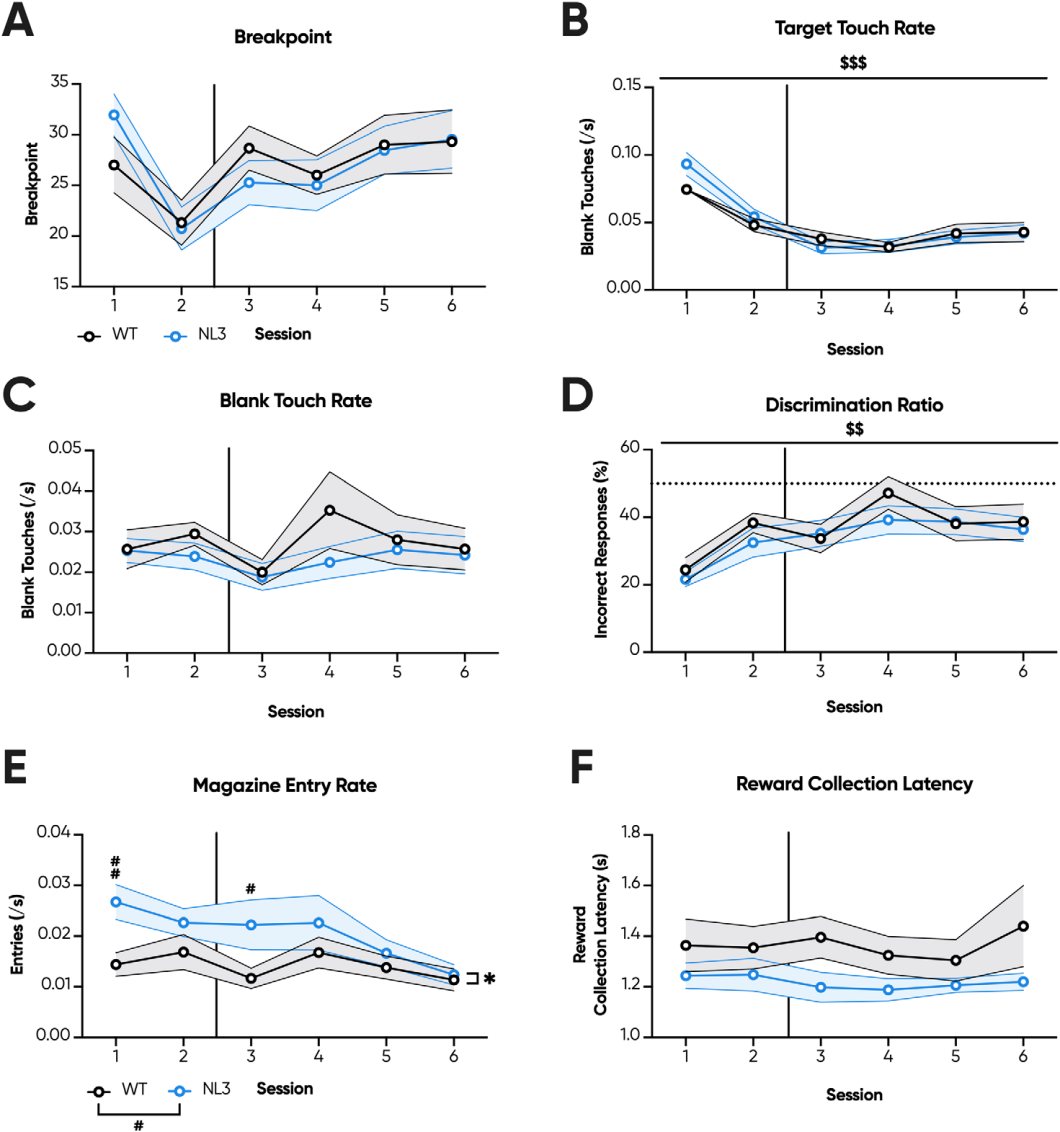
Naïve NL3 mice exhibit similar responding to wild‐type mice during touchscreen progressive ratio. NL3 (*n* = 15) and WT (*n* = 12) mice underwent six consecutive sessions of PR; the first two sessions included a timeout, wherein 5 min of inactivity would result in early termination of the task. In the latter four sessions, however, this timeout was eliminated, and all mice were permitted an hour to perform the task. In this paradigm, there were no genotype effects on (A) breakpoint, (B) target touch rates, (C) blank touch rates, or (D) discrimination ratio. However, NL3 mice again exhibited (E) elevated magazine entry rates, though (F) indistinguishable reward collection latencies. Solid vertical lines represent the transition from PR sessions with a timeout for 5 min of inactivity to PR sessions without an inactivity timeout. All graphs are represented as mean ± SEM. ‘*’ denotes a significant genotype effect, ‘$’ denotes a significant session effect, and ‘#’ denotes a significant genotype by session interaction effect. NL3, neuroligin‐3 R451C mouse model; WT, wild‐type. *p < 0.05, ^$$^ *p* < 0.01, ^$$$^ *p* < 0.001, ^#^ *p* < 0.05, ^##^ *p* < 0.01.

For the first two sessions of PR, the 5‐min timeout previously described was maintained. However, during the latter 4 PR sessions, this timeout was removed. Instead, mice were permitted the full hour to interact with the task, more closely mimicking the lever PR. Herein, no genotype effects were observed across breakpoint (Figure 10A), target touch rates (Figure 10B), blank touch rates (Figure 10C), or discrimination ratio (Figure 10D). As before, NL3 mice exhibited increased magazine entry rates during the first and third PR sessions (Figure 10E; genotype × session interaction, *p* < 0.05; pairwise comparison: Session 1 *p* < 0.01, Session 2 *p* = 0.11, Session 3 *p* < 0.05, Session 4 *p* = 0.24, Session 5 *p* = 0.45, Session 6 *p* = 0.72); though now produced similar reward collection latencies to WT mice throughout PR (Figure 10G). Combined, these results suggest that prior touchscreen training differentially impacts task engagement during FR/PR training between WT and NL3 mice.

### 3.8 Metabolic Chambers

Lastly, we considered the impact of food restriction and hypothesised that metabolism may account for altered reward strength between genotypes. Using automated housing chambers and minispec MRI, we identified a trend toward hyperactivity and a significant reduction in body fat in free‐feeding NL3 mice compared to free‐feeding WT mice. However, these body fat differences were absent in food‐restricted mice, and no other metabolic differences were observed at the genotype level (Figure S5).

## 4 Discussion

We observed no overarching evidence of altered non‐social motivation in the NL3 mouse model of autism. Instead, altered PR responding in two of the six cohorts tested (reduced and elevated, respectively) is more readily explained by an experience‐dependent shift in non‐motivational behaviours. These genotype‐level behavioural differences impacted the shaping and performance of PR and thus the ability of task metrics to resolve motivational state. Our findings raise important considerations for the design of experiments to assess motivation across genotypes, supporting the consideration of the overall training schedule to contextualise behavioural readouts. Our findings, therefore, suggest that the NL3 mouse model best represents autistic individuals with intact non‐social motivation. Reduced motivation for social and non‐social rewards is frequently reported in autism [8], though not ubiquitously [47, 48, 49]. Indeed, the social motivation hypothesis of autism does not necessitate globally impaired motivation. Instead, an imbalance could arise from impaired social yet otherwise comparable non‐social reward processing. As such, alongside known social interaction deficits in NL3 mice [18, 19, 21, 22, 25, 27], motivation for non‐social reward appears unaffected.

Prior training in the rodent‐adapted continuous performance task (rCPT) altered the responses of NL3 mice during high‐effort touchscreen FR/PR. In rCPT, mice are trained to withhold responding in the majority of trials. Mice that respond to unconditioned stimuli enter correction trials, wherein the same stimulus is presented until the subject manages to correctly withhold responding ([50]). Curiously, NL3 mice are better than WT mice at correctly withholding responding during rCPT [20, 24]. This reduction in impulsive responding appears carried forward into FR training in Cohorts 1 and 2, with NL3 mice generally taking longer to complete the full 30 FR trials (Figure S1). When progressed to PR, rCPT‐trained NL3 mice more readily deconditioned to the conditioned stimulus. This reduction in stimulus interaction was overcome by prolonging FR training in the second touchscreen cohort. However, a compensatory increase in overall responsivity to the touchscreen in late FR5 training occurred as a result, as evidenced by elevated blank touch rates that coincided with the amelioration of the target touch rate. Curiously, ATO is known to produce stronger effects in subjects with greater baseline impulsivity [51], and the administration of 3 mg/kg ATO in this cohort produced a greater reduction in breakpoint in NL3 mice than WT mice. MPH, on the other hand, increased breakpoint to similar degrees in both genotypes. This MPH effect is well‐described in rodents but differs from the human response to MPH [34, 36]. Crucially, no differences during FR training and consecutive PR sessions were observed between naïve NL3 and WT mice. Thus, reductions in breakpoint and target touch rate in Cohort 1 likely arose from a combination of prior task training and a short FR/PR training schedule, rather than differences in non‐social motivation.

Secondary touchscreen measures further support the conclusion that NL3 have intact non‐social motivation. Indeed, magazine entry rates were intermittently elevated in NL3 mice. We initially considered whether NL3 exhibit greater goal‐tracking, enticing them away from the response site and thus reducing their target touch rate [45]. However, a subsequent lever experiment wherein the response and reward collection site were either proximal or distant revealed no differences in sign‐ versus goal‐tracking. Intriguingly, magazine entry rates increased in NL3 mice as breakpoint reduced in our first cohort, reaching significant elevation above WT mice during the final PR session. Thus, NL3 mice appear in this cohort interested in the reward but likely interacted less with the stimulus due to reduced certainty that the interaction led to reward delivery. Similarly, reward collection latencies were consistently reduced in rCPT‐trained NL3 mice, though this was not observed in naïve mice. Reduced reward collection latencies may indicate increased motivation for reward [42, 52]. However, considered in context, it is perhaps most likely related to the formation of repetitive motor sequences over time. NL3 mice are indeed known to form enhanced motor sequences upon repeated exposure, as observed during rotarod [53]. This also aligns with the human autism phenotype wherein motor stereotypies are one of the most frequently observed features [54].

Conversely, when FR/PR was performed in lever chambers, NL3 mice revealed a heightened sensitivity to the effects of overtraining. The extended training paradigm in Cohort 3 led to NL3 exhibiting elevated active lever presses compared to WT mice during late FR5 and PR. 3 mg/kg ATO under these conditions produced a moderate drug effect, but genotype remained the most significant factor driving active lever responding. However, in Cohort 4 that underwent shorter lever training, NL3 mice produced a comparable number of active lever responses throughout FR5 and PR. Treatment with both 1 mg/kg and 3 mg/kg ATO in this cohort reduced active lever responses regardless of genotype. Curiously, however, 3 mg/kg ATO did reduce active lever responding less in NL3 mice than WT mice. In the fourth cohort, the ATO drug probes overlapped in terms of session number with the appearance of the strongest overtraining effects in the third cohort. Thus, this reduced sensitivity to 3 mg/kg ATO may be an early indication of the emergence of overtraining effects [51]. Accordingly, active lever responses were steadily increasing in the NL3 mice over the latter sessions of PR, though this did not reach significance across the intentionally limited number of sessions. The lever studies therefore also revealed no overall evidence of motivational dysfunction in NL3 mice. On the contrary, NL3 mice exhibited increased responding in one cohort, though this is likely attributable to a heightened degree of habitual responding following overtraining [55]. Akin to the diminishing return of reward during PR, reversal learning tasks rely upon error feedback from the absence of reward to update behaviour [56]. NL3 mice are indeed slower to adapt during reversal learning [26, 57], persevering with previously trained responses. These elevated habitual phenotypes relate to inflexibility, leading to restricted and repetitive behaviours [55]: the presence of which represents one of the core diagnostic criteria for autism [1].

At last, to investigate basic reward drive without operant conditioning, we utilised a cocaine CPP protocol. In CPP, NL3 mice were hyperactive at baseline, but equally sensitive to the reinforcing effects of cocaine. Interestingly, a trend towards hyperactivity was also observed in the NL3 mice housed within our automatic metabolic chambers and is a phenotype commonly reported in the human autism population [58]. Though our metabolic chambers did not identify broad metabolic dysfunction in free‐feeding or food‐restricted NL3 mice, the impact of the different rewards used between cohorts cannot be ruled out. Milkshake, for example, is known to have greater reward strength for mice than sucrose [59]. Further, sucrose is a simple carbohydrate composed of glucose and fructose, while milkshake is dense in calories and has a complex nutritional profile ([60]). Both factors may interact with the NL3 genotype to affect task performance, particularly given prior evidence of gut dysfunction [61, 62] and atypical fat metabolism reported in our study (Figure S5).

Our findings also generate some general recommendations for the design of FR/PR experiments. First, an individual approach to task progression versus group progression helps mitigate the influence of differential learning curves during task shaping. Second, perhaps unsurprisingly, the relevance of PR results to underlying motivational state is easier to interpret from naïve mice. This is particularly relevant given the enduring popularity of rodent behavioural batteries [63, 64, 65], including in touchscreen testing [41, 66], and test re‐test effects have not been well explored. Prior training, particularly if that training occurs within the same context and relies upon similar interactions and stimuli, is likely to influence subsequent task shaping and performance [67]. Third, testing should be long enough to create stable task associations but varied enough to discourage habit formation [68, 69], especially in the context of a mouse model with an increased tendency for habitual behaviour. Where possible, tasks should be designed to prevent habitual responding from becoming an optimal strategy. Examples of mitigating strategies include the use of timeouts, correction trials, maintenance schedules, and punishments for premature responses.

Our results also question the feasibility of standard breakpoint definitions. No mouse across all three iterations of PR longer than 1‐h achieved standard breakpoint, defined as the last ratio performed prior to a period of 1‐h of inactivity [46]. This is despite extending session length to 6 h in Cohort 5; though this could be related to the high reward strength of milkshake. Further, the standard touchscreen protocol [70] includes an early termination for 5‐min of inactivity and a different definition of breakpoint to standard lever PR [46], affecting our ability to directly compare metrics across cohorts. However, varying experimental design, prior experiences, and contexts can help resolve whether phenotypes are robust and withstand heterogeneity. Our study comes amidst a call for the introduction of systematic heterogenization to animal behaviour research [65]. The basic principle argues that intentional heterogeneity within a study design improves its external validity outside of the controlled lab environment and its reproducibility across lab groups. Finally, we aim to contribute to the growing trend towards open, transparent, and replicable science by freely sharing our datasets and statistical scripts. Moreover, we hope that the transparent presentation and discussion of our behavioural training facilitates interpretation and reproducibility [71, 72].

## Conflicts of Interest

The authors declare no conflicts of interest.

## Supporting information


**Figure S1:** Total schedule lengths across touchscreen FR and PR cohorts.
**Figure S2:** Total distance travelled during the conditioning days of a cocaine‐induced conditioned place preference paradigm.
**Figure S3:** Number of FR and PR sessions performed during Cohort 5.
**Figure S4:** FR and PR lever presses from Cohort 5 ordered by session number.
**Figure S5:** Minimal metabolic phenotype observed in minispec MRI of body composition and automated Promethion housing chambers.

**Table S1:** Summary of statistical results. The GLM/GLMM distribution family for each dataset was selected based on the type and distribution of the dependent variable, as well as the overall experimental design, as detailed in the paper—Methods. In this supplementary table, we provide the experimenter and task stage to permit filtering, as well as the dependent and independent variables used to construct the model. Statistical output includes the statistical model used, the coefficient, lower 2.5% confidence interval (CI), upper 2.5% CI, and *p* value. In statistical summary, * = genotype effect, $ = session effect, # = genotype × session interaction effect, @ = drug effect, % = genotype × drug interaction effect, > = drug × session interaction effect, ^ = genotype × drug × session interaction effect, & = dose effect, + = genotype × dose interaction effect, ~ = lever location effect, { = genotype × lever location interaction effect, < = session × lever location effect, and} = genotype × session × lever location interaction effect. One symbol refers to *p* < 0.05, two to *p* < 0.01, and three to *p* < 0.001. Where significant two‐way interaction effects were observed, post hoc *p* values are reported after correction using Tukey's honest significant difference. Processed datasets and the statistical scripts used to obtain these results are freely available at https://github.com/rikidingwall/pr‐cpp‐gbb‐2025.

## Data Availability

The data that support the findings of this study are openly available in Github at https://github.com/rikidingwall/pr‐cpp‐gbb‐2025.
