# Supplementary material for: Training Schedule Affects Operant Responding Independent of Motivation in the Neuroligin‐3 R451C Mouse Model of Autism
Source: Genes Brain Behav. 2025 Aug 15;24(4):e70032. doi: 10.1111/gbb.70032 (PMC12356647; doi:10.1111/gbb.70032)
Supplement: Supplementary file 1 — Figure S1: Total schedule lengths across touchscreen FR and PR cohorts. Figure S2: Total distance travelled during the conditioning days of a cocaine‐induced conditioned place preference paradigm. Figure S3: Number of FR and PR sessions performed during Cohort 5. Figure S4: FR and PR lever presses from Cohort 5 ordered by session number. Figure S5: Minimal metabolic phenotype observed in minispec MRI of body composition and automated Promethion housing chambers. [file GBB-24-e70032-s001.docx]

Training schedule affects operant responding independent of motivation in the neuroligin-3 R451C mouse model of autism

***Riki Dingwall****^1,2^, Carlos May^1^, Jackson A. McDonald^1^, Thomas Hill^1^, Robyn Brown^1,3^, Andrew J. Lawrence^1^, Anthony J. Hannan^1,2,+^, Emma L. Burrows^1,2^*

+ Corresponding author: Anthony J. Hannan [anthony.hannan@florey.edu.au](mailto:anthony.hannan@florey.edu.au)

**Affiliations**

1 The Florey Institute of Neuroscience and Mental Health, University of Melbourne, Parkville, Melbourne, Australia

2 Faculty of Medicine, Dentistry & Health Sciences, University of Melbourne, Parkville, VIC, Australia

3 Department of Biochemistry & Pharmacology, University of Melbourne, Parkville, VIC, Australia

# **Supplementary Figures**


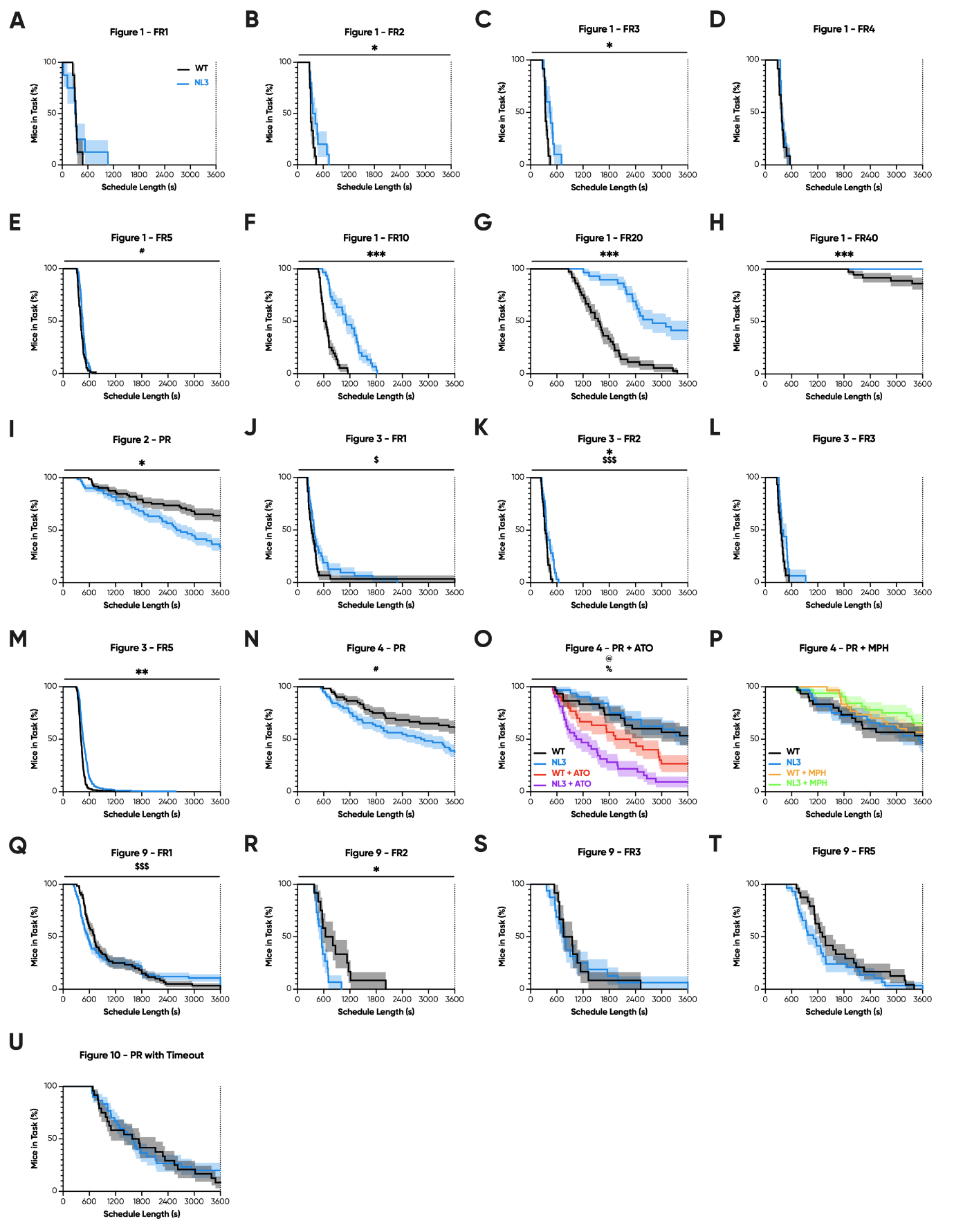


**Supplementary Figure 1. Total schedule lengths across touchscreen FR and PR cohorts**

Unlike the operant FR/PR where schedule length was fixed, touchscreen FR/PR involved conditions for early termination of the task schedule. Early termination occurred upon (i) achieving a maximum number of rewards in FR and (ii) a 5-minute period of inactivity in the presence of the conditioned stimulus in PR. WT and NL3 mice met these conditions inconsistently across the touchscreen experiments. Accordingly, total schedule length was variegated, rendering total target and blank touches difficult to interpret. Both touch types were therefore represented in the body of the paper as rates. Data from cohort one was presented in Figures 1 and 2, here spanning A-I (WT n=12; NL3 n=10). In this cohort, schedule length for NL3 mice was primary longer than their WT counterparts (**B-C, E-H**), although it was at times indistinguishable (**A, D**). During PR, NL3 mice exhibited shorter schedule lengths compared to WT mice due to the addition of the early timeout for non-response (**I**). Data from cohort two was presented in Figures 3 and 4, here spanning J-P (WT n=15; NL3 n=16). This cohort additionally underwent PR drug probes using ATO and MPH. In this cohort, NL3 mice again exhibited a mixture of longer schedule lengths (**K, M**) and indistinguishable schedule lengths (**J, L**) compared to WT mice. During PR, NL3 mice were again more likely to trigger the timeout (**N**). Intraperitoneal administration of 3 mg/kg ATO reduced schedule lengths during PR in both genotypes but to a greater degree in NL3 mice (**O**). Meanwhile, 3 mg/kg MPH administration did not significant alter schedule length in either genotype (**P**). Lastly, data from cohort six was presented in Figures 9 and 10, here spanning Q-U (WT n=12; NL3 n=15). Unlike the previous touchscreen cohorts with prior rCPT training, the naive NL3 mice in this cohort exhibited largely unaltered FR schedule lengths compared to their WT counterparts (**Q, S-T**). Indeed, during FR2, NL3 mice were faster than WT mice to achieve maximum rewards (**R**). Mice in this cohort underwent six consecutive PR sessions. However, the timeout condition was removed for the latter four and the schedule length was instead fixed at 3,600 seconds. Only schedule length data from the first two PR sessions has therefore been plotted and analysed. In a manner dissimilar once again with the prior touchscreen cohorts, PR schedule length did not differ in this cohort according to genotype (**U**). All graphs are represented as mean ± SEM. Statistical analysis was performed using Cox proportional hazards models in R. “✱” denotes a significant genotype effect, “**$**” denotes a significant session effect, “**#**” denotes a significant genotype by session interaction effect, “@” denotes a significant drug effect, and “%” denotes a significant genotype by drug interaction effect. WT = wildtype, NL3 = neuroligin-3 R451C mouse model, FR = fixed ratio, PR = progressive ratio, ATO = atomoxetine, MPH = methylphenidate, ✱ = P<0.05, ✱✱ = P<0.01, ✱✱✱ = P<0.001, **$** = P<0.05, **$$** = P<0.01, **$$$** = P<0.001, **#** = P<0.05, **##** = P<0.01, **###** = P<0.001, **@** = P<0.05, **@@ =** P<0.01, **@@@** = P<0.001, **%** = P<0.05, **%%** = P<0.01, **%%%** = P<0.001


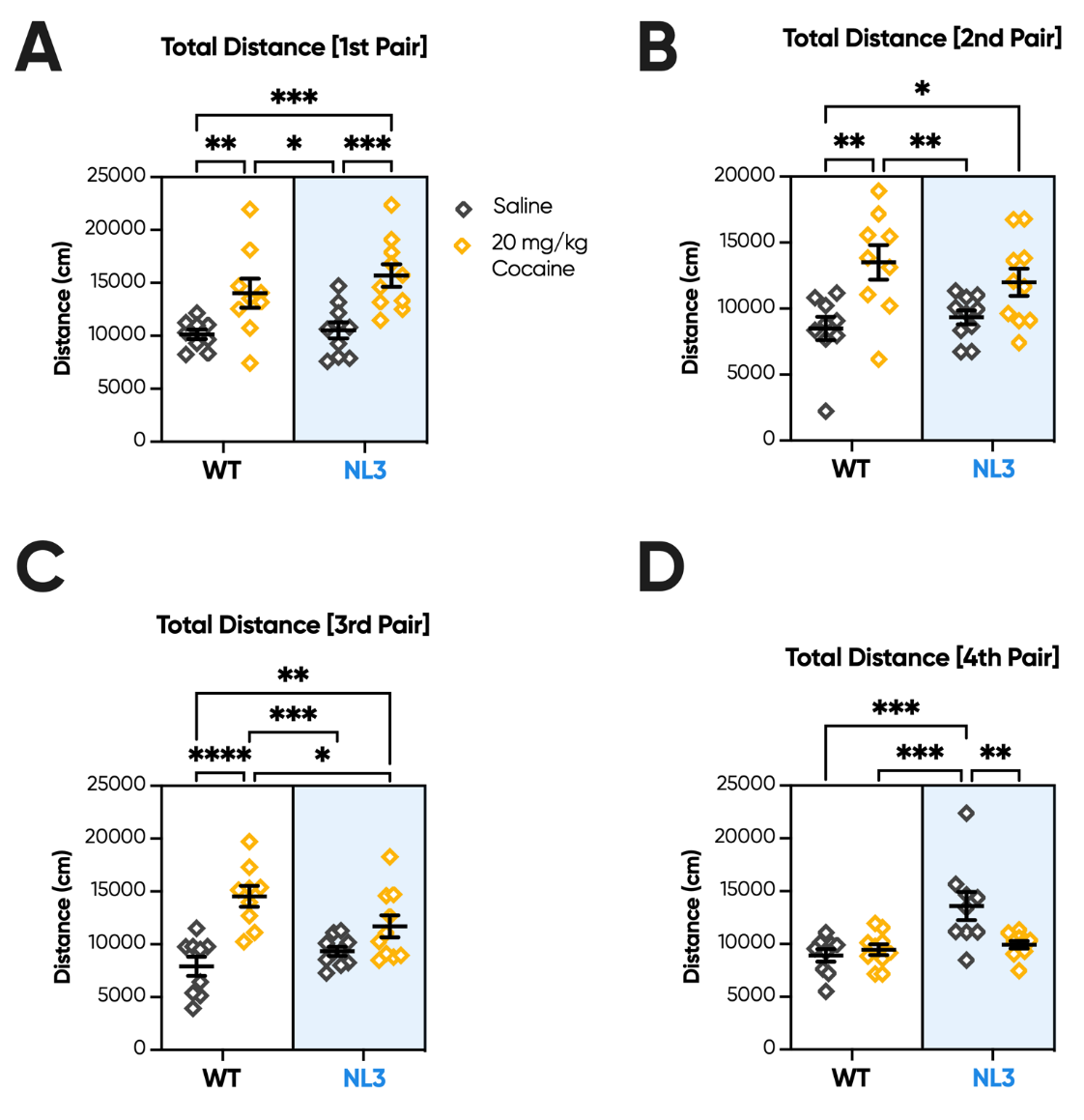


**Supplementary Figure 2. Total distance travelled during the conditioning days of a cocaine-induced conditioned place preference paradigm**

WT (n=9) and NL3 (n=10) mice underwent a conditioned place preference paradigm using 20 mg/kg cocaine. As depicted in Figure 7, conditioning took place in paired two-day blocks. Data from these conditioning pairs are plotted and analysed above. During conditioning, mice are restricted to their assigned conditioned chamber when receiving cocaine or their assigned unconditioned chamber when receiving saline. In the first conditioning pair, cocaine induced hyperactivity in both genotypes (**A**). However, NL3 mice began habituating to the locomotor effects of cocaine by the second conditioning days (**B**) and were indistinguishable from saline-treated NL3 mice by the third conditioning pair (**C**). During conditioning pair 4, both genotypes had habituated to cocaine-induced hyperactivity (**D**). However, saline-treated NL3 mice curiously exhibited hyperactivity compared to all other conditions and indeed compared to saline-treated *NL3* mice in other conditioning pairs. Two possible interpretations include heightened reward-seeking or heightened frustrative locomotor activity following reward omission. All graphs are represented as mean ± SEM. Statistical analysis was performed using generalized linear models in R. “✱” denotes a significant group effect. WT = wildtype, NL3 = neuroligin-3 R451C mouse model, ✱ = P<0.05, ✱✱ = P<0.01, ✱✱✱ = P<0.001


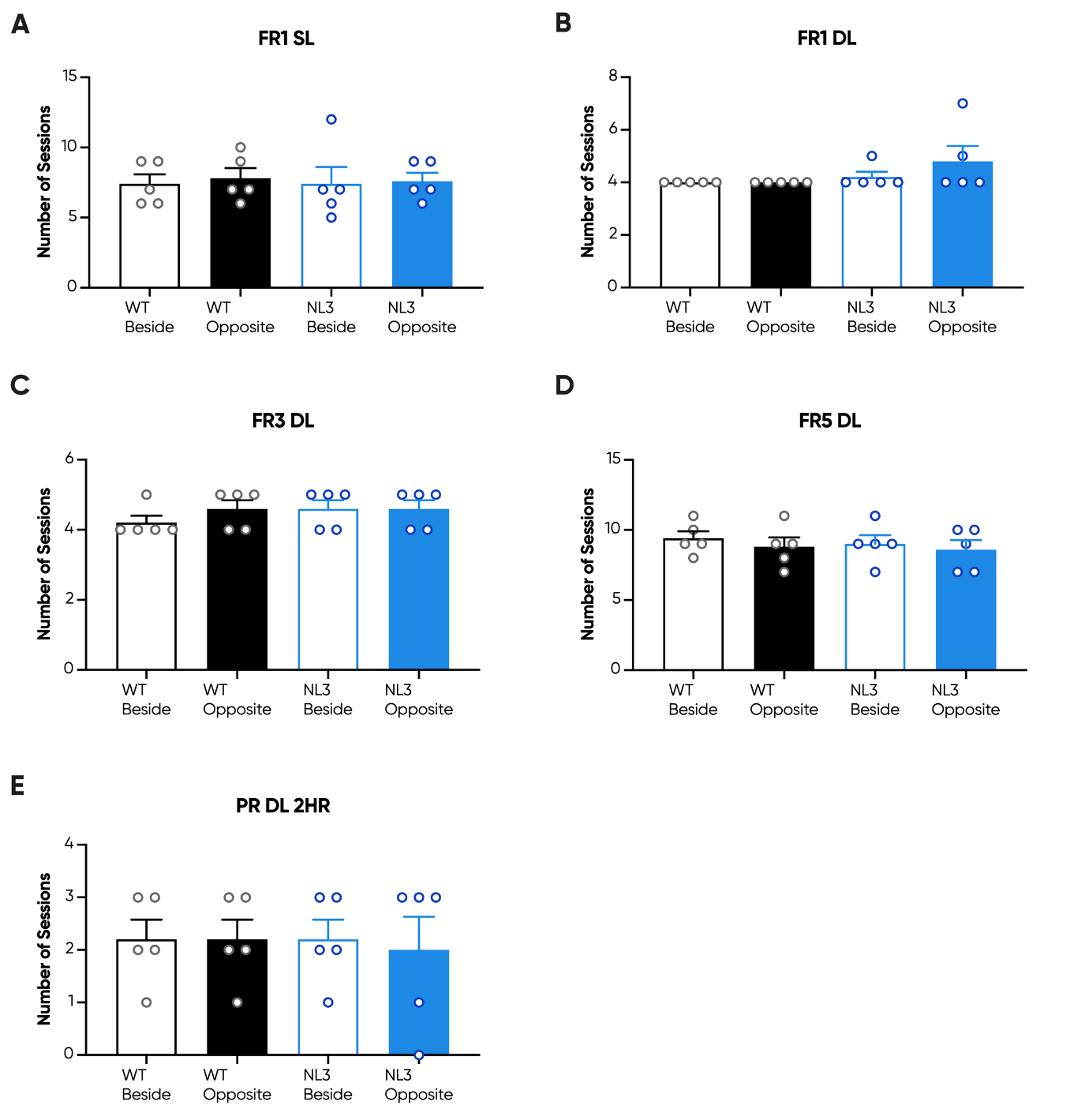


**Supplementary Figure 3. Number of FR and PR sessions performed during cohort five**

In cohort five, mice were individually progressed through fixed ratio stages once their active lever responding was stable (<20% variation) over four consecutive sessions. However, there were no genotype or lever location differences in the number sessions required to successfully progress during FR, including FR1 (**A-B**), FR3 (**C**) and FR5 (**D**). A total of three 2-hour PR sessions were performed, but mice were only included if they satisfied progression from FR5. No genotype or lever location effects were however observed in the number of 2-hour PR sessions (**E**). All graphs are represented as mean ± SEM. Statistical analysis was performed using generalized linear models in R. WT = wildtype, NL3 = neuroligin-3 R451C mouse model, FR = fixed ratio, SL = single lever, DL = double lever, PR = progressive ratio


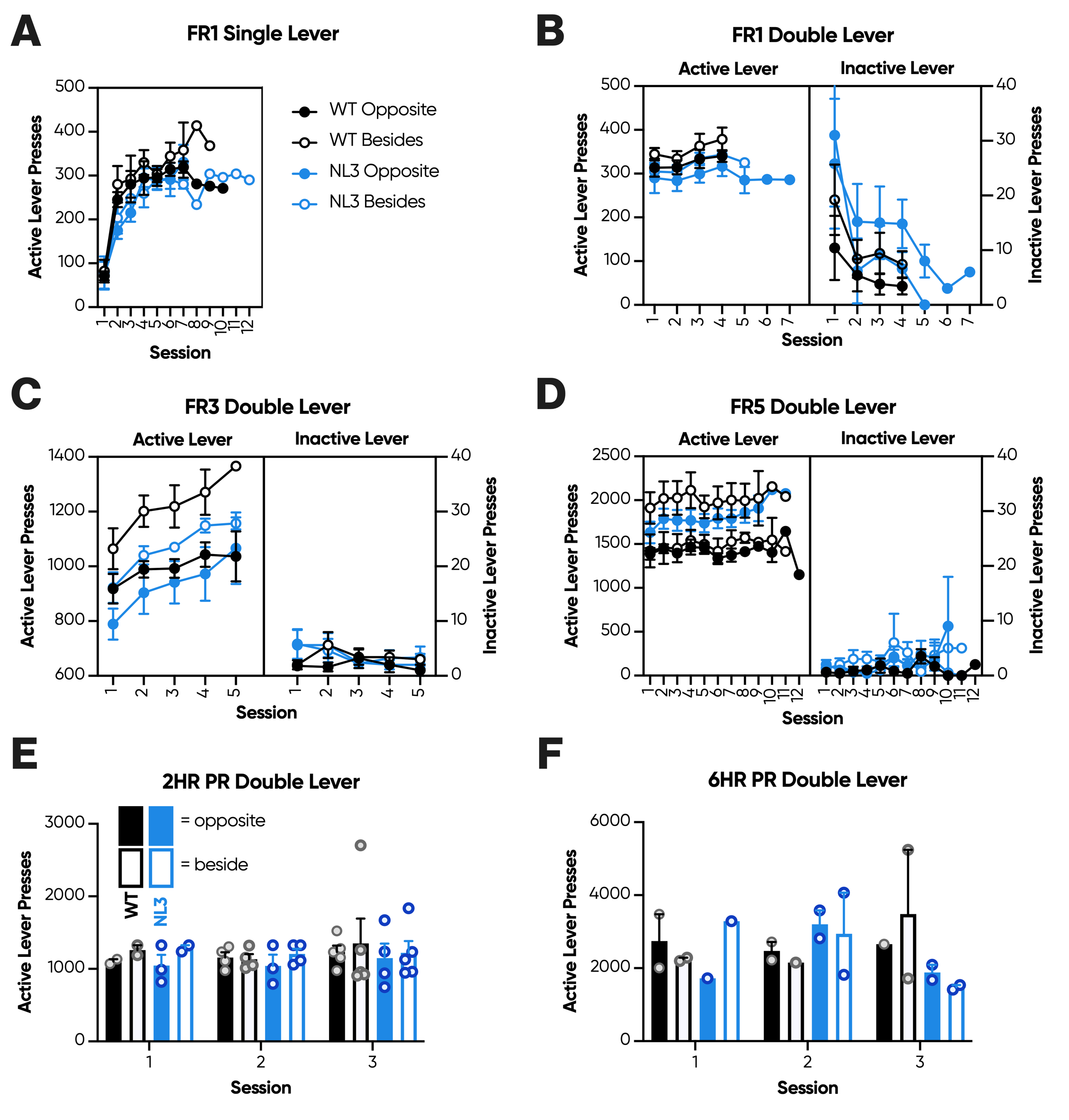


**Supplementary Figure 4. FR and PR lever presses from cohort five ordered by session number**

As aforementioned, mice in cohort 5 were individually progressed through fixed ratio stages once their active lever responding was stable (<20% variation) over four consecutive sessions. Figure 8 plotted and analysed the four sessions that led to progression at each FR stage. Despite finding no genotype or lever location differences in the number of trials received at any FR stage, presses by session number are represented here for transparency. As individual mice received differing amounts of FR sessions, each symbol on the XY plot does not represent the average of a consistent number of animals. Mice across both genotypes were randomly assigned to operant chambers with levers either opposite or beside the reward tray (**A**). Active and inactive lever data is represented by session number for FR1 SL (**B**), FR1 DL (**C**), FR3 (**D**), and FR5 (**E**). In Figure 8, the three 2-hour PR sessions were collapsed prior to analysis, following the absence of a session effect. In (**F**), active lever presses during 2-hour PR are plotted separately by session. Lastly, all mice underwent a single 6-hour PR session. This occurred over three days due to experimental constraints. Data from these sessions was collapsed before being plotted and analysed in Figure 8. Here, (**G**) depicts active lever presses during 6-hour PR separated by session date. No mouse was retested. Thus, each dot represents a novel mouse undergoing 6-hour PR. All graphs are represented as mean ± SEM. No formal statistical analysis was performed on the data as presented. WT = wildtype, NL3 = neuroligin-3 R451C mouse model, FR = fixed ratio, SL = single lever, DL = double lever, HR = hour, PR = progressive ratio


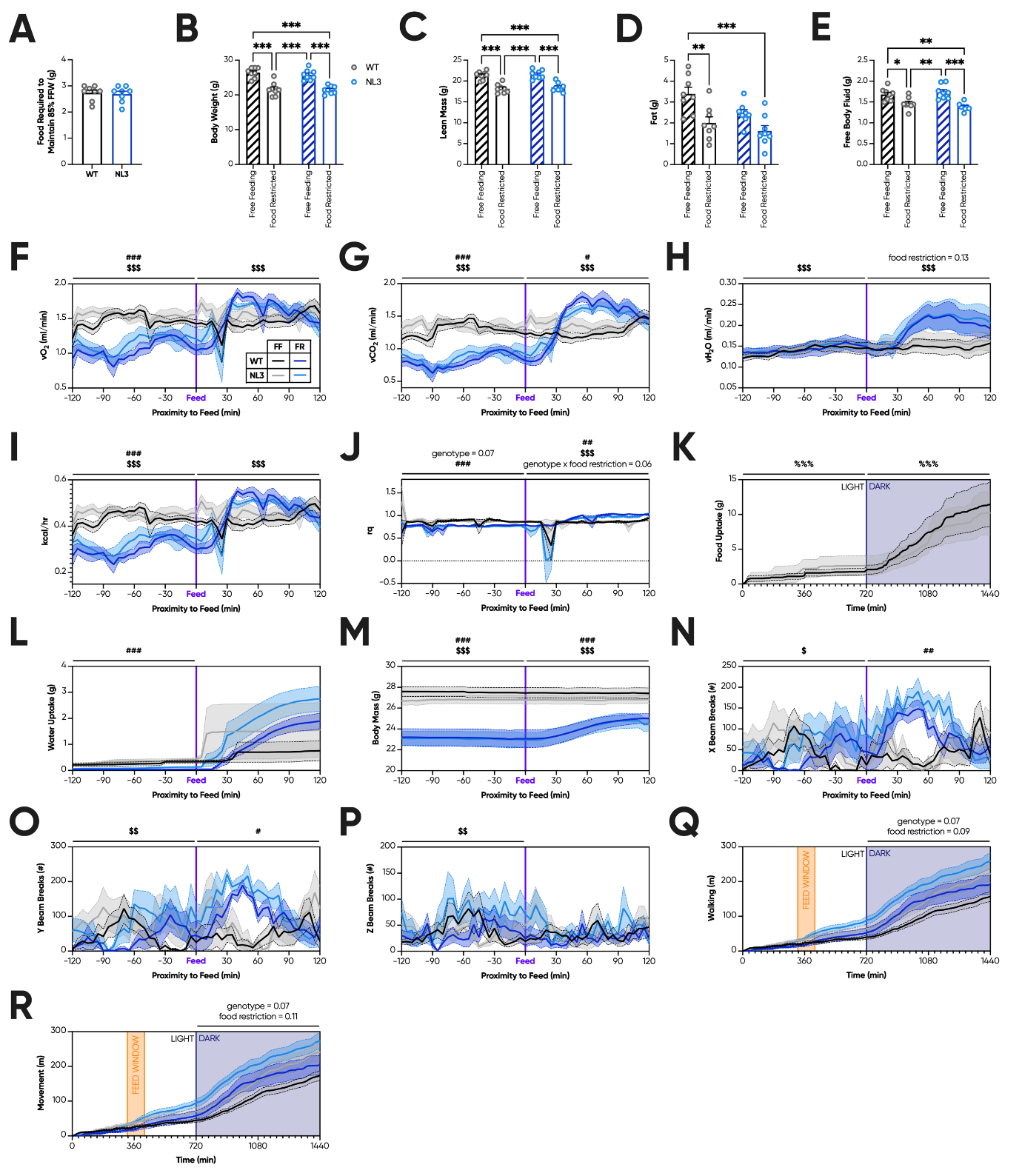


**Supplementary Figure 5. Minimal metabolic phenotype observed in minispec MRI of body composition and automated Promethion housing chambers**

At 8 weeks of age, single-housed NL3 and WT male mice underwent food-restriction to 85% of their free-feeding body weight (NL3-FR; WT-FR). Littermate controls were provided unrestricted access to chow ad libitum (NL3-FF; WT-FF). At 11 weeks of age, all mice were mechanically restrained to undergo minispec MRI for body composition analysis. The following day, mice were rehoused from their standard individually ventilated housing to the automated Promethion chambers for metabolic analysis. Mice undergoing food restriction were maintained on a restricted feeding schedule and provided with pre-weighed chow placed into the housing chamber once per day. The internal food hooper was left empty. Alternatively, free feeding mice continued to receive ad libitum access to chow inside the Promethion chamber via the internal food hopper. Mice initially explore the novel housing environment, and this interferes with measurements of baseline metabolism. As such, the first 12 hours was designated habituation and removed from the analysis. Mice are also nocturnal and therefore exhibit different movement and sleep patterns during the 12-hour light (6:30am-6:30pm) and 12-hour dark (6:30pm-6:30am) periods experienced by laboratory mice. As such, a full 24-hour period was analysed starting at the first light period following a >12-hour habituation. This procedure was performed across independent runs in two age-matched cohorts (WT-FF n=8, WT-FR n=8, NL3-FF n=8, NL3-FR n=8). These cohorts have been collapsed for the purposes of data visualisation and analysis. A Promethian chamber faulted during the second run, removing one NL3-FR mouse from the study. Additionally, some automated chamber elements were faulty. Following an iterative review of individual chambers, data arising from faulty readings were removed prior to analysis. For rigor, animal numbers for each panel will be indicated in the legend. Some metabolic outputs were largely stable in free-feeding mice over a 24-hour period, with changes arising mostly in food-restricted mice upon food delivery. For these metrics, data from two hours before feeding and two hours after feeding were plotted and analysed. Other metrics were more heavily influenced by time of day. These metrics were instead plotted and analysed across the entire 24-hour test period, with light and dark periods analysed separately. Food-restricted NL3 and WT mice required similar amounts of food to maintain 85% free-feeding body weight (**A**). Body weight (**B**), lean mass (**C**) and free body fluid (**D**) were all reduced by food restriction, though identically across genotypes. Curiously, despite no overall body weight changes, free-feeding NL3 mice had reduced body fat compared to their WT counterparts. In WT mice, food restriction significantly reduced body fat, while total body fat was indistinguishable from between free-feeding NL3 and both food-restricted genotypes (**E**). Food restriction effects were observed across vO_2_ (**F**; WT-FF n=8, WT-FR n=8, NL3-FF n=8, NL3-FR n=7), vCO_2_ (**G**; WT-FF n=8, WT-FR n=8, NL3-FF n=8, NL3-FR n=7), kcal/hr (**F**; WT-FF n=8, WT-FR n=8, NL3-FF n=8, NL3-FR n=7), rq (**G**; WT-FF n=8, WT-FR n=8, NL3-FF n=8, NL3-FR n=7), water uptake (**L**; WT-FF n=7, WT-FR n=8, NL3-FF n=5, NL3-FR n=7), body mass (**M**; WT-FF n=7, WT-FR n=8, NL3-FF n=5, NL3-FR n=7), X beam breaks (**N**; WT-FF n=8, WT-FR n=8, NL3-FF n=7, NL3-FR n=7), and Y beam breaks (**O**; WT-FF n=8, WT-FR n=8, NL3-FF n=8, NL3-FR n=7). No such food restriction effects were observed in vH_2_O (**H**; WT-FF n=8, WT-FR n=8, NL3-FF n=8, NL3-FR n=7), Z beam breaks (**P**; WT-FF n=8, WT-FR n=8, NL3-FF n=8, NL3-FR n=7), walking (**Q**; WT-FF n=8, WT-FR n=8, NL3-FF n=8, NL3-FR n=7) or total movement (**M**; WT-FF n=8, WT-FR n=8, NL3-FF n=8, NL3-FR n=7). Walking is defined here as movement within the beam system at or faster than 1cm/s. Crucially, no significant genotype effects were observed across any of the measures. However, we observed a genotype-level trend towards increased walking (**Q**) and total movement (**R**) in NL3 mice compared to WT mice, congruent with the hyperactivity observed during CPP. There was also a trend towards altered rq in NL3 mice. Respiratory quotient or rq is a ratio between carbon dioxide production and oxygen consumption. It provides an estimation of the primary macromolecule being metabolised with solely fats producing a rq of ~0.7, proteins ~0.8 and carbohydrates ~1. This trend towards a rq shift in NL3 mice regardless of food access aligns with their reduced fat composition despite similar total food intakes. There was also a tentative trend towards an increase in walking and movement that was driven by food restriction, which may be indicative of unsated food-seeking behaviours. Lastly, total food uptake by free-feeding mice over the 24-hour period was similar across genotypes (**K**; WT-FF n=8, NL3-FF n=7). However, NL3 mice consumed marginally more during the light phase and less during the dark phase than their WT counterparts. All graphs are represented as mean ± SEM. Statistical analysis of body composition data was performed using two-way analysis of variance with Tukey’s correction for multiple comparisons in Prism 10. Meanwhile, the metabolic chamber data was analysed using generalized linear mixed models in R. “✱” denotes a significant genotype effect, “**$**” denotes a significant time effect, “**#**” denotes a significant food restriction effect, and “%” denotes a significant genotype by time interaction effect. WT = wildtype, NL3 = neuroligin-3 R451C mouse model, ✱ = P<0.05, ✱✱ = P<0.01, ✱✱✱ = P<0.001, **$** = P<0.05, **$$** = P<0.01, **$$$** = P<0.001, **#** = P<0.05, **##** = P<0.01, **###** = P<0.001, **%** = P<0.05, **%%** = P<0.01, **%%%** = P<0.001
