# Supplementary material for: Training Schedule Affects Operant Responding Independent of Motivation in the Neuroligin‐3 R451C Mouse Model of Autism
Source: Genes Brain Behav. 2025 Aug 15;24(4):e70032. doi: 10.1111/gbb.70032 (PMC12356647; doi:10.1111/gbb.70032)
Supplement: Supplementary file 2 — Table S1: Summary of statistical results. The GLM/GLMM distribution family for each dataset was selected based on the type and distribution of the dependent variable, as well as the overall experimental design, as detailed in the paper—Methods. In this supplementary table, we provide the experimenter and task stage to permit filtering, as well as the dependent and independent variables used to construct the model. Statistical output includes the statistical model used, the coefficient, lower 2.5% confidence interval (CI), upper 2.5% CI, and p value. In statistical summary, * = genotype effect, $ = session effect, # = genotype × session interaction effect, @ = drug effect, % = genotype × drug interaction effect, > = drug × session interaction effect, ^ = genotype × drug × session interaction effect, & = dose effect, + = genotype × dose interaction effect, ~ = lever location effect, { = genotype × lever location interaction effect, < = session × lever location effect, and} = genotype × session × lever location interaction effect. One symbol refers to p < 0.05, two to p < 0.01, and three to p < 0.001. Where significant two‐way interaction effects were observed, post hoc p values are reported after correction using Tukey's honest significant difference. Processed datasets and the statistical scripts used to obtain these results are freely available at https://github.com/rikidingwall/pr‐cpp‐gbb‐2025. [file GBB-24-e70032-s002.docx]

Training schedule affects operant responding independent of motivation in the neuroligin-3 R451C mouse model of autism

***Riki Dingwall****^1,2^, Carlos May^1^, Jackson A. McDonald^1^, Thomas Hill^1^, Robyn Brown^1,3^, Andrew J. Lawrence^1^, Anthony J. Hannan^1,2,+^, Emma L. Burrows^1,2^*

+ Corresponding author: Anthony J. Hannan [anthony.hannan@florey.edu.au](mailto:anthony.hannan@florey.edu.au)

**Affiliations**

1 The Florey Institute of Neuroscience and Mental Health, University of Melbourne, Parkville, Melbourne, Australia

2 Faculty of Medicine, Dentistry & Health Sciences, University of Melbourne, Parkville, VIC, Australia

3 Department of Biochemistry & Pharmacology, University of Melbourne, Parkville, VIC, Australia

# **Supplementary Tables**

**Figures 1 and 2.**

| **Experimenter** | **Stage** | **Dependent Variable** | **Independent Variable** | **Stats Test** | **Coefficient** | **Lower 2.5% CI** | **Upper 97.5% CI** | **P-Value** | **Stats Summary** | **Pairwise Notes** |
| --- | --- | --- | --- | --- | --- | --- | --- | --- | --- | --- |
| Carlos | PR | Blank Touch Rate | Genotype | GLMM with beta regression | -0.05487 | -0.3562812 | 0.2465352 | 0.721 | n.s. |  |
| Carlos | PR | Blank Touch Rate | Session | GLMM with beta regression | 0.02621 | -0.07600339 | 0.1284231 | 0.615 | n.s. |  |
| Carlos | PR | Blank Touch Rate | Genotype x Session | GLMM with beta regression | 0.03853 | -0.11956517 | 0.196625 | 0.633 | n.s. |  |
| Carlos | FR1 | Blank Touch Rate | Genotype | GLM with zero-inflated beta regression | 0.4001 | -0.6257195 | 1.425928 | 0.445 | n.s. |  |
| Carlos | FR2 | Blank Touch Rate | Genotype | GLM with zero-inflated beta regression | -0.2964 | -0.9354978 | 0.3426217 | 0.363 | n.s. |  |
| Carlos | FR3 | Blank Touch Rate | Genotype | GLM with zero-inflated beta regression | 0.004195 | -0.6789194 | 0.6873096 | 0.99 | n.s. |  |
| Carlos | FR4 | Blank Touch Rate | Genotype | GLM with zero-inflated beta regression | 0.155 | -0.56218 | 0.8721733 | 0.672 | n.s. |  |
| Carlos | FR5 ALL | Blank Touch Rate | Genotype | GLMM with zero-inflated beta regression | 0.41003 | -0.1325941 | 0.9526555 | 0.139 | n.s. |  |
| Carlos | FR5 ALL | Blank Touch Rate | Session | GLMM with zero-inflated beta regression | 0.1624 | -0.01999881 | 0.3448045 | 0.081 | n.s. |  |
| Carlos | FR5 ALL | Blank Touch Rate | Genotype x Session | GLMM with zero-inflated beta regression | -0.1236 | -0.35910369 | 0.1119124 | 0.304 | n.s. |  |
| Carlos | FR5 BEFORE PR | Blank Touch Rate | Genotype | GLMM with zero-inflated beta regression | 0.5831 | -0.07797855 | 1.24407867 | 0.0839 | n.s. |  |
| Carlos | FR5 BEFORE PR | Blank Touch Rate | Session | GLMM with zero-inflated beta regression | -0.226 | -0.47322704 | 0.02130597 | 0.0733 | n.s. |  |
| Carlos | FR5 BEFORE PR | Blank Touch Rate | Genotype x Session | GLMM with zero-inflated beta regression | 0.1349 | -0.16207101 | 0.43195106 | 0.3732 | n.s. |  |
| Carlos | FR5 AFTER PR | Blank Touch Rate | Genotype | GLMM with zero-inflated beta regression | 0.1906 | -0.4438403 | 0.8249529 | 0.556 | n.s. |  |
| Carlos | FR5 AFTER PR | Blank Touch Rate | Session | GLMM with zero-inflated beta regression | -0.2093 | -0.5375549 | 0.1189679 | 0.211 | n.s. |  |
| Carlos | FR5 AFTER PR | Blank Touch Rate | Genotype x Session | GLMM with zero-inflated beta regression | 0.1044 | -0.3152038 | 0.5239543 | 0.626 | n.s. |  |
| Carlos | FR10 | Blank Touch Rate | Genotype | GLMM with zero-inflated beta regression | 0.3958 | -0.22635883 | 1.0179843 | 0.21244 | n.s. |  |
| Carlos | FR10 | Blank Touch Rate | Session | GLMM with zero-inflated beta regression | -0.3918 | -0.67306184 | -0.1105191 | 0.00633 | $$ |  |
| Carlos | FR10 | Blank Touch Rate | Genotype x Session | GLMM with zero-inflated beta regression | 0.396 | 0.04401725 | 0.7478889 | 0.02745 | # | **WT - KI Session 1**  P = 0.9134  **WT - KI Session 2** P = 0.8494  **WT - KI Session 3** P = 0.0078 ** |
| Carlos | FR20 | Blank Touch Rate | Genotype | GLMM with zero-inflated beta regression | 0.98642 | 0.37110723 | 1.60174045 | 0.00168 | ✱✱ |  |
| Carlos | FR20 | Blank Touch Rate | Session | GLMM with zero-inflated beta regression | -0.23129 | -0.42203745 | -0.04054481 | 0.01747 | $ |  |
| Carlos | FR20 | Blank Touch Rate | Genotype x Session | GLMM with zero-inflated beta regression | 0.1787 | -0.06411198 | 0.42151836 | 0.14917 | n.s. |  |
| Carlos | FR40 | Blank Touch Rate | Genotype | GLMM with zero-inflated beta regression | 0.85589 | 0.208566 | 1.5032167 | 0.00956 | ✱✱ |  |
| Carlos | FR40 | Blank Touch Rate | Session | GLMM with zero-inflated beta regression | 0.05462 | -0.1038795 | 0.2131135 | 0.49943 | n.s. |  |
| Carlos | FR40 | Blank Touch Rate | Genotype x Session | GLMM with zero-inflated beta regression | 0.01224 | -0.1896077 | 0.214088 | 0.90539 | n.s. |  |
| Carlos | PR | Blank Touches | Genotype | GLMM with negative binomial regression | 4.22105 | -0.6249113 | 0.1072069 | 0.166 | n.s. |  |
| Carlos | PR | Blank Touches | Session | GLMM with negative binomial regression | -0.25885 | -0.1115491 | 0.2282276 | 0.501 | n.s. |  |
| Carlos | PR | Blank Touches | Genotype x Session | GLMM with negative binomial regression | 0.05834 | -0.2711275 | 0.2086196 | 0.798 | n.s. |  |
| Carlos | FR1 | Blank Touches | Genotype | GLM with negative binomial regression | 0.6466 | -1.414858 | 2.708113 | 0.539 | n.s. |  |
| Carlos | FR2 | Blank Touches | Genotype | GLM with poisson regression | 0.2776 | -0.1053485 | 0.660612 | 0.155 | n.s. |  |
| Carlos | FR3 | Blank Touches | Genotype | GLM with negative binomial regression | 0.1335 | -0.89778 | 1.164843 | 0.79967 | n.s. |  |
| Carlos | FR4 | Blank Touches | Genotype | GLM with negative binomial regression | 0.2693 | -0.8233601 | 1.362026 | 0.629 | n.s. |  |
| Carlos | FR5 ALL | Blank Touches | Genotype | GLMM with negative binomial regression | 1.2147 | 0.30637863 | 2.1229521 | 0.00876 | ✱✱ |  |
| Carlos | FR5 ALL | Blank Touches | Session | GLMM with negative binomial regression | 0.1924 | -0.03390228 | 0.4186463 | 0.09565 | n.s. |  |
| Carlos | FR5 ALL | Blank Touches | Genotype x Session | GLMM with negative binomial regression | -0.1734 | -0.48148344 | 0.1346703 | 0.26994 | n.s. |  |
| Carlos | FR5 BEFORE PR | Blank Touches | Genotype | GLMM with negative binomial regression | 1.3993 | 0.44606441 | 2.3525042 | 0.00401 | ✱✱ |  |
| Carlos | FR5 BEFORE PR | Blank Touches | Session | GLMM with negative binomial regression | -0.4021 | -0.6542718 | -0.1499483 | 0.00178 | $$ |  |
| Carlos | FR5 BEFORE PR | Blank Touches | Genotype x Session | GLMM with negative binomial regression | 0.2537 | -0.07417766 | 0.5815124 | 0.12939 | n.s. |  |
| Carlos | FR5 AFTER PR | Blank Touches | Genotype | GLMM with negative binomial regression | 1.1178 | 0.03230611 | 2.20324049 | 0.0436 | ✱ |  |
| Carlos | FR5 AFTER PR | Blank Touches | Session | GLMM with negative binomial regression | -0.3358 | -0.62129229 | -0.05020894 | 0.0212 | $ |  |
| Carlos | FR5 AFTER PR | Blank Touches | Genotype x Session | GLMM with negative binomial regression | 0.1958 | -0.18686859 | 0.57845114 | 0.3159 | n.s. |  |
| Carlos | FR10 | Blank Touches | Genotype | GLMM with negative binomial regression | 2.199 | 0.97807931 | 3.41990474 | 0.000415 | ✱✱✱ |  |
| Carlos | FR10 | Blank Touches | Session | GLMM with negative binomial regression | -0.2977 | -0.6419098 | 0.04649093 | 0.090031 | n.s. |  |
| Carlos | FR10 | Blank Touches | Genotype x Session | GLMM with negative binomial regression | 0.148 | -0.30293519 | 0.59895495 | 0.520028 | n.s. |  |
| Carlos | FR20 | Blank Touches | Genotype | GLMM with negative binomial regression | 1.7515 | 1.0263824 | 2.4765844 | 0.000002 | ✱✱✱ |  |
| Carlos | FR20 | Blank Touches | Session | GLMM with negative binomial regression | -0.4839 | -0.7813111 | -0.1863949 | 0.00143 | $$ |  |
| Carlos | FR20 | Blank Touches | Genotype x Session | GLMM with negative binomial regression | 0.2517 | -0.1598432 | 0.6632927 | 0.23062 | n.s. |  |
| Carlos | FR40 | Blank Touches | Genotype | GLMM with negative binomial regression | 1.17478 | 0.18038699 | 2.1691683 | 0.0206 | ✱ |  |
| Carlos | FR40 | Blank Touches | Session | GLMM with negative binomial regression | 0.1011 | -0.08250492 | 0.2846971 | 0.2805 | n.s. |  |
| Carlos | FR40 | Blank Touches | Genotype x Session | GLMM with negative binomial regression | 0.01032 | -0.2609837 | 0.2816265 | 0.9406 | n.s. |  |
| Carlos | PR | Breakpoint | Genotype | GLMM with negative binomial regression | -0.38287 | -0.5690114 | -0.1967208 | 0.00006 | ✱✱✱ |  |
| Carlos | PR | Breakpoint | Session | GLMM with negative binomial regression | -0.07146 | -0.1181605 | -0.02475798 | 0.00271 | $$ |  |
| Carlos | PR | Breakpoint | Genotype x Session | GLMM with negative binomial regression | -0.08936 | -0.1627528 | -0.01595929 | 0.01703 | # | **WT - KI Session 1** P=0.2561  **WT - KI Session 2** P=0.0007 (***)  **WT - KI Session 3** P=0.0007 (***)  **WT-KI Session 4**  P=0.0002 (***)   **WT-KI Session 5** P=0.0002 (***)  **WT-KI Session 6** P=0.0007 (***) |
| Carlos | PR | Discrimination Ratio | Genotype | GLMM with beta regression | 0.37047 | 0.03394505 | 0.7069966 | 0.03095 | ✱ |  |
| Carlos | PR | Discrimination Ratio | Session | GLMM with beta regression | 0.1799 | 0.05756818 | 0.3022305 | 0.00395 | $$ |  |
| Carlos | PR | Discrimination Ratio | Genotype x Session | GLMM with beta regression | 0.10261 | -0.07497597 | 0.2801917 | 0.25744 | n.s. |  |
| Carlos | FR1 | Discrimination Ratio | Genotype | GLM with zero-inflated beta regression | 0.6351 | -0.549876 | 1.820119 | 0.293 | n.s. |  |
| Carlos | FR2 | Discrimination Ratio | Genotype | GLM with zero-inflated beta regression | -0.1301 | -0.7670557 | 0.5068222 | 0.689 | n.s. |  |
| Carlos | FR3 | Discrimination Ratio | Genotype | GLM with zero-inflated beta regression | 0.1482 | -0.5445879 | 0.8410772 | 0.675 | n.s. |  |
| Carlos | FR4 | Discrimination Ratio | Genotype | GLM with zero-inflated beta regression | 0.2061 | -0.515344 | 0.9276261 | 0.575 | n.s. |  |
| Carlos | FR5 ALL | Discrimination Ratio | Genotype | GLMM with zero-inflated beta regression | 0.3362 | -0.05012378 | 1.0340345 | 0.0753 | n.s. |  |
| Carlos | FR5 ALL | Discrimination Ratio | Session | GLMM with zero-inflated beta regression | 0.08939 | -0.09588575 | 0.2746622 | 0.3443 | n.s. |  |
| Carlos | FR5 ALL | Discrimination Ratio | Genotype x Session | GLMM with zero-inflated beta regression | -0.10846 | -0.34571271 | 0.1288014 | 0.3703 | n.s. |  |
| Carlos | FR5 BEFORE PR | Discrimination Ratio | Genotype | GLMM with zero-inflated beta regression | 0.63123 | -0.01509684 | 1.27756615 | 0.0556 | n.s. |  |
| Carlos | FR5 BEFORE PR | Discrimination Ratio | Session | GLMM with zero-inflated beta regression | -0.16815 | -0.42003585 | 0.08372944 | 0.1907 | n.s. |  |
| Carlos | FR5 BEFORE PR | Discrimination Ratio | Genotype x Session | GLMM with zero-inflated beta regression | 0.09529 | -0.20728403 | 0.39785876 | 0.5371 | n.s. |  |
| Carlos | FR5 AFTER PR | Discrimination Ratio | Genotype | GLMM with zero-inflated beta regression | 0.29069 | -0.3499065 | 0.931294 | 0.374 | n.s. |  |
| Carlos | FR5 AFTER PR | Discrimination Ratio | Session | GLMM with zero-inflated beta regression | -0.22373 | -0.551947 | 0.1044792 | 0.182 | n.s. |  |
| Carlos | FR5 AFTER PR | Discrimination Ratio | Genotype x Session | GLMM with zero-inflated beta regression | 0.09812 | -0.3141687 | 0.5104066 | 0.641 | n.s. |  |
| Carlos | FR10 | Discrimination Ratio | Genotype | GLMM with zero-inflated beta regression | 0.7814 | 0.18854915 | -3.0052556 | 0.00979 | ✱✱ |  |
| Carlos | FR10 | Discrimination Ratio | Session | GLMM with zero-inflated beta regression | -0.4171 | -0.72538603 | 1.3743452 | 0.008 | $$ |  |
| Carlos | FR10 | Discrimination Ratio | Genotype x Session | GLMM with zero-inflated beta regression | 0.3979 | 0.02025557 | -0.1088484 | 0.03891 | # | **WT - KI Session 1** P=0.2560  **WT - KI Session 2** P=0.2595  **WT - KI Session 3** P=0.0002 (***) |
| Carlos | FR20 | Discrimination Ratio | Genotype | GLMM with zero-inflated beta regression | 1.5786 | 0.9077024 | 2.2494867 | 0.000004 | ✱✱✱ |  |
| Carlos | FR20 | Discrimination Ratio | Session | GLMM with zero-inflated beta regression | -0.3111 | -0.5514861 | -0.07070935 | 0.0112 | $ |  |
| Carlos | FR20 | Discrimination Ratio | Genotype x Session | GLMM with zero-inflated beta regression | 0.1809 | -0.108152 | 0.46987906 | 0.22 | n.s. |  |
| Carlos | FR40 | Discrimination Ratio | Genotype | GLMM with zero-inflated beta regression | 1.53853 | 0.787441819 | 2.2896157 | 0.00006 | ✱✱✱ |  |
| Carlos | FR40 | Discrimination Ratio | Session | GLMM with zero-inflated beta regression | 0.16561 | -0.005963872 | 0.3371922 | 0.0585 | n.s. |  |
| Carlos | FR40 | Discrimination Ratio | Genotype x Session | GLMM with zero-inflated beta regression | 0.03675 | -0.17089141 | 0.2443874 | 0.7287 | n.s. |  |
| Carlos | PR | Magazine Entry Rate | Genotype | GLMM with beta regression | 0.32225 | -0.04866833 | 0.693171 | 0.0886 | n.s. |  |
| Carlos | PR | Magazine Entry Rate | Session | GLMM with beta regression | -0.28523 | -0.42454749 | -0.1459222 | 0.0001 | $$$ |  |
| Carlos | PR | Magazine Entry Rate | Genotype x Session | GLMM with beta regression | 0.1999 | 0.0136733 | 0.3861275 | 0.0354 | # | **WT - KI Session 1** P=0.6203  **WT - KI Session 2** P=0.1681  **WT - KI Session 3** P=0.1367  **WT - KI Session 4** P=0.3513  **WT - KI Session 5** P=0.2037  **WT - KI Session 6** P=0.0125 (*) |
| Carlos | FR1 | Magazine Entry Rate | Genotype | GLM with beta regression | -0.02114 | -0.2845351 | 0.242261 | 0.875 | n.s. |  |
| Carlos | FR2 | Magazine Entry Rate | Genotype | GLM with beta regression | -0.04762 | -0.2384245 | 0.1431808 | 0.625 | n.s. |  |
| Carlos | FR3 | Magazine Entry Rate | Genotype | GLM with beta regression | -0.1937 | -0.3463014 | -0.04110082 | 0.0129 | ✱ |  |
| Carlos | FR4 | Magazine Entry Rate | Genotype | GLM with beta regression | -0.11421 | -0.2510549 | 0.022639 | 0.102 | n.s. |  |
| Carlos | FR5 ALL | Magazine Entry Rate | Genotype | GLMM with beta regression | -0.055449 | -0.1618401 | 0.05094243 | 0.307 | n.s. |  |
| Carlos | FR5 ALL | Magazine Entry Rate | Session | GLMM with beta regression | 0.090905 | 0.0638733 | 0.11793668 | 0.0000000 | $$$ |  |
| Carlos | FR5 ALL | Magazine Entry Rate | Genotype x Session | GLMM with beta regression | 0.006839 | -0.0337600 | 0.04743772 | 0.741 | n.s. |  |
| Carlos | FR5 BEFORE PR | Magazine Entry Rate | Genotype | GLMM with beta regression | -0.060781 | -0.18594966 | 0.064387279 | 0.3412 | n.s. |  |
| Carlos | FR5 BEFORE PR | Magazine Entry Rate | Session | GLMM with beta regression | -0.038008 | -0.06932976 | -0.006685347 | 0.0174 | $ |  |
| Carlos | FR5 BEFORE PR | Magazine Entry Rate | Genotype x Session | GLMM with beta regression | -0.005899 | -0.05331783 | 0.041520734 | 0.8074 | n.s. |  |
| Carlos | FR5 AFTER PR | Magazine Entry Rate | Genotype | GLMM with beta regression | -0.045596 | -0.14819583 | 0.05700434 | 0.384 | n.s. |  |
| Carlos | FR5 AFTER PR | Magazine Entry Rate | Session | GLMM with beta regression | 0.01729 | -0.01319385 | 0.04777427 | 0.266 | n.s. |  |
| Carlos | FR5 AFTER PR | Magazine Entry Rate | Genotype x Session | GLMM with beta regression | -0.003829 | -0.04952775 | 0.0418702 | 0.87 | n.s. |  |
| Carlos | FR10 | Magazine Entry Rate | Genotype | GLMM with zero-inflated beta regression | -0.207384 | -0.4116580 | -0.0031106 | 0.0466 | ✱ |  |
| Carlos | FR10 | Magazine Entry Rate | Session | GLMM with zero-inflated beta regression | -0.005448 | -0.0716507 | 0.0607557 | 0.8719 | n.s. |  |
| Carlos | FR10 | Magazine Entry Rate | Genotype x Session | GLMM with zero-inflated beta regression | 0.013342 | -0.0898477 | 0.1165326 | 0.7999 | n.s. |  |
| Carlos | FR20 | Magazine Entry Rate | Genotype | GLMM with zero-inflated beta regression | -0.0767 | -0.30921872 | 0.155826695 | 0.518 | n.s. |  |
| Carlos | FR20 | Magazine Entry Rate | Session | GLMM with zero-inflated beta regression | 0.06911 | -0.02036348 | 0.158581249 | 0.1301 | n.s. |  |
| Carlos | FR20 | Magazine Entry Rate | Genotype x Session | GLMM with zero-inflated beta regression | -0.14302 | -0.28056508 | -0.005472648 | 0.0416 | # | **WT - KI Session 1** P=0.5260  **WT - KI Session 2** P=0.6267  **WT - KI Session 3** P=0.1059 |
| Carlos | FR40 | Magazine Entry Rate | Genotype | GLMM with beta regression | 0.34847 | -0.1105952 | 0.8075264 | 0.1368 | n.s. |  |
| Carlos | FR40 | Magazine Entry Rate | Session | GLMM with beta regression | -0.09075 | -0.19521462 | 0.0137104 | 0.0886 | n.s. |  |
| Carlos | FR40 | Magazine Entry Rate | Genotype x Session | GLMM with beta regression | 0.07575 | -0.07272044 | 0.2242286 | 0.3173 | n.s. |  |
| Carlos | PR | Magazine Entries | Genotype | GLMM with negative binomial regression | 0.1741 | -0.1464884 | 0.49468567 | 0.28716 | n.s. |  |
| Carlos | PR | Magazine Entries | Session | GLMM with negative binomial regression | -0.18545 | -0.3260055 | -0.04489327 | 0.00971 | $$ |  |
| Carlos | PR | Magazine Entries | Genotype x Session | GLMM with negative binomial regression | 0.03824 | -0.1612169 | 0.23770019 | 0.70708 | n.s. |  |
| Carlos | FR1 | Magazine Entries | Genotype | GLM with negative binomial regression | -0.002862 | 3.4175203 | 4.1394654 | 0.991 | n.s. |  |
| Carlos | FR2 | Magazine Entries | Genotype | GLM with negative binomial regression | 0.214 | 0.06476086 | 0.3632359 | 0.00495 | ✱✱ |  |
| Carlos | FR3 | Magazine Entries | Genotype | GLM with negative binomial regression | 0.04015 | -0.1010169 | 0.1813109 | 0.577 | n.s. |  |
| Carlos | FR4 | Magazine Entries | Genotype | GLM with negative binomial regression | -0.04794 | -0.1911681 | 0.09528872 | 0.512 | n.s. |  |
| Carlos | FR5 ALL | Magazine Entries | Genotype | GLMM with negative binomial regression | 0.049795 | -0.02632331 | 0.12591284 | 0.2 | n.s. |  |
| Carlos | FR5 ALL | Magazine Entries | Session | GLMM with negative binomial regression | 0.000595 | -0.03935737 | 0.04054734 | 0.977 | n.s. |  |
| Carlos | FR5 ALL | Magazine Entries | Genotype x Session | GLMM with negative binomial regression | 0.011159 | -0.04719792 | 0.06951676 | 0.708 | n.s. |  |
| Carlos | FR5 BEFORE PR | Magazine Entries | Genotype | GLMM with poisson regression | 0.038476 | -0.04322171 | 0.12017389 | 0.356 | n.s. |  |
| Carlos | FR5 BEFORE PR | Magazine Entries | Session | GLMM with poisson regression | -0.004178 | -0.05322836 | 0.04487323 | 0.867 | n.s. |  |
| Carlos | FR5 BEFORE PR | Magazine Entries | Genotype x Session | GLMM with poisson regression | -0.023798 | -0.0958037 | 0.0482083 | 0.517 | n.s. |  |
| Carlos | FR5 AFTER PR | Magazine Entries | Genotype | GLMM with poisson regression | 0.06959 | -0.03073878 | 0.16991318 | 0.174 | n.s. |  |
| Carlos | FR5 AFTER PR | Magazine Entries | Session | GLMM with poisson regression | 0.00125 | -0.06845498 | 0.07095576 | 0.972 | n.s. |  |
| Carlos | FR5 AFTER PR | Magazine Entries | Genotype x Session | GLMM with poisson regression | -0.02784 | -0.12932557 | 0.07364614 | 0.591 | n.s. |  |
| Carlos | FR10 | Magazine Entries | Genotype | GLMM with negative binomial regression | 0.30936 | 0.17236349 | 0.4463634 | 0.00001 | ✱✱✱ |  |
| Carlos | FR10 | Magazine Entries | Session | GLMM with negative binomial regression | -0.04763 | -0.11386221 | 0.01860675 | 0.159 | n.s. |  |
| Carlos | FR10 | Magazine Entries | Genotype x Session | GLMM with negative binomial regression | -0.03402 | -0.12694778 | 0.05891225 | 0.473 | n.s. |  |
| Carlos | FR20 | Magazine Entries | Genotype | GLMM with negative binomial regression | 0.43307 | 0.1846225 | 0.68151815 | 0.000635 | ✱✱✱ |  |
| Carlos | FR20 | Magazine Entries | Session | GLMM with negative binomial regression | -0.01968 | -0.1346709 | 0.09531435 | 0.737322 | n.s. |  |
| Carlos | FR20 | Magazine Entries | Genotype x Session | GLMM with negative binomial regression | -0.18142 | -0.3496185 | -0.013212 | 0.034523 | # | **WT - KI Session 1** P=0.0002 (***)  **WT - KI Session 2** P=0.0132 (*)  **WT - KI Session 3** P=0.2254 |
| Carlos | FR40 | Magazine Entries | Genotype | GLMM with negative binomial regression | 0.41714 | -0.0235685 | 0.857853748 | 0.0636 | n.s. |  |
| Carlos | FR40 | Magazine Entries | Session | GLMM with negative binomial regression | -0.11136 | -0.2150931 | -0.007631725 | 0.0354 | $ |  |
| Carlos | FR40 | Magazine Entries | Genotype x Session | GLMM with negative binomial regression | 0.1141 | -0.04737025 | 0.275570064 | 0.1661 | n.s. |  |
| Carlos | PR | Reward Collection Latency | Genotype | GLMM with log-adjusted inverse gaussian regression | -0.109384 | -0.20257332 | -0.01619483 | 0.021416 | ✱ |  |
| Carlos | PR | Reward Collection Latency | Session | GLMM with log-adjusted inverse gaussian regression | -0.033687 | -0.06126555 | -0.00610846 | 0.01666200 | $ |  |
| Carlos | PR | Reward Collection Latency | Genotype x Session | GLMM with inverse gaussian regression | 0.009358 | -0.03174913 | 0.05046464 | 0.655471 | n.s. |  |
| Carlos | FR1 | Reward Collection Latency | Genotype | GLMM with inverse gaussian regression | 0.33302 | 0.0729914 | 0.5930574 | 0.0121 | ✱ |  |
| Carlos | FR2 | Reward Collection Latency | Genotype | GLMM with inverse gaussian regression | 0.2215 | 0.07094858 | 0.3720524 | 0.00393 | ✱✱ |  |
| Carlos | FR3 | Reward Collection Latency | Genotype | GLMM with log-adjusted inverse gaussian regression | 0.11367 | -0.08558559 | 0.3129335 | 0.264 | n.s. |  |
| Carlos | FR4 | Reward Collection Latency | Genotype | GLMM with inverse gaussian regression | 0.24781 | 0.0851444 | 0.4104741 | 0.00283 | ✱✱ |  |
| Carlos | FR5 ALL | Reward Collection Latency | Genotype | GLMM with log-adjusted inverse gaussian regression | -0.16977 | -0.285061876 | -0.05448545 | 0.0039 | ✱✱ |  |
| Carlos | FR5 ALL | Reward Collection Latency | Session | GLMM with log-adjusted inverse gaussian regression | -0.07352 | -0.092980932 | -0.05405553 | 1.33E-13 | $$$ |  |
| Carlos | FR5 ALL | Reward Collection Latency | Genotype x Session | GLMM with log-adjusted inverse gaussian regression | 0.02233 | -0.006955727 | 0.05161836 | 0.13505 | n.s. |  |
| Carlos | FR5 BEFORE PR | Reward Collection Latency | Genotype | GLMM with inverse gaussian regression | 0.27519 | 0.1307648 | 0.41961881 | 0.000188 | ✱✱✱ |  |
| Carlos | FR5 BEFORE PR | Reward Collection Latency | Session | GLMM with inverse gaussian regression | -0.01264 | -0.04786354 | 0.02257871 | 0.481733 | n.s. |  |
| Carlos | FR5 BEFORE PR | Reward Collection Latency | Genotype x Session | GLMM with inverse gaussian regression | 0.02618 | -0.02263425 | 0.07500362 | 0.293142 | n.s. |  |
| Carlos | FR5 AFTER PR | Reward Collection Latency | Genotype | GLMM with inverse gaussian regression | 0.20262 | -0.03171343 | 0.43695667 | 0.09013 | n.s. |  |
| Carlos | FR5 AFTER PR | Reward Collection Latency | Session | GLMM with inverse gaussian regression | -0.01465 | -0.07178084 | 0.04248149 | 0.61526 | n.s. |  |
| Carlos | FR5 AFTER PR | Reward Collection Latency | Genotype x Session | GLMM with inverse gaussian regression | 0.107 | 0.02751424 | 0.18648038 | 0.00833 | ## | **WT - KI Session 1** P=0.3922  **WT - KI Session 2** P=0.0261 (*) |
| Carlos | FR10 | Reward Collection Latency | Genotype | GLMM with inverse gaussian regression | 0.2002051 | -0.007948113 | 0.40835824 | 0.0594 | n.s. |  |
| Carlos | FR10 | Reward Collection Latency | Session | GLMM with inverse gaussian regression | 0.0205554 | -0.022347288 | 0.06345809 | 0.3477 | n.s. |  |
| Carlos | FR10 | Reward Collection Latency | Genotype x Session | GLMM with inverse gaussian regression | 0.0009309 | -0.059766123 | 0.06162792 | 0.976 | n.s. |  |
| Carlos | FR20 | Reward Collection Latency | Genotype | GLMM with log-adjusted inverse gaussian regression | -0.21145 | -0.33349029 | -0.08940539 | 0.000684 | ✱✱✱ |  |
| Carlos | FR20 | Reward Collection Latency | Session | GLMM with log-adjusted inverse gaussian regression | 0.0754 | 0.03899267 | 0.11181696 | 4.93E-05 | $$$ |  |
| Carlos | FR20 | Reward Collection Latency | Genotype x Session | GLMM with log-adjusted inverse gaussian regression | -0.07164 | -0.12560432 | -0.01767769 | 0.009268 | ## | **WT - KI Session 1** P =0.0335 (*)  **WT - KI Session 2** P=0.0017 (**)  **WT - KI Session 3** P=0.0004 (***) |
| Carlos | FR40 | Reward Collection Latency | Genotype | GLMM with inverse gaussian regression | 0.25613 | 0.04908192 | 0.46317377 | 0.0153 | ✱ |  |
| Carlos | FR40 | Reward Collection Latency | Session | GLMM with inverse gaussian regression | 0.02659 | -0.007245562 | 0.06042659 | 0.1235 | n.s. |  |
| Carlos | FR40 | Reward Collection Latency | Genotype x Session | GLMM with inverse gaussian regression | -0.01216 | -0.070063277 | 0.04574978 | 0.6807 | n.s. |  |
| Carlos | PR | Schedule Length | Genotype | Cox proportional hazards model | 0.8293 | 1.184 | 4.436 | 0.0138 | ✱ |  |
| Carlos | PR | Schedule Length | Session | Cox proportional hazards model | -0.2026 | 0.5302 | 1.258 | 0.3578 | n.s. |  |
| Carlos | PR | Schedule Length | Genotype x Session | Cox proportional hazards model | -0.2026 | 0.7287 | 2.202 | 0.4022 | n.s. |  |
| Carlos | FR1 | Schedule Length | Genotype | Cox proportional hazards model | -0.2258 | 0.3826 | 1.664 | 0.547 | n.s. |  |
| Carlos | FR2 | Schedule Length | Genotype | Cox proportional hazards model | -1.0789 | 0.1324 | 0.8726 | 0.0249 | ✱ |  |
| Carlos | FR3 | Schedule Length | Genotype | Cox proportional hazards model | -1.2813 | 0.09639 | 0.7999 | 0.0176 | ✱ |  |
| Carlos | FR4 | Schedule Length | Genotype | Cox proportional hazards model | -0.1662 | 0.3705 | 1.936 | 0.694 | n.s. |  |
| Carlos | FR5 ALL | Schedule Length | Genotype | Cox proportional hazards model | -0.58753 | 0.2821 | 1.0948 | 0.0895 | n.s. |  |
| Carlos | FR5 ALL | Schedule Length | Session | Cox proportional hazards model | 0.76884 | 1.7861 | 2.6056 | 0.00000000 | $$$ |  |
| Carlos | FR5 ALL | Schedule Length | Genotype x Session | Cox proportional hazards model | -0.26192 | 0.6162 | 0.9611 | 0.02090000 | # | **WT - KI Session 1** P=0.1108  **WT - KI Session 2** P=0.5535  **WT - KI Session 3** P=0.2481  **WT - KI Session 4** P=0.7281  **WT - KI Session 5** P=0.0012 (**)  **WT - KI Session 6** P=0.1356 |
| Carlos | FR5 BEFORE PR | Schedule Length | Genotype | Cox proportional hazards model | -0.4198 | 0.3155 | 1.369 | 0.262 | n.s. |  |
| Carlos | FR5 BEFORE PR | Schedule Length | Session | Cox proportional hazards model | -0.1793 | 0.6713 | 1.041 | 0.109 | n.s. |  |
| Carlos | FR5 BEFORE PR | Schedule Length | Genotype x Session | Cox proportional hazards model | 0.1674 | 0.8733 | 1.601 | 0.279 | n.s. |  |
| Carlos | FR5 AFTER PR | Schedule Length | Genotype | Cox proportional hazards model | -0.80643 | 0.2182 | 0.9133 | 0.0272 | ✱ |  |
| Carlos | FR5 AFTER PR | Schedule Length | Session | Cox proportional hazards model | 0.06645 | 0.7689 | 1.4854 | 0.6924 | n.s. |  |
| Carlos | FR5 AFTER PR | Schedule Length | Genotype x Session | Cox proportional hazards model | 0.25679 | 0.8146 | 2.0517 | 0.2758 | n.s. |  |
| Carlos | FR10 | Schedule Length | Genotype | Cox proportional hazards model | -1.75123 | 0.09134 | 0.3298 | 0.00000009 | ✱✱✱ |  |
| Carlos | FR10 | Schedule Length | Session | Cox proportional hazards model | 0.2238 | 0.94095 | 1.6627 | 0.123 | n.s. |  |
| Carlos | FR10 | Schedule Length | Genotype x Session | Cox proportional hazards model | 0.05792 | 0.65185 | 1.7225 | 0.815 | n.s. |  |
| Carlos | FR20 | Schedule Length | Genotype | Cox proportional hazards model | -1.69463 | 0.081 | 0.4165 | 0.00005 | ✱✱✱ |  |
| Carlos | FR20 | Schedule Length | Session | Cox proportional hazards model | 0.21123 | 0.8695 | 1.7548 | 0.238 | n.s. |  |
| Carlos | FR20 | Schedule Length | Genotype x Session | Cox proportional hazards model | -0.00418 | 0.6658 | 1.4895 | 0.984 | n.s. |  |
| Carlos | FR40 | Schedule Length | Genotype | Cox proportional hazards model | -20.19 | 0.0000000 | 0.0000000 | 0.0000000 | ✱✱✱ |  |
| Carlos | FR40 | Schedule Length | Session | Cox proportional hazards model | -0.5202000 | 0.2363000 | 1.4950000 | 0.269 | n.s. |  |
| Carlos | FR40 | Schedule Length | Genotype x Session | Cox proportional hazards model | 0.5202000 | 0.6657000 | 4.2520000 | 0.271 | n.s. |  |
| Carlos | PR | Target Touch Rate | Genotype | GLMM with beta regression | -0.52607 | -0.7326729 | -0.3194664 | 6.02E-07 | ✱✱✱ |  |
| Carlos | PR | Target Touch Rate | Session | GLMM with beta regression | -0.20651 | -0.2798796 | -0.1331493 | 3.45E-08 | $$$ |  |
| Carlos | PR | Target Touch Rate | Genotype x Session | GLMM with beta regression | -0.01236 | -0.1330014 | 0.1082763 | 0.841 | n.s. |  |
| Carlos | FR1 | Target Touch Rate | Genotype | GLM with beta regression | -0.2798 | -0.5725271 | 0.01292104 | 0.061 | n.s. |  |
| Carlos | FR2 | Target Touch Rate | Genotype | GLM with beta regression | -0.26936 | -0.4890314 | -0.04969375 | 0.0162 | ✱ |  |
| Carlos | FR3 | Target Touch Rate | Genotype | GLM with beta regression | -0.26735 | -0.4746695 | -0.06002216 | 0.0115 | ✱ |  |
| Carlos | FR4 | Target Touch Rate | Genotype | GLM with beta regression | -0.0937 | -0.2986089 | 0.1111996 | 0.37 | n.s. |  |
| Carlos | FR5 ALL | Target Touch Rate | Genotype | GLMM with beta regression | -0.15589 | -0.30397053 | -0.007801025 | 0.0391 | ✱ |  |
| Carlos | FR5 ALL | Target Touch Rate | Session | GLMM with beta regression | 0.13127 | 0.09843284 | 0.164099154 | 4.66E-15 | $$$ |  |
| Carlos | FR5 ALL | Target Touch Rate | Genotype x Session | GLMM with beta regression | -0.01391 | -0.06312395 | 0.035301755 | 0.5796 | n.s. |  |
| Carlos | FR5 BEFORE PR | Target Touch Rate | Genotype | GLMM with beta regression | -0.14294 | -0.3155637 | 0.02968915 | 0.10462 | n.s. |  |
| Carlos | FR5 BEFORE PR | Target Touch Rate | Session | GLMM with beta regression | 0.0287 | -0.0859729 | -0.01668847 | 0.00368 | $$ |  |
| Carlos | FR5 BEFORE PR | Target Touch Rate | Genotype x Session | GLMM with beta regression | 0.0287 | -0.02325777 | 0.08066071 | 0.27896 | n.s. |  |
| Carlos | FR5 AFTER PR | Target Touch Rate | Genotype | GLMM with beta regression | -0.18179 | -0.31049822 | -0.05307328 | 0.00564 | ✱✱ |  |
| Carlos | FR5 AFTER PR | Target Touch Rate | Session | GLMM with beta regression | 0.02777 | -0.02597175 | 0.08151245 | 0.31117 | n.s. |  |
| Carlos | FR5 AFTER PR | Target Touch Rate | Genotype x Session | GLMM with beta regression | 0.03225 | -0.04832052 | 0.1128279 | 0.43271 | n.s. |  |
| Carlos | FR10 | Target Touch Rate | Genotype | GLMM with beta regression | -0.74922 | -1.0182092 | -0.48022395 | 4.79E-08 | ✱✱✱ |  |
| Carlos | FR10 | Target Touch Rate | Session | GLMM with beta regression | 0.08089 | -0.02906517 | 0.19085057 | 0.149 | n.s. |  |
| Carlos | FR10 | Target Touch Rate | Genotype x Session | GLMM with beta regression | 0.0136 | -0.15735364 | 0.18455137 | 0.876 | n.s. |  |
| Carlos | FR20 | Target Touch Rate | Genotype | GLMM with beta regression | -1.01104 | -1.442597592 | -0.5794917 | 4.39E-06 | ✱✱✱ |  |
| Carlos | FR20 | Target Touch Rate | Session | GLMM with beta regression | 0.09984 | -0.005060355 | 0.2047481 | 0.06212 | n.s. |  |
| Carlos | FR20 | Target Touch Rate | Genotype x Session | GLMM with beta regression | 0.02532 | -0.148674918 | 0.1993122 | 0.77549 | n.s. |  |
| Carlos | FR40 | Target Touch Rate | Genotype | GLMM with beta regression | -0.92877 | -1.4487543 | -0.4087796 | 0.000464 | ✱✱✱ |  |
| Carlos | FR40 | Target Touch Rate | Session | GLMM with beta regression | -0.14114 | -0.21723583 | -0.0650401 | 0.000278 | $$$ |  |
| Carlos | FR40 | Target Touch Rate | Genotype x Session | GLMM with beta regression | 0.04401 | -0.09587436 | 0.1838886 | 0.537492 | n.s. |  |
| Carlos | PR | Target Touches | Genotype | GLMM with negative binomial regression | -0.66243 | -0.9922166 | -0.332642584 | 8.25E-05 | ✱✱✱ |  |
| Carlos | PR | Target Touches | Session | GLMM with negative binomial regression | -0.14413 | -0.233044 | -0.055213215 | 0.00149 | $$ |  |
| Carlos | PR | Target Touches | Genotype x Session | GLMM with negative binomial regression | -0.13467 | -0.2648621 | -0.004477495 | 0.04262 | # | **WT - KI Session 1** P=0.2754  **WT - KI Session 2**  P=0.0009 (***)  **WT - KI Session 3** P=0.0006 (***)  **WT - KI Session 4** P=0.0004 (***)  **WT - KI Session 5** P=0.0002 (***)  **WT - KI Session 6** P=0.0011 (**) |
| Carlos | FR1 | Target Touches | Genotype | GLM with negative binomial regression | -0.2336 | -0.6323198 | 0.16509 | 0.251 | n.s. |  |
| Carlos | FR2 | Target Touches | Genotype | GLM with poisson regression | 1.01E-10 | -0.1083411 | 0.1083411 | 1 | n.s. |  |
| Carlos | FR3 | Target Touches | Genotype | GLM with poisson regression | 4.05E-08 | -0.08846008 | 0.08846016 | 1 | n.s. |  |
| Carlos | FR4 | Target Touches | Genotype | GLM with poisson regression | 1.94E-09 | -0.07660871 | 0.07660871 | 1 | n.s. |  |
| Carlos | FR5 ALL | Target Touches | Genotype | GLM with poisson regression | -5.40E-10 | -0.02797355 | 0.02797355 | 1 | n.s. |  |
| Carlos | FR5 ALL | Target Touches | Session | GLM with poisson regression | -1.97E-10 | -0.01893161 | 0.01893161 | 1 | n.s. |  |
| Carlos | FR5 ALL | Target Touches | Genotype x Session | GLM with poisson regression | -1.47E-09 | -0.02808011 | 0.02808011 | 1 | n.s. |  |
| Carlos | FR5 BEFORE PR | Target Touches | Genotype | GLM with poisson regression | 7.00E-15 | -0.03426046 | 0.03426046 | 1 | n.s. |  |
| Carlos | FR5 BEFORE PR | Target Touches | Session | GLM with poisson regression | 4.40E-15 | -0.02323076 | 0.02323076 | 1 | n.s. |  |
| Carlos | FR5 BEFORE PR | Target Touches | Genotype x Session | GLM with poisson regression | -9.27E-15 | -0.03445679 | 0.03445679 | 1 | n.s. |  |
| Carlos | FR5 AFTER PR | Target Touches | Genotype | GLM with poisson regression | 1.83E-13 | -0.0484516 | 0.0484516 | 1 | n.s. |  |
| Carlos | FR5 AFTER PR | Target Touches | Session | GLM with poisson regression | 0.00E+00 | -0.03304372 | 0.03304372 | 1 | n.s. |  |
| Carlos | FR5 AFTER PR | Target Touches | Genotype x Session | GLM with poisson regression | 0.00E+00 | -0.04901176 | 0.04901176 | 1 | n.s. |  |
| Carlos | FR10 | Target Touches | Genotype | GLM with poisson regression | -2.50E-09 | -0.02797355 | 0.02797354 | 1 | n.s. |  |
| Carlos | FR10 | Target Touches | Session | GLM with poisson regression | 0.00E+00 | -0.01900428 | 0.01900428 | 1 | n.s. |  |
| Carlos | FR10 | Target Touches | Genotype x Session | GLM with poisson regression | 0.00E+00 | -0.0281879 | 0.0281879 | 1 | n.s. |  |
| Carlos | FR20 | Target Touches | Genotype | GLM with poisson regression | -2.19E-01 | -0.3825734 | -0.054907125 | 0.00888 | ✱✱ |  |
| Carlos | FR20 | Target Touches | Session | GLM with poisson regression | -9.41E-10 | -0.01343806 | 0.013438058 | 1 | n.s. |  |
| Carlos | FR20 | Target Touches | Genotype x Session | GLM with poisson regression | -1.65E-02 | -0.03753519 | 0.004451632 | 0.1225 | n.s. |  |
| Carlos | FR40 | Target Touches | Genotype | GLM with poisson regression | -0.715846 | -1.101522389 | -0.33017004 | 0.000275 | ✱✱✱ |  |
| Carlos | FR40 | Target Touches | Session | GLM with poisson regression | -0.107594 | -0.118976615 | -0.09621197 | 2.00E-16 | $$$ |  |
| Carlos | FR40 | Target Touches | Genotype x Session | GLM with poisson regression | 0.029142 | 0.007564923 | 0.05071952 | 0.008118 | ## | **WT - KI Session 1** P=0.0001 (***)  **WT - KI Session 2** P=0.0003 (***)  **WT - KI Session 3** P=0.0005 (***) |

**Figures 3 and 4**.

| **Experimenter** | **Stage** | **Dependent Variable** | **Independent Variable** | **Stats Test** | **Coefficient** | **Lower 2.5% CI** | **Upper 97.5% CI** | **P-Value** | **Stats Summary** | **Pairwise Notes** |
| --- | --- | --- | --- | --- | --- | --- | --- | --- | --- | --- |
| Julia | PR | Blank Touch Rate | Genotype | GLMM with zero-inflated beta regression | 0.09461 | -0.288661 | 0.47787376 | 0.629 | n.s. |  |
| Julia | PR | Blank Touch Rate | Session | GLMM with zero-inflated beta regression | 0.02426 | -0.07790715 | 0.12642084 | 0.642 | n.s. |  |
| Julia | PR | Blank Touch Rate | Genotype x Session | GLMM with zero-inflated beta regression | -0.09763 | -0.23465256 | 0.03940245 | 0.629 | n.s. |  |
| Julia | PR + ATO | Blank Touch Rate | Genotype | GLMM with zero-inflated beta regression | 0.1123 | -0.2525627 | 0.4772036 | 0.546 | n.s. |  |
| Julia | PR + ATO | Blank Touch Rate | Drug | GLMM with zero-inflated beta regression | -0.6032 | -0.8567133 | -0.3496172 | 3.12E-06 | @@@ |  |
| Julia | PR + ATO | Blank Touch Rate | Genotype x Drug | GLMM with zero-inflated beta regression | 0.2053 | -0.1251509 | 0.5356941 | 0.223 | n.s. |  |
| Julia | PR + MPH | Blank Touch Rate | Genotype | GLMM with zero-inflated beta regression | 0.14347 | -0.245361 | 0.5322929 | 0.46958 | n.s. |  |
| Julia | PR + MPH | Blank Touch Rate | Drug | GLMM with zero-inflated beta regression | 0.32807 | 0.1131751 | 0.5429667 | 0.00277 | @@ |  |
| Julia | PR + MPH | Blank Touch Rate | Genotype x Drug | GLMM with zero-inflated beta regression | -0.07826 | -0.3683839 | 0.2118676 | 0.59703 | n.s. |  |
| Julia | FR1 | Blank Touch Rate | Genotype | GLMM with zero-inflated beta regression | -0.27892 | -0.9571208 | 0.39928204 | 0.42 | n.s. |  |
| Julia | FR1 | Blank Touch Rate | Session | GLMM with zero-inflated beta regression | 0.07874 | -0.1656143 | 0.32308543 | 0.528 | n.s. |  |
| Julia | FR1 | Blank Touch Rate | Genotype x Session | GLMM with zero-inflated beta regression | 0.02331 | -0.3162681 | 0.36288797 | 0.893 | n.s. |  |
| Julia | FR2 | Blank Touch Rate | Genotype | GLMM with zero-inflated beta regression | -0.12079 | -0.5448163 | 0.303228 | 0.577 | n.s. |  |
| Julia | FR2 | Blank Touch Rate | Session | GLMM with zero-inflated beta regression | -0.10296 | -0.4171665 | 0.2112551 | 0.521 | n.s. |  |
| Julia | FR2 | Blank Touch Rate | Genotype x Session | GLMM with zero-inflated beta regression | 0.02842 | -0.3988616 | 0.4556944 | 0.896 | n.s. |  |
| Julia | FR3 | Blank Touch Rate | Genotype | GLM with zero-inflated beta regression | -0.2286 | -0.7722791 | 0.3151522 | 0.41 | n.s. |  |
| Julia | FR5 ALL | Blank Touch Rate | Genotype | GLM with zero-inflated beta regression | 0.52682 | 0.14262755 | 0.91101683 | 0.0072 | ✱✱ |  |
| Julia | FR5 ALL | Blank Touch Rate | Session | GLM with zero-inflated beta regression | -0.19706 | -0.33327138 | -0.06084624 | 0.00458 | $$ |  |
| Julia | FR5 ALL | Blank Touch Rate | Genotype x Session | GLM with zero-inflated beta regression | 0.06878 | -0.08513614 | 0.22269897 | 0.38111 | n.s. |  |
| Julia | FR5 BEFORE PR + ATO | Blank Touch Rate | Genotype | GLM with zero-inflated beta regression | 0.5236 | 0.08547612 | 0.9618071 | 0.019165 | ✱ |  |
| Julia | FR5 BEFORE PR + ATO | Blank Touch Rate | Session | GLM with zero-inflated beta regression | -0.3126 | -0.45199092 | -0.1732831 | 1.10E-05 | $$$ |  |
| Julia | FR5 BEFORE PR + ATO | Blank Touch Rate | Genotype x Session | GLM with zero-inflated beta regression | 0.3079 | 0.13523678 | 0.4805741 | 0.000474 | ### | **WT - KI Session 1** P=0.8047  **WT - KI Session 2** P=0.1234  **WT - KI Session 3** P=0.9505  **WT - KI Session 4** P=0.0821  **WT - KI Session 5** 0.0106 (*)  **WT - KI Session 6** P=0.0089 (**)  **WT - KI Session 7** P=0.0071 (**) |
| Julia | FR5 DURING PR + ATO | Blank Touch Rate | Genotype | GLM with zero-inflated beta regression | 0.51706 | -0.05582527 | 1.0899368 | 0.0769 | n.s. |  |
| Julia | FR5 DURING PR + ATO | Blank Touch Rate | Session | GLM with zero-inflated beta regression | -0.03275 | -0.265847 | 0.2003393 | 0.783 | n.s. |  |
| Julia | FR5 DURING PR + ATO | Blank Touch Rate | Genotype x Session | GLM with zero-inflated beta regression | -0.04231 | -0.29771951 | 0.2130958 | 0.7454 | n.s. |  |
| Julia | FR5 DURING PR + MPH | Blank Touch Rate | Genotype | GLM with zero-inflated beta regression | 0.61519 | 0.1382229 | 1.0921572 | 0.0115 | ✱ |  |
| Julia | FR5 DURING PR + MPH | Blank Touch Rate | Session | GLM with zero-inflated beta regression | 0.03683 | -0.2349805 | 0.3086423 | 0.7906 | n.s. |  |
| Julia | FR5 DURING PR + MPH | Blank Touch Rate | Genotype x Session | GLM with zero-inflated beta regression | -0.1145 | -0.4295519 | 0.2005455 | 0.4763 | n.s. |  |
| Julia | PR | Blank Touches | Genotype | GLMM with negative binomial regression | -0.08089 | -0.5843986 | 0.42261124 | 0.753 | n.s. |  |
| Julia | PR | Blank Touches | Session | GLMM with negative binomial regression | -0.07464 | -0.2294956 | 0.08021601 | 0.345 | n.s. |  |
| Julia | PR | Blank Touches | Genotype x Session | GLMM with negative binomial regression | -0.02456 | -0.2421183 | 0.19300315 | 0.825 | n.s. |  |
| Julia | PR + ATO | Blank Touches | Genotype | GLMM with negative binomial regression | 0.21267 | -0.3681472 | 0.7934817 | 0.473 | n.s. |  |
| Julia | PR + ATO | Blank Touches | Drug | GLMM with negative binomial regression | -0.92378 | -1.2566539 | -0.59089697 | 5.35E-08 | @@@ |  |
| Julia | PR + ATO | Blank Touches | Session | GLMM with negative binomial regression | -0.20706 | -0.4414351 | 0.02732117 | 0.0834 | n.s. |  |
| Julia | PR + ATO | Blank Touches | Genotype x Drug | GLMM with negative binomial regression | -0.0639 | -0.5209809 | 0.39317987 | 0.7841 | n.s. |  |
| Julia | PR + ATO | Blank Touches | Genotype x Session | GLMM with negative binomial regression | 0.15914 | -0.1652086 | 0.48348042 | 0.3362 | n.s. |  |
| Julia | PR + ATO | Blank Touches | Drug x Session | GLMM with negative binomial regression | -0.04037 | -0.4029807 | 0.32224596 | 0.8273 | n.s. |  |
| Julia | PR + ATO | Blank Touches | Genotype x Drug x Session | GLMM with negative binomial regression | -0.09628 | -0.6028329 | 0.41026677 | 0.7095 | n.s. |  |
| Julia | PR + MPH | Blank Touches | Genotype | GLMM with negative binomial regression | 0.311552 | -0.1200701 | 0.7431748 | 0.157146 | n.s. |  |
| Julia | PR + MPH | Blank Touches | Drug | GLMM with negative binomial regression | 0.516358 | 0.2516208 | 0.7810948 | 0.000132 | @@@ |  |
| Julia | PR + MPH | Blank Touches | Session | GLMM with negative binomial regression | 0.009435 | -0.1824623 | 0.2013321 | 0.923231 | n.s. |  |
| Julia | PR + MPH | Blank Touches | Genotype x Drug | GLMM with negative binomial regression | -0.210905 | -0.5775698 | 0.1557588 | 0.259585 | n.s. |  |
| Julia | PR + MPH | Blank Touches | Genotype x Session | GLMM with negative binomial regression | 0.053418 | -0.2175926 | 0.3244295 | 0.699256 | n.s. |  |
| Julia | PR + MPH | Blank Touches | Drug x Session | GLMM with negative binomial regression | -0.105151 | -0.383195 | 0.1728927 | 0.458558 | n.s. |  |
| Julia | PR + MPH | Blank Touches | Genotype x Drug x Session | GLMM with negative binomial regression | -0.049863 | -0.4396336 | 0.339908 | 0.802019 | n.s. |  |
| Julia | FR1 | Blank Touches | Genotype | GLMM with negative binomial regression | 0.9674 | -0.2713414 | 2.2061361 | 0.126 | n.s. |  |
| Julia | FR1 | Blank Touches | Session | GLMM with negative binomial regression | -0.3218 | -0.8298354 | 0.1862415 | 0.214 | n.s. |  |
| Julia | FR1 | Blank Touches | Genotype x Session | GLMM with negative binomial regression | 0.1014 | -0.5756733 | 0.7784012 | 0.769 | n.s. |  |
| Julia | FR2 | Blank Touches | Genotype | GLMM with negative binomial regression | 0.15885 | -0.5595974 | 0.8772889 | 0.6648 | n.s. |  |
| Julia | FR2 | Blank Touches | Session | GLMM with negative binomial regression | -0.36392 | -0.7828752 | 0.0550381 | 0.0887 | n.s. |  |
| Julia | FR2 | Blank Touches | Genotype x Session | GLMM with negative binomial regression | -0.04271 | -0.6323978 | 0.5469818 | 0.8871 | n.s. |  |
| Julia | FR3 | Blank Touches | Genotype | GLMM with poisson regression | -0.04854 | -0.3991584 | 0.302082 | 0.786 | n.s. |  |
| Julia | FR5 ALL | Blank Touches | Genotype | GLMM with negative binomial regression | 1.19862 | 0.6718654 | 1.7253755 | 8.20E-06 | ✱✱✱ |  |
| Julia | FR5 ALL | Blank Touches | Session | GLMM with negative binomial regression | -0.45238 | -0.6099003 | -0.2948606 | 1.81E-08 | $$$ |  |
| Julia | FR5 ALL | Blank Touches | Genotype x Session | GLMM with negative binomial regression | 0.30466 | 0.1040203 | 0.5052998 | 0.00292 | ## | **WT - KI Session 1** P=0.1117  **WT - KI Session 2** P=0.0874  **WT - KI Session 3** O=0.5041  **WT - KI Session 4** P=0.0265 (*)  **WT - KI Session 5** P=0.0008 (***)  **WT - KI Session 6** P=0.0233 (*)  **WT - KI Session 7** P=0.0014 (**)  **WT - KI Session 8** P=0.0409 (*)  **WT - KI Session 9** P=0.0075 (**)  **WT - KI Session 10** P=0.0002 (***)  **WT - KI Session 11** P=0.0693  **WT - KI Session 12** P=0.0007 (***)  **WT - KI Session 13** P<.0001 (***)  **WT - KI Session 14** P<.0001 (***)  **WT - KI Session 15** P=0.003 (**)  **WT - KI Session 16** P=0.0838  WT - KI Session 17 P=0.0088 (**) |
| Julia | FR5 BEFORE PR + ATO | Blank Touches | Genotype | GLMM with negative binomial regression | 0.906 | 0.068054689 | 1.7440371 | 0.0341 | ✱ |  |
| Julia | FR5 BEFORE PR + ATO | Blank Touches | Session | GLMM with negative binomial regression | -0.711 | -0.989860194 | -0.432192 | 5.80E-07 | $$$ |  |
| Julia | FR5 BEFORE PR + ATO | Blank Touches | Genotype x Session | GLMM with negative binomial regression | 0.2194 | -0.132446475 | 0.5713266 | 0.2216 | n.s. |  |
| Julia | FR5 DURING PR + ATO | Blank Touches | Genotype | GLMM with negative binomial regression | 1.3995 | 0.5902547 | 2.20867378 | 0.0007 | ✱✱✱ |  |
| Julia | FR5 DURING PR + ATO | Blank Touches | Session | GLMM with negative binomial regression | -0.3321 | -0.6424267 | -0.02169409 | 0.036 | $ |  |
| Julia | FR5 DURING PR + ATO | Blank Touches | Genotype x Session | GLMM with negative binomial regression | 0.243 | -0.1424618 | 0.62838729 | 0.2166 | n.s. |  |
| Julia | FR5 DURING PR + MPH | Blank Touches | Genotype | GLMM with negative binomial regression | 1.4847 | 8.54E-01 | 2.1158693 | 4.02E-06 | ✱✱✱ |  |
| Julia | FR5 DURING PR + MPH | Blank Touches | Session | GLMM with negative binomial regression | 0.6703 | 2.09E-01 | 1.1313898 | 0.00438 | $$ |  |
| Julia | FR5 DURING PR + MPH | Blank Touches | Genotype x Session | GLMM with negative binomial regression | -0.7252 | -1.29E+00 | -0.1553723 | 0.01262 | # | **WT - KI Session 1** P=0.0006 (***)  **WT - KI Session 2** P=0.041 (*)  **WT - KI Session 3** P=0.0064 (**)  **WT - KI Session 4** P=0.2762  **WT - KI Session 5** P=0.4089 |
| Julia | PR | Breakpoint | Genotype | GLMM with negative binomial regression | -0.23469 | -0.483695 | 0.01430541 | 0.064695 | n.s. |  |
| Julia | PR | Breakpoint | Session | GLMM with negative binomial regression | -0.08258 | -0.1289458 | -0.03621466 | 0.000482 | $$$ |  |
| Julia | PR | Breakpoint | Genotype x Session | GLMM with negative binomial regression | 0.04156 | -0.02553004 | 0.10865208 | 0.224693 | n.s. |  |
| Julia | PR + ATO | Breakpoint | Genotype | GLMM with negative binomial regression | -0.08761 | -0.26011635 | 0.08488972 | 0.3195 | n.s. |  |
| Julia | PR + ATO | Breakpoint | Drug | GLMM with negative binomial regression | -0.27936 | -0.37614853 | -0.18256848 | 1.54E-08 | @@@ | **WT Saline - ATO**  P<.0001 (***)  **KI Saline - ATO** P<.0001 (***) |
| Julia | PR + ATO | Breakpoint | Session | GLMM with negative binomial regression | -0.03053 | -0.09892624 | 0.03787341 | 0.3817 | n.s. |  |
| Julia | PR + ATO | Breakpoint | Genotype x Drug | GLMM with negative binomial regression | -0.15078 | -0.28920179 | -0.01235082 | 0.0328 | % | **WT - KI Saline** P=0.3018 **WT - KI ATO** P=0.007 (**) |
| Julia | PR + ATO | Breakpoint | Genotype x Session | GLMM with negative binomial regression | 0.02749 | -0.06980455 | 0.12478581 | 0.5797 | n.s. |  |
| Julia | PR + ATO | Breakpoint | Drug x Session | GLMM with negative binomial regression | -0.01889 | -0.12644988 | 0.08866885 | 0.7307 | n.s. |  |
| Julia | PR + ATO | Breakpoint | Genotype x Drug x Session | GLMM with negative binomial regression | -0.04519 | -0.19901301 | 0.10864186 | 0.5648 | n.s. |  |
| Julia | PR + MPH | Breakpoint | Genotype | GLMM with negative binomial regression | -0.180942 | -0.38170972 | 0.0198258 | 0.0773 | n.s. |  |
| Julia | PR + MPH | Breakpoint | Drug | GLMM with negative binomial regression | 0.172939 | 0.09298086 | 0.25289798 | 2.24E-05 | @@@ | **WT Saline - MPH** P=0.0001 (***)  **KI Saline - MPH** P=0.0003 (***) |
| Julia | PR + MPH | Breakpoint | Session | GLMM with negative binomial regression | 0.005915 | -0.05469061 | 0.06652133 | 0.8483 | n.s. |  |
| Julia | PR + MPH | Breakpoint | Genotype x Drug | GLMM with negative binomial regression | -0.002531 | -0.11708198 | 0.1120208 | 0.9655 | n.s. |  |
| Julia | PR + MPH | Breakpoint | Genotype x Session | GLMM with negative binomial regression | 0.041803 | -0.04502185 | 0.12862712 | 0.3454 | n.s. |  |
| Julia | PR + MPH | Breakpoint | Drug x Session | GLMM with negative binomial regression | 0.0062 | -0.08043135 | 0.09283191 | 0.8884 | n.s. |  |
| Julia | PR + MPH | Breakpoint | Genotype x Drug x Session | GLMM with negative binomial regression | -0.015085 | -0.13922688 | 0.10905778 | 0.8118 | n.s. |  |
| Julia | PR | Discrimination Ratio | Genotype | GLMM with zero-inflated beta regression | 0.30692 | -0.08040947 | 0.69424441 | 0.12 | n.s. |  |
| Julia | PR | Discrimination Ratio | Session | GLMM with zero-inflated beta regression | 0.07333 | -0.04560288 | 0.19226704 | 0.227 | n.s. |  |
| Julia | PR | Discrimination Ratio | Genotype x Session | GLMM with zero-inflated beta regression | -0.07072 | -0.22880412 | 0.08737199 | 0.381 | n.s. |  |
| Julia | PR + ATO | Discrimination Ratio | Genotype | GLMM with zero-inflated beta regression | 0.32032 | -0.06259121 | 0.70324027 | 0.10109 | n.s. |  |
| Julia | PR + ATO | Discrimination Ratio | Drug | GLMM with zero-inflated beta regression | -0.41504 | -0.68104091 | -0.14903639 | 0.00223 | @@ |  |
| Julia | PR + ATO | Discrimination Ratio | Session | GLMM with zero-inflated beta regression | -0.07935 | -0.25550213 | 0.09679297 | 0.37726 | n.s. |  |
| Julia | PR + ATO | Discrimination Ratio | Genotype x Drug | GLMM with zero-inflated beta regression | 0.04666 | -0.30031052 | 0.39364009 | 0.79209 | n.s. |  |
| Julia | PR + ATO | Discrimination Ratio | Genotype x Session | GLMM with zero-inflated beta regression | 0.05623 | -0.17837325 | 0.29082776 | 0.63853 | n.s. |  |
| Julia | PR + ATO | Discrimination Ratio | Drug x Session | GLMM with zero-inflated beta regression | -0.06071 | -0.35062378 | 0.22920886 | 0.68151 | n.s. |  |
| Julia | PR + ATO | Discrimination Ratio | Genotype x Drug x Session | GLMM with zero-inflated beta regression | -0.11876 | -0.50406192 | 0.26654712 | 0.54578 | n.s. |  |
| Julia | PR + MPH | Discrimination Ratio | Genotype | GLMM with zero-inflated beta regression | 0.49343 | 0.09990244 | 0.8869597 | 0.014 | ✱ |  |
| Julia | PR + MPH | Discrimination Ratio | Drug | GLMM with zero-inflated beta regression | 0.18183 | -0.04641487 | 0.4100678 | 0.118 | n.s. |  |
| Julia | PR + MPH | Discrimination Ratio | Session | GLMM with zero-inflated beta regression | -0.02927 | -0.20624526 | 0.1477123 | 0.746 | n.s. |  |
| Julia | PR + MPH | Discrimination Ratio | Genotype x Drug | GLMM with zero-inflated beta regression | -0.11128 | -0.40864766 | 0.1860786 | 0.463 | n.s. |  |
| Julia | PR + MPH | Discrimination Ratio | Genotype x Session | GLMM with zero-inflated beta regression | 0.03301 | -0.19321312 | 0.2592339 | 0.775 | n.s. |  |
| Julia | PR + MPH | Discrimination Ratio | Drug x Session | GLMM with zero-inflated beta regression | -0.09483 | -0.33974351 | 0.1500821 | 0.448 | n.s. |  |
| Julia | PR + MPH | Discrimination Ratio | Genotype x Drug x Session | GLMM with zero-inflated beta regression | -0.05305 | -0.37246609 | 0.2663756 | 0.745 | n.s. |  |
| Julia | FR1 | Discrimination Ratio | Genotype | GLMM with zero-inflated beta regression | -0.06611 | -0.6205351 | 0.48831379 | 0.815 | n.s. |  |
| Julia | FR1 | Discrimination Ratio | Session | GLMM with zero-inflated beta regression | -0.24663 | -0.6853746 | 0.1921073 | 0.271 | n.s. |  |
| Julia | FR1 | Discrimination Ratio | Genotype x Session | GLMM with zero-inflated beta regression | 0.13066 | -0.4284448 | 0.6897665 | 0.647 | n.s. |  |
| Julia | FR2 | Discrimination Ratio | Genotype | GLMM with zero-inflated beta regression | -0.03254 | -0.4641492 | 0.3990773 | 0.883 | n.s. |  |
| Julia | FR2 | Discrimination Ratio | Session | GLMM with zero-inflated beta regression | -0.13744 | -0.4610102 | 0.1861226 | 0.405 | n.s. |  |
| Julia | FR2 | Discrimination Ratio | Genotype x Session | GLMM with zero-inflated beta regression | -0.01878 | -0.453911 | 0.4163462 | 0.933 | n.s. |  |
| Julia | FR3 | Discrimination Ratio | Genotype | GLM with zero-inflated beta regression | -0.06095 | -0.6208797 | 0.4989735 | 0.831 | n.s. |  |
| Julia | FR5 ALL | Discrimination Ratio | Genotype | GLMM with zero-inflated beta regression | 0.613324 | 0.2166108 | 1.01003807 | 0.00244 | ✱✱ |  |
| Julia | FR5 ALL | Discrimination Ratio | Session | GLMM with zero-inflated beta regression | -0.199351 | -0.3401295 | -0.05857178 | 0.00551 | $$ |  |
| Julia | FR5 ALL | Discrimination Ratio | Genotype x Session | GLMM with zero-inflated beta regression | 0.005403 | -0.1548441 | 0.16565017 | 0.94731 | n.s. |  |
| Julia | FR5 BEFORE PR + ATO | Discrimination Ratio | Genotype | GLMM with zero-inflated beta regression | 0.69057 | 0.2214301 | 1.1597062 | 0.00391 | ✱✱ |  |
| Julia | FR5 BEFORE PR + ATO | Discrimination Ratio | Session | GLMM with zero-inflated beta regression | -0.33364 | -0.47499381 | -0.1922847 | 3.73E-06 | $$$ |  |
| Julia | FR5 BEFORE PR + ATO | Discrimination Ratio | Genotype x Session | GLMM with zero-inflated beta regression | 0.22477 | 0.05344257 | 0.3960955 | 0.01013 | # | **WT - KI Session 1** P=0.2742  **WT - KI Session 2** P=0.0545  **WT - KI Session 3** P=0.3438  **WT - KI Session 4** P=0.027 (*)  **WT - KI Session 5** P=0.0054 (**)  **WT - KI Session 6** P=0.0085 (**)  **WT - KI Session 7** P=0.0049 (**) |
| Julia | FR5 DURING PR + ATO | Discrimination Ratio | Genotype | GLMM with zero-inflated beta regression | 0.57951 | -0.000929707 | 1.1599418 | 0.0504 | n.s. |  |
| Julia | FR5 DURING PR + ATO | Discrimination Ratio | Session | GLMM with zero-inflated beta regression | -0.08168 | -0.323802891 | 0.1604425 | 0.5085 | n.s. |  |
| Julia | FR5 DURING PR + ATO | Discrimination Ratio | Genotype x Session | GLMM with zero-inflated beta regression | -0.03391 | -0.300646724 | 0.2328357 | 0.8033 | n.s. |  |
| Julia | FR5 DURING PR + MPH | Discrimination Ratio | Genotype | GLMM with zero-inflated beta regression | 0.73367 | 0.2450402 | 1.2222999 | 0.00325 | ✱✱ |  |
| Julia | FR5 DURING PR + MPH | Discrimination Ratio | Session | GLMM with zero-inflated beta regression | 0.09339 | -0.1953329 | 0.3821034 | 0.52612 | n.s. |  |
| Julia | FR5 DURING PR + MPH | Discrimination Ratio | Genotype x Session | GLMM with zero-inflated beta regression | -0.14217 | -0.4770581 | 0.1927248 | 0.40539 | n.s. |  |
| Julia | PR | Magazine Entry Rate | Genotype | GLMM with beta regression | 0.27516 | 0.0117755 | 0.53853718 | 0.0406 | ✱ |  |
| Julia | PR | Magazine Entry Rate | Session | GLMM with beta regression | -0.15575 | -0.24040273 | -0.07109178 | 0.000311 | $$$ |  |
| Julia | PR | Magazine Entry Rate | Genotype x Session | GLMM with beta regression | 0.13635 | 0.02311447 | 0.24958858 | 0.018273 | # | **WT - KI Session 1** P=0.2971  **WT - KI Session 2** P=0.4888  **WT - KI Session 3** P=0.0503  **WT - KI Session 4** P=0.0031 (**) |
| Julia | PR + ATO | Magazine Entry Rate | Genotype | GLMM with beta regression | 0.13194 | -0.12655189 | 0.3904234 | 0.31712 | n.s. |  |
| Julia | PR + ATO | Magazine Entry Rate | Drug | GLMM with beta regression | -0.32814 | -0.49973554 | -0.15654504 | 0.000178 | @@@ |  |
| Julia | PR + ATO | Magazine Entry Rate | Session | GLMM with beta regression | -0.01566 | -0.13194736 | 0.10062804 | 0.79183 | n.s. |  |
| Julia | PR + ATO | Magazine Entry Rate | Genotype x Drug | GLMM with beta regression | 0.17869 | -0.04811363 | 0.40550312 | 0.122542 | n.s. |  |
| Julia | PR + ATO | Magazine Entry Rate | Genotype x Session | GLMM with beta regression | -0.03927 | -0.19832053 | 0.11977324 | 0.628403 | n.s. |  |
| Julia | PR + ATO | Magazine Entry Rate | Drug x Session | GLMM with beta regression | -0.09658 | -0.28324987 | 0.09008097 | 0.310523 | n.s. |  |
| Julia | PR + ATO | Magazine Entry Rate | Genotype x Drug x Session | GLMM with beta regression | 0.09038 | -0.15962515 | 0.3403779 | 0.478614 | n.s. |  |
| Julia | PR + MPH | Magazine Entry Rate | Genotype | GLMM with beta regression | 0.34992 | 0.0906818 | 0.60916678 | 0.00816 | ✱✱ |  |
| Julia | PR + MPH | Magazine Entry Rate | Drug | GLMM with beta regression | 0.14808 | -0.03449304 | 0.33064505 | 0.11191 | n.s. |  |
| Julia | PR + MPH | Magazine Entry Rate | Session | GLMM with beta regression | -0.04648 | -0.18565229 | 0.0926874 | 0.51271 | n.s. |  |
| Julia | PR + MPH | Magazine Entry Rate | Genotype x Drug | GLMM with beta regression | -0.14054 | -0.38093902 | 0.09985553 | 0.25186 | n.s. |  |
| Julia | PR + MPH | Magazine Entry Rate | Genotype x Session | GLMM with beta regression | 0.02959 | -0.15286385 | 0.21205024 | 0.75057 | n.s. |  |
| Julia | PR + MPH | Magazine Entry Rate | Drug x Session | GLMM with beta regression | -0.03385 | -0.22886573 | 0.16117064 | 0.73373 | n.s. |  |
| Julia | PR + MPH | Magazine Entry Rate | Genotype x Drug x Session | GLMM with beta regression | -0.11714 | -0.37357596 | 0.13929087 | 0.37061 | n.s. |  |
| Julia | FR1 | Magazine Entry Rate | Genotype | GLMM with beta regression | -0.10767 | -0.34444711 | 0.1291008 | 0.373 | n.s. |  |
| Julia | FR1 | Magazine Entry Rate | Session | GLMM with beta regression | 0.04099 | -0.06437928 | 0.1463643 | 0.446 | n.s. |  |
| Julia | FR1 | Magazine Entry Rate | Genotype x Session | GLMM with beta regression | 0.04248 | -0.10726404 | 0.1922228 | 0.578 | n.s. |  |
| Julia | FR2 | Magazine Entry Rate | Genotype | GLMM with beta regression | -0.12862 | -0.29606141 | 0.03881318 | 0.132 | n.s. |  |
| Julia | FR2 | Magazine Entry Rate | Session | GLMM with beta regression | 0.03285 | -0.01224092 | 0.0779416 | 0.153 | n.s. |  |
| Julia | FR2 | Magazine Entry Rate | Genotype x Session | GLMM with beta regression | -0.02587 | -0.08945482 | 0.03771775 | 0.425 | n.s. |  |
| Julia | FR3 | Magazine Entry Rate | Genotype | GLM with beta regression | -0.09326 | -0.2523765 | 0.06586196 | 0.251 | n.s. |  |
| Julia | FR5 ALL | Magazine Entry Rate | Genotype | GLMM with beta regression | -0.102574 | -0.200209874 | -0.004938328 | 0.03948 | ✱ |  |
| Julia | FR5 ALL | Magazine Entry Rate | Session | GLMM with beta regression | 0.013678 | -0.005049125 | 0.032405265 | 0.15228 | n.s. |  |
| Julia | FR5 ALL | Magazine Entry Rate | Genotype x Session | GLMM with beta regression | 0.036176 | 0.009751829 | 0.062601071 | 0.00729 | ## | **WT - KI Session 1** P=0.0041 (**)  **WT - KI Session 2** P=0.0036 (**)  **WT - KI Session 3** P=0.048 (*)  **WT - KI Session 4** P=0.0076 (**)  **WT - KI Session 5** P=0.231  **WT - KI Session 6** P=0.3031  **WT - KI Session 7** P=0.1242  **WT - KI Session 8** P=0.2128  **WT - KI Session 9** P=0.0322 (*)  **WT - KI Session 10** P=0.3655  **WT - KI Session 11** P=0.9094  **WT - KI Session 12** P=0.4139  **WT - KI Session 13** P=0.0549  **WT - KI Session 14** P=0.1643  **WT - KI Session 15** P=0.9578  **WT - KI Session 16** P=0.5553  **WT - KI Session 17** P=0.4205 |
| Julia | FR5 BEFORE PR + ATO | Magazine Entry Rate | Genotype | GLMM with beta regression | -0.148545 | -0.266802028 | -0.03028764 | 0.0138 | ✱ |  |
| Julia | FR5 BEFORE PR + ATO | Magazine Entry Rate | Session | GLMM with beta regression | -0.003931 | -0.027940246 | 0.0200783 | 0.7483 | n.s. |  |
| Julia | FR5 BEFORE PR + ATO | Magazine Entry Rate | Genotype x Session | GLMM with beta regression | 0.044194 | 0.009661498 | 0.07872649 | 0.0121 | # | **WT - KI Session 1** P=0.0039 (**)  **WT - KI Session 2** P=0.0032 (**)  **WT - KI Session 3** P=0.0497 (*)  **WT - KI Session 4** P=0.008 (**)  **WT - KI Session 5** P=0.2351  **WT - KI Session 6** P=0.294  **WT - KI Session 7** P=0.1243 |
| Julia | FR5 DURING PR + ATO | Magazine Entry Rate | Genotype | GLMM with beta regression | -0.07244 | -0.16976415 | 0.024885 | 0.1446 | n.s. |  |
| Julia | FR5 DURING PR + ATO | Magazine Entry Rate | Session | GLMM with beta regression | 0.03408 | 0.00106029 | 0.06709892 | 0.0431 | $ |  |
| Julia | FR5 DURING PR + ATO | Magazine Entry Rate | Genotype x Session | GLMM with beta regression | 0.02928 | -0.01728772 | 0.07584986 | 0.2178 | n.s. |  |
| Julia | FR5 DURING PR + MPH | Magazine Entry Rate | Genotype | GLMM with beta regression | -0.06987 | -0.19239975 | 0.052669088 | 0.264 | n.s. |  |
| Julia | FR5 DURING PR + MPH | Magazine Entry Rate | Session | GLMM with beta regression | -0.02161 | -0.05152593 | 0.008305134 | 0.157 | n.s. |  |
| Julia | FR5 DURING PR + MPH | Magazine Entry Rate | Genotype x Session | GLMM with beta regression | 0.02678 | -0.01507435 | 0.068641775 | 0.21 | n.s. |  |
| Julia | PR | Magazine Entries | Genotype | GLMM with negative binomial regression | -0.08089 | -0.5843986 | 0.42261124 | 0.753 | n.s. |  |
| Julia | PR | Magazine Entries | Session | GLMM with negative binomial regression | -0.07464 | -0.2294956 | 0.08021601 | 0.345 | n.s. |  |
| Julia | PR | Magazine Entries | Genotype x Session | GLMM with negative binomial regression | -0.02456 | -0.2421183 | 0.19300315 | 0.825 | n.s. |  |
| Julia | PR + ATO | Magazine Entries | Genotype | GLMM with negative binomial regression | 0.21267 | -0.3681472 | 0.7934817 | 0.473 | n.s. |  |
| Julia | PR + ATO | Magazine Entries | Drug | GLMM with negative binomial regression | -0.92378 | -1.2566539 | -0.59089697 | 5.35E-08 | @@@ |  |
| Julia | PR + ATO | Magazine Entries | Session | GLMM with negative binomial regression | -0.20706 | -0.4414351 | 0.02732117 | 0.0834 | n.s. |  |
| Julia | PR + ATO | Magazine Entries | Genotype x Drug | GLMM with negative binomial regression | -0.0639 | -0.5209809 | 0.39317987 | 0.7841 | n.s. |  |
| Julia | PR + ATO | Magazine Entries | Genotype x Session | GLMM with negative binomial regression | 0.15914 | -0.1652086 | 0.48348042 | 0.3362 | n.s. |  |
| Julia | PR + ATO | Magazine Entries | Drug x Session | GLMM with negative binomial regression | -0.04037 | -0.4029807 | 0.32224596 | 0.8273 | n.s. |  |
| Julia | PR + ATO | Magazine Entries | Genotype x Drug x Session | GLMM with negative binomial regression | -0.09628 | -0.6028329 | 0.41026677 | 0.7095 | n.s. |  |
| Julia | PR + MPH | Magazine Entries | Genotype | GLMM with negative binomial regression | 0.311552 | -0.1200701 | 0.7431748 | 0.157146 | n.s. |  |
| Julia | PR + MPH | Magazine Entries | Drug | GLMM with negative binomial regression | 0.516358 | 0.2516208 | 0.7810948 | 0.000132 | @@@ |  |
| Julia | PR + MPH | Magazine Entries | Session | GLMM with negative binomial regression | 0.009435 | -0.1824623 | 0.2013321 | 0.923231 | n.s. |  |
| Julia | PR + MPH | Magazine Entries | Genotype x Drug | GLMM with negative binomial regression | -0.210905 | -0.5775698 | 0.1557588 | 0.259585 | n.s. |  |
| Julia | PR + MPH | Magazine Entries | Genotype x Session | GLMM with negative binomial regression | 0.053418 | -0.2175926 | 0.3244295 | 0.699256 | n.s. |  |
| Julia | PR + MPH | Magazine Entries | Drug x Session | GLMM with negative binomial regression | -0.105151 | -0.383195 | 0.1728927 | 0.458558 | n.s. |  |
| Julia | PR + MPH | Magazine Entries | Genotype x Drug x Session | GLMM with negative binomial regression | -0.049863 | -0.4396336 | 0.6749238 | 0.802019 | n.s. |  |
| Julia | FR1 | Magazine Entries | Genotype | GLMM with negative binomial regression | 0.9674 | -0.2713414 | 2.2061361 | 0.126 | n.s. |  |
| Julia | FR1 | Magazine Entries | Session | GLMM with negative binomial regression | -0.3218 | -0.8298354 | 0.1862415 | 0.214 | n.s. |  |
| Julia | FR1 | Magazine Entries | Genotype x Session | GLMM with negative binomial regression | 0.1014 | -0.5756733 | 0.7784012 | 0.769 | n.s. |  |
| Julia | FR2 | Magazine Entries | Genotype | GLMM with negative binomial regression | 0.15885 | -0.5595974 | 0.8772889 | 0.6648 | n.s. |  |
| Julia | FR2 | Magazine Entries | Session | GLMM with negative binomial regression | -0.36392 | -0.7828752 | 0.0550381 | 0.0887 | n.s. |  |
| Julia | FR2 | Magazine Entries | Genotype x Session | GLMM with negative binomial regression | -0.04271 | -0.6323978 | 0.5469818 | 0.8871 | n.s. |  |
| Julia | FR3 | Magazine Entries | Genotype | GLM with negative binomial regression | -0.04854 | -0.9487733 | 0.8516969 | 0.916 | n.s. |  |
| Julia | FR5 ALL | Magazine Entries | Genotype | GLMM with negative binomial regression | 1.19862 | 0.6718654 | 1.7253755 | 8.20E-06 | ✱✱✱ |  |
| Julia | FR5 ALL | Magazine Entries | Session | GLMM with negative binomial regression | -0.45238 | -0.6099003 | -0.2948606 | 1.81E-08 | $$$ |  |
| Julia | FR5 ALL | Magazine Entries | Genotype x Session | GLMM with negative binomial regression | 0.30466 | 0.1040203 | 0.5052998 | 0.00292 | ## | **WT - KI Session 1** P=0.1117  **WT - KI Session 2** P=0.0874  **WT - KI Session 3** P=0.5041  **WT - KI Session 4** P=0.0265 (*)  **WT - KI Session 5** P=0.0008 (***)  **WT - KI Session 6** P=0.0233 (*)  **WT - KI Session 7** P=0.0014 (**)  **WT - KI Session 8** P=0.0409 (*)  **WT - KI Session 9** P=0.0075 (**)  **WT - KI Session 10** P=0.0002 (***)  **WT - KI Session 11** P=0.0693  **WT - KI Session 12** P=00007 (***)  **WT - KI Session 13** P<.0001 (***)  **WT - KI Session 14** P<.0001 (***)  **WT - KI Session 15** P=0.003 (**)  **WT - KI Session 16** P=0.0838  **WT - KI Session 17** P=0.0088 (**) |
| Julia | FR5 BEFORE PR + ATO | Magazine Entries | Genotype | GLMM with negative binomial regression | 1.05284 | 0.41183473 | 1.6938437 | 0.001285 | ✱✱ |  |
| Julia | FR5 BEFORE PR + ATO | Magazine Entries | Session | GLMM with negative binomial regression | -0.57658 | -0.75487853 | -0.3982839 | 2.33E-10 | $$$ |  |
| Julia | FR5 BEFORE PR + ATO | Magazine Entries | Genotype x Session | GLMM with negative binomial regression | 0.29325 | 0.06394213 | 0.5225525 | 0.012193 | # | **WT - KI Session 1** P=0.0732  **WT - KI Session 2** P=0.0537  **WT - KI Session 3** P=0.2772  **WT - KI Session 4** P=0.0146 (*)  **WT - KI Session 5** P=0.0002 (***)  **WT - KI Session 6** P=0.0109 (*)  **WT - KI Session 7** P=0.0007 (***) |
| Julia | FR5 DURING PR + ATO | Magazine Entries | Genotype | GLMM with negative binomial regression | 1.3995 | 0.5902547 | 2.20867378 | 0.0007 | ✱✱✱ |  |
| Julia | FR5 DURING PR + ATO | Magazine Entries | Session | GLMM with negative binomial regression | -0.3321 | -0.6424267 | -0.02169409 | 0.036 | $ |  |
| Julia | FR5 DURING PR + ATO | Magazine Entries | Genotype x Session | GLMM with negative binomial regression | 0.243 | -0.1424618 | 0.62838729 | 0.2166 | n.s. |  |
| Julia | FR5 DURING PR + MPH | Magazine Entries | Genotype | GLMM with negative binomial regression | 1.9518 | 1.2627624 | 2.64089825 | 2.83E-08 | ✱✱✱ |  |
| Julia | FR5 DURING PR + MPH | Magazine Entries | Session | GLMM with negative binomial regression | 0.5142 | 0.1409098 | 0.88754288 | 0.00694 | $$ |  |
| Julia | FR5 DURING PR + MPH | Magazine Entries | Genotype x Session | GLMM with negative binomial regression | -0.5367 | -0.9815728 | -0.09187523 | 0.01804 | # | **WT - KI Session 1** P<0.0001 (***)  **WT - KI Session 2** P=0.0001 (***)  **WT - KI Session 3** P=0.0019 (**)  **WT - KI Session 4** P=0.0501  **WT - KI Session 5** P=0.005 (**) |
| Julia | PR | Reward Collection Latency | Genotype | GLMM with log-adjusted inverse gaussian regression | -0.15082 | -0.259496372 | -0.04214741 | 0.00653 | ✱✱ |  |
| Julia | PR | Reward Collection Latency | Session | GLMM with log-adjusted inverse gaussian regression | 0.03962 | 0.004094916 | 0.07515056 | 2.88E-02 | $ |  |
| Julia | PR | Reward Collection Latency | Genotype x Session | GLMM with log-adjusted inverse gaussian regression | -0.02046 | -0.069965706 | 0.02905537 | 0.41808 | n.s. |  |
| Julia | PR + ATO | Reward Collection Latency | Genotype | GLMM with log-adjusted inverse gaussian regression | -0.10729 | -0.188538856 | -0.026042083 | 0.00965 | ✱✱ |  |
| Julia | PR + ATO | Reward Collection Latency | Drug | GLMM with log-adjusted inverse gaussian regression | 0.05069 | 0.019642221 | 0.081738055 | 1.37E-03 | @@ |  |
| Julia | PR + ATO | Reward Collection Latency | Session | GLMM with log-adjusted inverse gaussian regression | 0.0376 | -0.007588966 | 0.082788489 | 0.10293 | n.s. |  |
| Julia | PR + ATO | Reward Collection Latency | Genotype x Drug | GLMM with log-adjusted inverse gaussian regression | -0.04459 | -0.087487441 | -0.001702165 | 4.16E-02 | % | **WT - KI Saline** P=0.0451 (*)  **WT - KI ATO** P=0.0001 (***) |
| Julia | PR + ATO | Reward Collection Latency | Genotype x Session | GLMM with log-adjusted inverse gaussian regression | -0.04561 | -0.108179628 | 0.016956743 | 0.15306 | n.s. |  |
| Julia | PR + ATO | Reward Collection Latency | Drug x Session | GLMM with log-adjusted inverse gaussian regression | -0.01299 | -0.045960593 | 0.019990581 | 0.44024 | n.s. |  |
| Julia | PR + ATO | Reward Collection Latency | Genotype x Drug x Session | GLMM with log-adjusted inverse gaussian regression | 0.0641 | 0.017607347 | 0.11059554 | 0.00689 | ^^ | Three-way interactions are difficult to interpret. |
| Julia | PR + MPH | Reward Collection Latency | Genotype | GLMM with log-adjusted inverse gaussian regression | -0.099761 | -0.190414179 | -0.009108423 | 0.03101 | ✱ |  |
| Julia | PR + MPH | Reward Collection Latency | Drug | GLMM with log-adjusted inverse gaussian regression | -0.096805 | -0.119280726 | -0.074330272 | 2.00E-16 | @@@ |  |
| Julia | PR + MPH | Reward Collection Latency | Session | GLMM with log-adjusted inverse gaussian regression | 0.023314 | -0.001515745 | 0.04814318 | 0.06572 | n.s. |  |
| Julia | PR + MPH | Reward Collection Latency | Genotype x Drug | GLMM with log-adjusted inverse gaussian regression | 0.049438 | 0.017395805 | 0.081480934 | 0.00249 | %% | **WT - KI Saline** P=0.0465 (*)  **WT - KI MPH** P=0.2442 |
| Julia | PR + MPH | Reward Collection Latency | Genotype x Session | GLMM with log-adjusted inverse gaussian regression | -0.006754 | -0.041479583 | 0.027972192 | 0.70307 | n.s. |  |
| Julia | PR + MPH | Reward Collection Latency | Drug x Session | GLMM with log-adjusted inverse gaussian regression | -0.017114 | -0.040338914 | 0.006110546 | 0.14866 | n.s. |  |
| Julia | PR + MPH | Reward Collection Latency | Genotype x Drug x Session | GLMM with log-adjusted inverse gaussian regression | 0.015346 | -0.017468301 | 0.048161122 | 0.35935 | n.s. |  |
| Julia | FR1 | Reward Collection Latency | Genotype | GLMM with log-adjusted inverse gaussian regression | -0.082117 | -0.15836048 | -0.005872577 | 0.0348 | ✱ |  |
| Julia | FR1 | Reward Collection Latency | Session | GLMM with log-adjusted inverse gaussian regression | 0.007262 | -0.02820834 | 0.042732575 | 0.6882 | n.s. |  |
| Julia | FR1 | Reward Collection Latency | Genotype x Session | GLMM with log-adjusted inverse gaussian regression | 0.003395 | -0.04645818 | 0.053249058 | 0.8938 | n.s. |  |
| Julia | FR2 | Reward Collection Latency | Genotype | GLMM with log-adjusted inverse gaussian regression | -0.092883 | -0.15957644 | -0.02618925 | 0.00634 | ✱✱ |  |
| Julia | FR2 | Reward Collection Latency | Session | GLMM with log-adjusted inverse gaussian regression | -0.009271 | -0.03073554 | 0.01219383 | 0.39726 | n.s. |  |
| Julia | FR2 | Reward Collection Latency | Genotype x Session | GLMM with log-adjusted inverse gaussian regression | -0.00111 | -0.03056674 | 0.02834736 | 0.94114 | n.s. |  |
| Julia | FR3 | Reward Collection Latency | Genotype | GLMM with log-adjusted inverse gaussian regression | -0.09811 | -0.1591904 | -0.03703085 | 0.00164 | ✱✱ |  |
| Julia | FR5 ALL | Reward Collection Latency | Genotype | GLMM with log-adjusted inverse gaussian regression | -0.13086 | -0.19989306 | -0.061830564 | 0.000203 | ✱✱✱ |  |
| Julia | FR5 ALL | Reward Collection Latency | Session | GLMM with log-adjusted inverse gaussian regression | 0.06382 | 0.04037289 | 0.087262908 | 9.55E-08 | $$$ |  |
| Julia | FR5 ALL | Reward Collection Latency | Genotype x Session | GLMM with log-adjusted inverse gaussian regression | -0.041 | -0.07431733 | -0.007681793 | 0.015872 | # |  |
| Julia | FR5 BEFORE PR + ATO | Reward Collection Latency | Genotype | GLMM with log-adjusted inverse gaussian regression | -0.096467 | -0.15493785 | -0.037996027 | 0.00122 | ✱✱ |  |
| Julia | FR5 BEFORE PR + ATO | Reward Collection Latency | Session | GLMM with log-adjusted inverse gaussian regression | -0.019493 | -0.03654283 | -0.002442434 | 0.02504 | $ |  |
| Julia | FR5 BEFORE PR + ATO | Reward Collection Latency | Genotype x Session | GLMM with log-adjusted inverse gaussian regression | -0.007658 | -0.03163585 | 0.016320561 | 0.53136 | n.s. |  |
| Julia | FR5 DURING PR + ATO | Reward Collection Latency | Genotype | GLMM with log-adjusted inverse gaussian regression | -0.11738 | -0.232518305 | -0.002238463 | 0.0457 | ✱ |  |
| Julia | FR5 DURING PR + ATO | Reward Collection Latency | Session | GLMM with log-adjusted inverse gaussian regression | 0.02313 | 0.001703395 | 0.044552797 | 0.0344 | $ |  |
| Julia | FR5 DURING PR + ATO | Reward Collection Latency | Genotype x Session | GLMM with log-adjusted inverse gaussian regression | -0.03025 | -0.060381929 | -0.000125633 | 0.0491 | # | **WT - KI Session 1** P=0.0232 (*)  **WT - KI Session 2** P=0.1106  **WT - KI Session 3** P=0.4733  **WT - KI Session 4** 0.0036 (**)  **WT - KI Session 5** P=0.0242 (*) |
| Julia | FR5 DURING PR + MPH | Reward Collection Latency | Genotype | GLMM with log-adjusted inverse gaussian regression | -0.18733 | -0.28690354 | -0.08776027 | 0.000227 | ✱✱✱ |  |
| Julia | FR5 DURING PR + MPH | Reward Collection Latency | Session | GLMM with log-adjusted inverse gaussian regression | 0.04051 | 0.008940636 | 0.0720699 | 0.011899 | $ |  |
| Julia | FR5 DURING PR + MPH | Reward Collection Latency | Genotype x Session | GLMM with log-adjusted inverse gaussian regression | -0.02119 | -0.066288576 | 0.0239091 | 0.357107 | n.s. |  |
| Julia | PR | Schedule Length | Genotype | Cox proportional hazards model | 0.7292 | 0.839 | 5.125 | 0.1142 | n.s. |  |
| Julia | PR | Schedule Length | Session | Cox proportional hazards model | 0.421 | 1.0585 | 2.193 | 0.0235 | $ |  |
| Julia | PR | Schedule Length | Genotype x Session | Cox proportional hazards model | -0.4193 | 0.4384 | 0.986 | 0.0425 | # | **WT - KI Session 1** P=0.058  **WT - KI Session 2** P=0.1155  **WT - KI Session 3** P=0.1365  **WT - KI Session 4** P=0.8952 |
| Julia | PR + ATO | Schedule Length | Genotype | Cox proportional hazards model | -0.05597 | 0.4107 | 2.177 | 0.8953 | n.s. |  |
| Julia | PR + ATO | Schedule Length | Drug | Cox proportional hazards model | 0.71825 | 1.1725 | 3.587 | 0.0118 | @ |  |
| Julia | PR + ATO | Schedule Length | Session | Cox proportional hazards model | 0.07435 | 0.7092 | 1.636 | 0.7273 | n.s. |  |
| Julia | PR + ATO | Schedule Length | Genotype x Drug | Cox proportional hazards model | 0.74842 | 1.0093 | 4.426 | 0.0472 | % | **WT - KI Saline** P=0.8699  **WT - KI ATO** P=0.0314 (*) |
| Julia | PR + ATO | Schedule Length | Genotype x Session | Cox proportional hazards model | -0.12604 | 0.4742 | 1.639 | 0.6903 | n.s. |  |
| Julia | PR + ATO | Schedule Length | Drug x Session | Cox proportional hazards model | 0.10994 | 0.6578 | 1.894 | 0.6837 | n.s. |  |
| Julia | PR + ATO | Schedule Length | Genotype x Drug x Session | Cox proportional hazards model | 0.03211 | 0.482 | 2.212 | 0.9342 | n.s. |  |
| Julia | PR + MPH | Schedule Length | Genotype | Cox proportional hazards model | 0.1362 | 0.4495 | 2.922 | 0.7754 | n.s. |  |
| Julia | PR + MPH | Schedule Length | Drug | Cox proportional hazards model | -0.171 | 0.4653 | 1.527 | 0.5726 | n.s. |  |
| Julia | PR + MPH | Schedule Length | Session | Cox proportional hazards model | 0.3352 | 0.9164 | 2.134 | 0.1199 | n.s. |  |
| Julia | PR + MPH | Schedule Length | Genotype x Drug | Cox proportional hazards model | -0.4376 | 0.2891 | 1.441 | 0.2856 | n.s. |  |
| Julia | PR + MPH | Schedule Length | Genotype x Session | Cox proportional hazards model | -0.5892 | 0.3076 | 1.001 | 0.0503 | n.s. |  |
| Julia | PR + MPH | Schedule Length | Drug x Session | Cox proportional hazards model | -0.4422 | 0.3629 | 1.138 | 0.1294 | n.s. |  |
| Julia | PR + MPH | Schedule Length | Genotype x Drug x Session | Cox proportional hazards model | 0.5917 | 0.7463 | 4.376 | 0.1897 | n.s. |  |
| Julia | FR1 | Schedule Length | Genotype | Cox proportional hazards model | -0.399 | 0.3492 | 1.289 | 0.2312 | n.s. |  |
| Julia | FR1 | Schedule Length | Session | Cox proportional hazards model | 0.2733 | 1.0531 | 1.64 | 0.0156 | $ |  |
| Julia | FR1 | Schedule Length | Genotype x Session | Cox proportional hazards model | -0.0015 | 0.7225 | 1.38 | 0.993 | n.s. |  |
| Julia | FR2 | Schedule Length | Genotype | Cox proportional hazards model | -0.78477 | 0.23 | 0.9048 | 0.024684 | ✱ |  |
| Julia | FR2 | Schedule Length | Session | Cox proportional hazards model | 0.16345 | 1.0835 | 1.2798 | 0.000119 | $$$ |  |
| Julia | FR2 | Schedule Length | Genotype x Session | Cox proportional hazards model | 0.11343 | 0.8732 | 1.4368 | 0.371905 | n.s. |  |
| Julia | FR3 | Schedule Length | Genotype | Cox proportional hazards model | -0.6871 | 0.2378 | 1.064 | 0.0722 | n.s. |  |
| Julia | FR5 ALL | Schedule Length | Genotype | Cox proportional hazards model | -0.67629 | 0.326 | 0.7931 | 0.00286 | ✱✱ |  |
| Julia | FR5 ALL | Schedule Length | Session | Cox proportional hazards model | 0.06281 | 0.8865 | 1.279 | 0.50184 | n.s. |  |
| Julia | FR5 ALL | Schedule Length | Genotype x Session | Cox proportional hazards model | 0.10968 | 0.8954 | 1.3907 | 0.32881 | n.s. |  |
| Julia | FR5 BEFORE PR + ATO | Schedule Length | Genotype | Cox proportional hazards model | -0.87581 | 0.2374 | 0.7308 | 0.00227 | ✱✱ |  |
| Julia | FR5 BEFORE PR + ATO | Schedule Length | Session | Cox proportional hazards model | 0.07064 | 0.8772 | 1.313 | 0.49233 | n.s. |  |
| Julia | FR5 BEFORE PR + ATO | Schedule Length | Genotype x Session | Cox proportional hazards model | 0.18353 | 0.9511 | 1.5177 | 0.12365 | n.s. |  |
| Julia | FR5 DURING PR + ATO | Schedule Length | Genotype | Cox proportional hazards model | -0.53429 | 0.352 | 0.9759 | 0.04 | ✱ |  |
| Julia | FR5 DURING PR + ATO | Schedule Length | Session | Cox proportional hazards model | 0.14983 | 0.966 | 1.3969 | 0.111 | n.s. |  |
| Julia | FR5 DURING PR + ATO | Schedule Length | Genotype x Session | Cox proportional hazards model | 0.08576 | 0.8508 | 1.3953 | 0.497 | n.s. |  |
| Julia | FR5 DURING PR + MPH | Schedule Length | Genotype | Cox proportional hazards model | -0.53429 | 0.352 | 0.9759 | 0.04 | ✱ |  |
| Julia | FR5 DURING PR + MPH | Schedule Length | Session | Cox proportional hazards model | 0.14983 | 0.966 | 1.3969 | 0.111 | n.s. |  |
| Julia | FR5 DURING PR + MPH | Schedule Length | Genotype x Session | Cox proportional hazards model | 0.08576 | 0.8508 | 1.3953 | 0.497 | n.s. |  |
| Julia | PR | Target Touch Rates | Genotype | GLMM with beta regression | -0.23177 | -0.50345473 | 0.03991559 | 0.0945 | n.s. |  |
| Julia | PR | Target Touch Rates | Session | GLMM with beta regression | -0.075469 | -0.1384174 | -0.01252077 | 0.0188 | $ |  |
| Julia | PR | Target Touch Rates | Genotype x Session | GLMM with beta regression | 0.002629 | -0.08812516 | 0.09338292 | 0.9547 | n.s. |  |
| Julia | PR + ATO | Target Touch Rates | Genotype | GLMM with beta regression | -0.30797 | -0.49845808 | -0.11747818 | 0.00153 | ✱✱ |  |
| Julia | PR + ATO | Target Touch Rates | Drug | GLMM with beta regression | -0.29753 | -0.44672736 | -0.1483284 | 9.29E-05 | @@@ |  |
| Julia | PR + ATO | Target Touch Rates | Session | GLMM with beta regression | 0.01712 | -0.08401998 | 0.11825593 | 0.74009 | n.s. |  |
| Julia | PR + ATO | Target Touch Rates | Genotype x Drug | GLMM with beta regression | 0.24604 | 0.0320162 | 0.46006378 | 0.02425 | % | **WT - KI Saline** P=0.0014 (**)  **WT - KI ATO** P=0.5875 |
| Julia | PR + ATO | Target Touch Rates | Genotype x Session | GLMM with beta regression | -0.0023 | -0.15291309 | 0.14831344 | 0.97612 | n.s. |  |
| Julia | PR + ATO | Target Touch Rates | Drug x Session | GLMM with beta regression | -0.1076 | -0.26700151 | 0.05180838 | 0.18585 | n.s. |  |
| Julia | PR + ATO | Target Touch Rates | Genotype x Drug x Session | GLMM with beta regression | 0.0122 | -0.21588328 | 0.24029051 | 0.91648 | n.s. |  |
| Julia | PR + MPH | Target Touch Rates | Genotype | GLMM with beta regression | -0.3546 | -0.65386414 | -0.05534368 | 0.02021 | ✱ |  |
| Julia | PR + MPH | Target Touch Rates | Drug | GLMM with beta regression | 0.24819 | 0.10633143 | 0.39004865 | 0.000606 | @@@ |  |
| Julia | PR + MPH | Target Touch Rates | Session | GLMM with beta regression | 0.09363 | -0.01416776 | 0.20143188 | 0.088686 | n.s. |  |
| Julia | PR + MPH | Target Touch Rates | Genotype x Drug | GLMM with beta regression | -0.08871 | -0.29787927 | 0.12044956 | 0.405805 | n.s. |  |
| Julia | PR + MPH | Target Touch Rates | Genotype x Session | GLMM with beta regression | -0.10469 | -0.26319193 | 0.05380784 | 0.195461 | n.s. |  |
| Julia | PR + MPH | Target Touch Rates | Drug x Session | GLMM with beta regression | -0.07341 | -0.22618835 | 0.0793595 | 0.346271 | n.s. |  |
| Julia | PR + MPH | Target Touch Rates | Genotype x Drug x Session | GLMM with beta regression | 0.1985 | -0.02734528 | 0.42433857 | 0.084952 | n.s. |  |
| Julia | FR1 | Target Touch Rates | Genotype | GLMM with beta regression | -0.20469 | -0.50030649 | 0.09092931 | 0.175 | n.s. |  |
| Julia | FR1 | Target Touch Rates | Session | GLMM with beta regression | 0.09008 | -0.02264341 | 0.20280241 | 0.117 | n.s. |  |
| Julia | FR1 | Target Touch Rates | Genotype x Session | GLMM with beta regression | 0.06075 | -0.10213146 | 0.22363723 | 0.465 | n.s. |  |
| Julia | FR2 | Target Touch Rates | Genotype | GLMM with beta regression | -0.17906 | -0.366203317 | 0.008087232 | 0.0608 | n.s. |  |
| Julia | FR2 | Target Touch Rates | Session | GLMM with beta regression | 0.04815 | 0.006032859 | 0.090267909 | 0.025 | $ |  |
| Julia | FR2 | Target Touch Rates | Genotype x Session | GLMM with beta regression | 0.01405 | -0.045758192 | 0.073848928 | 0.6453 | n.s. |  |
| Julia | FR3 | Target Touch Rates | Genotype | GLM with beta regression | -0.21571 | -0.4217162 | -0.009703576 | 0.0401 | ✱ |  |
| Julia | FR5 ALL | Target Touch Rates | Genotype | GLMM with beta regression | -0.20948 | -0.355093497 | -0.0638754 | 0.00481 | ✱✱ |  |
| Julia | FR5 ALL | Target Touch Rates | Session | GLMM with beta regression | 0.03341 | 0.004531511 | 0.06228415 | 0.02336 | $ |  |
| Julia | FR5 ALL | Target Touch Rates | Genotype x Session | GLMM with beta regression | 0.044 | 0.003315456 | 0.08468897 | 0.03403 | # | **WT - KI Session 1** P<.0001 (***)  **WT - KI Session 2** P=0.0296 (*)  **WT - KI Session 3** P=0.0054 (**)  **WT - KI Session 4** P=0.003 (**)  **WT - KI Session 5** P=0.0595  **WT - KI Session 6** P=0.0883  **WT - KI Session 7** P=0.2253  **WT - KI Session 8** P=0.2168  **WT - KI Session 9** P=0.0059 (**)  **WT - KI Session 10** P=0.1976  **WT - KI Session 11** P=0.3741  **WT - KI Session 12** P=0.082  **WT - KI Session 13** P=0.0047 (**)  **WT - KI Session 14** P=0.0441 (*)  **WT - KI Session 15** P=0.3051  **WT - KI Session 16** P=0.2403 |
| Julia | FR5 BEFORE PR + ATO | Target Touch Rates | Genotype | GLMM with beta regression | -0.26569 | -0.42611658 | -0.1052612 | 0.001171 | ✱✱ |  |
| Julia | FR5 BEFORE PR + ATO | Target Touch Rates | Session | GLMM with beta regression | 0.01614 | -0.02006729 | 0.05235349 | 0.382238 | n.s. |  |
| Julia | FR5 BEFORE PR + ATO | Target Touch Rates | Genotype x Session | GLMM with beta regression | 0.08847 | 0.0368222 | 0.14011236 | 0.000787 | ### | **WT - KI Session 1** P<.0001 (***)  **WT - KI Session 2** P=0.0203 (*)  **WT - KI Session 3** P=0.0034 (**)  **WT - KI Session 4** P=0.0016 (**)  **WT - KI Session 5** P=0.0422 (*)  **WT - KI Session 6** P=0.0646  **WT - KI Session 7** P=0.1913 |
| Julia | FR5 DURING PR + ATO | Target Touch Rates | Genotype | GLMM with beta regression | -0.169251 | -0.31845147 | -0.02005104 | 0.02619 | ✱ |  |
| Julia | FR5 DURING PR + ATO | Target Touch Rates | Session | GLMM with beta regression | 0.068318 | 0.01686263 | 0.11977295 | 0.00926 | $$ |  |
| Julia | FR5 DURING PR + ATO | Target Touch Rates | Genotype x Session | GLMM with beta regression | 0.009509 | -0.06300694 | 0.08202522 | 0.79717 | n.s. |  |
| Julia | FR5 DURING PR + MPH | Target Touch Rates | Genotype | GLMM with beta regression | -0.17236 | -0.35259876 | 0.007882135 | 0.0609 | n.s. |  |
| Julia | FR5 DURING PR + MPH | Target Touch Rates | Session | GLMM with beta regression | -0.04677 | -0.10060563 | 0.007071357 | 0.0887 | n.s. |  |
| Julia | FR5 DURING PR + MPH | Target Touch Rates | Genotype x Session | GLMM with beta regression | 0.05983 | -0.01571112 | 0.13536714 | 0.1206 | n.s. |  |
| Julia | PR | Target Touches | Genotype | GLMM with negative binomial regression | -0.42126 | -0.8568496 | 0.01433887 | 0.05803 | n.s. |  |
| Julia | PR | Target Touches | Session | GLMM with negative binomial regression | -0.13593 | -0.2263683 | -0.04549888 | 0.00322 | $$ |  |
| Julia | PR | Target Touches | Genotype x Session | GLMM with negative binomial regression | 0.03592 | -0.0910444 | 0.1628837 | 0.57924 | n.s. |  |
| Julia | PR + ATO | Target Touches | Genotype | GLMM with negative binomial regression | -0.14741 | -0.4555195 | 0.160694324 | 0.3484 | n.s. |  |
| Julia | PR + ATO | Target Touches | Drug | GLMM with negative binomial regression | -0.51445 | -0.6961389 | -0.332770458 | 2.86E-08 | @@@ |  |
| Julia | PR + ATO | Target Touches | Session | GLMM with negative binomial regression | -0.06091 | -0.1947322 | 0.072908249 | 0.3723 | n.s. |  |
| Julia | PR + ATO | Target Touches | Genotype x Drug | GLMM with negative binomial regression | -0.25415 | -0.5078527 | -0.00044913 | 0.0496 | % | **WT - KI Saline** P=0.3302  **WT - KI ATO** P=0.0074 (**) |
| Julia | PR + ATO | Target Touches | Genotype x Session | GLMM with negative binomial regression | 0.0333 | -0.1525914 | 0.219197807 | 0.7255 | n.s. |  |
| Julia | PR + ATO | Target Touches | Drug x Session | GLMM with negative binomial regression | -0.02975 | -0.2303972 | 0.170899943 | 0.7714 | n.s. |  |
| Julia | PR + ATO | Target Touches | Genotype x Drug x Session | GLMM with negative binomial regression | -0.05159 | -0.3330315 | 0.229858032 | 0.7194 | n.s. |  |
| Julia | PR + MPH | Target Touches | Genotype | GLMM with negative binomial regression | -0.310551 | -0.67568034 | 0.05457919 | 0.0955 | n.s. |  |
| Julia | PR + MPH | Target Touches | Drug | GLMM with negative binomial regression | 0.338986 | 0.19008284 | 0.48788972 | 8.12E-06 | @@@ |  |
| Julia | PR + MPH | Target Touches | Session | GLMM with negative binomial regression | -0.004331 | -0.11427782 | 0.10561486 | 0.9385 | n.s. |  |
| Julia | PR + MPH | Target Touches | Genotype x Drug | GLMM with negative binomial regression | -0.029383 | -0.23685829 | 0.17809273 | 0.7813 | n.s. |  |
| Julia | PR + MPH | Target Touches | Genotype x Session | GLMM with negative binomial regression | 0.106958 | -0.04630801 | 0.26022336 | 0.1714 | n.s. |  |
| Julia | PR + MPH | Target Touches | Drug x Session | GLMM with negative binomial regression | 0.023949 | -0.13662935 | 0.1845269 | 0.77 | n.s. |  |
| Julia | PR + MPH | Target Touches | Genotype x Drug x Session | GLMM with negative binomial regression | -0.055366 | -0.27963585 | 0.16890287 | 0.6285 | n.s. |  |
| Julia | FR1 | Target Touches | Genotype | GLMM with poisson regression | 0.02273 | -0.06875142 | 0.1142138 | 0.626 | n.s. |  |
| Julia | FR1 | Target Touches | Session | GLMM with poisson regression | 0.02292 | -0.04371018 | 0.08954369 | 0.5 | n.s. |  |
| Julia | FR1 | Target Touches | Genotype x Session | GLMM with poisson regression | -0.02292 | -0.11514617 | 0.06931267 | 0.626 | n.s. |  |
| Julia | FR2 | Target Touches | Genotype | GLMM with poisson regression | 5.41E-13 | -0.06430321 | 0.06430321 | 1 | n.s. |  |
| Julia | FR2 | Target Touches | Session | GLMM with poisson regression | -4.86E-13 | -0.04657391 | 0.04657391 | 1 | n.s. |  |
| Julia | FR2 | Target Touches | Genotype x Session | GLMM with poisson regression | -5.55E-13 | -0.06482814 | 0.06482814 | 1 | n.s. |  |
| Julia | FR3 | Target Touches | Genotype | GLMM with negative binomial regression | -6.75E-09 | -0.07425098 | 0.07425097 | 1 | n.s. |  |
| Julia | FR5 ALL | Target Touches | Genotype | GLMM with poisson regression | 7.94E-09 | -0.01396348 | 0.0139635 | 1 | n.s. |  |
| Julia | FR5 ALL | Target Touches | Session | GLMM with poisson regression | -3.52E-09 | -0.01004088 | 0.01004087 | 1 | n.s. |  |
| Julia | FR5 ALL | Target Touches | Genotype x Session | GLMM with poisson regression | 1.95E-08 | -0.01397588 | 0.01397592 | 1 | n.s. |  |
| Julia | FR5 BEFORE PR + ATO | Target Touches | Genotype | GLMM with poisson regression | -8.93E-09 | -0.02173846 | 0.02173844 | 1 | n.s. |  |
| Julia | FR5 BEFORE PR + ATO | Target Touches | Session | GLMM with poisson regression | -3.48E-10 | -0.01565347 | 0.01565347 | 1 | n.s. |  |
| Julia | FR5 BEFORE PR + ATO | Target Touches | Genotype x Session | GLMM with poisson regression | 7.87E-10 | -0.02178871 | 0.02178871 | 1 | n.s. |  |
| Julia | FR5 DURING PR + ATO | Target Touches | Genotype | GLMM with poisson regression | -2.62E-11 | -0.02581084 | 0.02581084 | 1 | n.s. |  |
| Julia | FR5 DURING PR + ATO | Target Touches | Session | GLMM with poisson regression | -6.30E-12 | -0.01860062 | 0.01860062 | 1 | n.s. |  |
| Julia | FR5 DURING PR + ATO | Target Touches | Genotype x Session | GLMM with poisson regression | -1.29E-11 | -0.025889 | 0.025889 | 1 | n.s. |  |
| Julia | FR5 DURING PR + MPH | Target Touches | Genotype | GLMM with poisson regression | 1.58E-10 | -0.02572128 | 0.02572128 | 1 | n.s. |  |
| Julia | FR5 DURING PR + MPH | Target Touches | Session | GLMM with poisson regression | 3.78E-09 | -0.01853861 | 0.01853862 | 1 | n.s. |  |
| Julia | FR5 DURING PR + MPH | Target Touches | Genotype x Session | GLMM with poisson regression | -4.38E-09 | -0.02580466 | 0.02580465 | 1 | n.s. |  |

**Figure 5**.

| **Experimenter** | **Stage** | **Dependent Variable** | **Independent Variable** | **Stats Test** | **Coefficient** | **Lower 2.5% CI** | **Upper 97.5% CI** | **P-Value** | **Stats Summary** | **Pairwise Notes** |
| --- | --- | --- | --- | --- | --- | --- | --- | --- | --- | --- |
| Jackson | FR1 SL | Active Lever Presses | Genotype | GLMM with poisson regression | -0.73328 | -1.38262914 | -0.08393402 | 0.0269 | ✱ |  |
| Jackson | FR1 SL | Active Lever Presses | Session | GLMM with poisson regression | 0.25405 | 0.22570267 | 0.28239446 | 2.00E-16 | $$$ |  |
| Jackson | FR1 SL | Active Lever Presses | Genotype x Session | GLMM with poisson regression | 0.13163 | 0.09166944 | 0.17159925 | 1.08E-10 | ### | **WT - KI Session 1** P=0.0098 (**)  **WT - KI Session 2** P=0.1313  **WT - KI Session 3** P=0.3797  **WT - KI Session 4** P=0.9108  **WT - KI Session 5** P=0.5294 |
| Jackson | FR1 DL | Active Lever Presses | Genotype | GLMM with poisson regression | -0.0753 | -0.29995688 | 0.14935766 | 0.5112 | n.s. |  |
| Jackson | FR1 DL | Active Lever Presses | Session | GLMM with poisson regression | 0.004957 | -0.02184897 | 0.03176254 | 0.7170 | n.s. |  |
| Jackson | FR1 DL | Active Lever Presses | Genotype x Session | GLMM with poisson regression | 0.046566 | 0.0109404 | 0.08219087 | 0.0104 | # | **WT - KI Session 1** P=0.48  **WT - KI Session 2** P=0.6931  **WT - KI Session 3** P=0.1647  **WT - KI Session 4** P=0.6769 |
| Jackson | FR1 DL | Inactive Lever Presses | Genotype | GLMM with poisson regression | 0.01185 | -0.9389895 | 0.962698123 | 0.9805 | n.s. |  |
| Jackson | FR1 DL | Inactive Lever Presses | Session | GLMM with poisson regression | -0.09295 | -0.1918074 | 0.005915553 | 0.0654 | n.s. |  |
| Jackson | FR1 DL | Inactive Lever Presses | Genotype x Session | GLMM with poisson regression | -0.14681 | -0.3078371 | 0.014221187 | 0.074 | n.s. |  |
| Jackson | FR3 | Active Lever Presses | Genotype | GLMM with poisson regression | 0.184113 | -0.132488124 | 0.50071463 | 0.25438 | n.s. |  |
| Jackson | FR3 | Active Lever Presses | Session | GLMM with poisson regression | 0.062855 | 0.052186102 | 0.07352454 | 2.00E-16 | $$$ |  |
| Jackson | FR3 | Active Lever Presses | Genotype x Session | GLMM with poisson regression | 0.02036 | 0.006673536 | 0.0340467 | 0.00355 | ## | **WT - KI Session 1** P=0.0473 (*)  **WT - KI Session 2** P=0.3823  **WT - KI Session 3** P=0.1659  **WT - KI Session 4** P=0.2658  **WT - KI Session 5** P=0.0463 (*)  **WT - KI Session 6** P=0.037 (*) |
| Jackson | FR3 | Inactive Lever Presses | Genotype | GLMM with poisson regression | -0.66316 | -1.4685001 | 0.1421888 | 0.1065 | n.s. |  |
| Jackson | FR3 | Inactive Lever Presses | Session | GLMM with poisson regression | -0.06956 | -0.128989 | -0.0101401 | 0.0218 | $ |  |
| Jackson | FR3 | Inactive Lever Presses | Genotype x Session | GLMM with poisson regression | 0.01819 | -0.07487732 | 0.1112477 | 0.7017 | n.s. |  |
| Jackson | FR5 ALL | Active Lever Presses | Genotype | GLMM with poisson regression | 0.36744 | -0.011411244 | 0.746291236 | 0.0573 | n.s. |  |
| Jackson | FR5 ALL | Active Lever Presses | Session | GLMM with poisson regression | 0.0051376 | 0.004599627 | 0.005675616 | 2.00E-16 | $$$ |  |
| Jackson | FR5 ALL | Active Lever Presses | Genotype x Session | GLMM with poisson regression | -0.0005014 | -0.00119021 | 0.000187457 | 0.1537 | n.s. |  |
| Jackson | FR5 ALL | Inactive Lever Presses | Genotype | GLMM with negative binomial regression | -0.483549 | -1.251727 | 0.284628964 | 0.217297 | n.s. |  |
| Jackson | FR5 ALL | Inactive Lever Presses | Session | GLMM with negative binomial regression | -0.013927 | -0.02195821 | -0.005896528 | 6.76E-04 | $$$ |  |
| Jackson | FR5 ALL | Inactive Lever Presses | Genotype x Session | GLMM with negative binomial regression | -0.006097 | -0.01791962 | 0.005726041 | 3.12E-01 | n.s. |  |
| Jackson | FR5 BEFORE PR | Active Lever Presses | Genotype | GLMM with poisson regression | 0.214738 | -0.17783264 | 0.60730883 | 0.284 | n.s. |  |
| Jackson | FR5 BEFORE PR | Active Lever Presses | Session | GLMM with poisson regression | -0.019534 | -0.02564706 | -0.01342043 | 3.79E-10 | $$$ |  |
| Jackson | FR5 BEFORE PR | Active Lever Presses | Genotype x Session | GLMM with poisson regression | 0.032154 | 0.02440968 | 0.03989863 | 4.03E-16 | ### | **WT - KI Session 1** P=0.1321  **WT - KI Session 2** P=0.0818  **WT - KI Session 3** P=0.1089  **WT - KI Session 4** P=0.5712  **WT - KI Session 5** P=0.0627  **WT - KI Session 6** P=0.0437 (*)  **WT - KI Session 7** P=0.0029 (**)  **WT - KI Session 8** P=0.0212 (*) |
| Jackson | FR5 BEFORE PR | Inactive Lever Presses | Genotype | GLMM with poisson regression | -0.05088 | -0.98951955 | 0.88776171 | 0.915392 | n.s. |  |
| Jackson | FR5 BEFORE PR | Inactive Lever Presses | Session | GLMM with poisson regression | 0.11992 | 0.08362803 | 0.1562185 | 9.42E-11 | $$$ |  |
| Jackson | FR5 BEFORE PR | Inactive Lever Presses | Genotype x Session | GLMM with poisson regression | -0.10753 | -0.16433922 | -0.05071886 | 0.000207 | ### | **WT - KI Session 1** P=0.9928  **WT - KI Session 2** P=0.229  **WT - KI Session 3** P=0.387  **WT - KI Session 4** P=0.9324  **WT - KI Session 5** P=0.0573  **WT - KI Session 6** P=0.4604  **WT - KI Session 7** P=0.2272  **WT - KI Session 8** P=0.0529 |
| Jackson | FR5 BASELINING | Active Lever Presses | Genotype | GLMM with negative binomial regression | 0.3583629 | -0.140388957 | 0.857114695 | 0.15905 | n.s. |  |
| Jackson | FR5 BASELINING | Active Lever Presses | Session | GLMM with negative binomial regression | 0.0088678 | 0.002801515 | 0.01493404 | 0.00417 | $$ |  |
| Jackson | FR5 BASELINING | Active Lever Presses | Genotype x Session | GLMM with negative binomial regression | 0.0008653 | -0.007341459 | 0.009072067 | 0.83628 | n.s. |  |
| Jackson | FR5 BASELINING | Inactive Lever Presses | Genotype | GLMM with poisson regression | -0.313138 | -1.13830846 | 0.51203257 | 0.45701 | n.s. |  |
| Jackson | FR5 BASELINING | Inactive Lever Presses | Session | GLMM with poisson regression | -0.001797 | -0.008115346 | 0.004521003 | 5.77E-01 | n.s. |  |
| Jackson | FR5 BASELINING | Inactive Lever Presses | Genotype x Session | GLMM with poisson regression | -0.015687 | -0.027057174 | -0.004316974 | 6.85E-03 | ## | **WT - KI Session 1** P=0.5415  **WT - KI Session 2** P=0.9326  **WT - KI Session 3** P=0.8332  **WT - KI Session 4** P=0.6393  **WT - KI Session 5** P=0.0089 (**)  **WT - KI Session 6** P=0.0817  **WT - KI Session 7** P=0.0543  **WT - KI Session 8** P=0.0008 (***)  **WT - KI Session 9** P=0.1324  **WT - KI Session 10** P=0.0117 (*)  **WT - KI Session 11** P=0.0101 (*)  **WT - KI Session 12** P=0.0018 (**)  **WT - KI Session 13** P=0.0314 (*)  **WT - KI Session 14** P=0.3882  **WT - KI Session 15** P=0.235  **WT - KI Session 16** P=0.2276  **WT - KI Session 17** P=0.1134  **WT - KI Session 18** P=0.3344  **WT - KI Session 19** P=0.1592  **WT - KI Session 20** P=0.0129 (*) |
| Jackson | PR 2HR | Active Lever Presses | Genotype | GLMM with poisson regression | 0.2605032 | -0.098511839 | 0.619518179 | 0.155 | n.s. |  |
| Jackson | PR 2HR | Active Lever Presses | Session | GLMM with poisson regression | -0.0092934 | -0.010361446 | -0.008225344 | 2.00E-16 | $$$ |  |
| Jackson | PR 2HR | Active Lever Presses | Genotype x Session | GLMM with poisson regression | 0.008891 | 0.007565178 | 0.010216757 | 2.00E-16 | ### | **WT - KI Session 1** P=0.0412 (*)  **WT - KI Session 2** P=0.0162 (*)  **WT - KI Session 3** P=0.25  **WT - KI Session 4** P=0.0233 (*)  **WT - KI Session 5** P=0.0973  **WT - KI Session 6** P=0.0294 (*)  **WT - KI Session 7** P=0.0002 (***)  **WT - KI Session 8** P=0.0019 (**)  **WT - KI Session 9** P=0.0044 (**)  **WT - KI Session 10** P=0.0015 (**)  **WT - KI Session 11** P=0.0058 (**) |
| Jackson | PR 2HR | Inactive Lever Presses | Genotype | GLMM with poisson regression | -0.08386 | -1.06804854 | 0.900327771 | 0.86737 | n.s. |  |
| Jackson | PR 2HR | Inactive Lever Presses | Session | GLMM with poisson regression | -0.007278 | -0.01214188 | -0.002413192 | 0.00336 | $$ |  |
| Jackson | PR 2HR | Inactive Lever Presses | Genotype x Session | GLMM with poisson regression | -0.013998 | -0.02218902 | -0.005806759 | 8.10E-04 | ### | **WT - KI Session 1** P=0.7724  **WT - KI Session 2** P=0.9199  **WT - KI Session 3** P=0.1731  **WT - KI Session 4** P=0.8454  **WT - KI Session 5** P=0.2544  **WT - KI Session 6** P=0.023 (*)  **WT - KI Session 7** P=0.2737  **WT - KI Session 8** P=0.2496  **WT - KI Session 9** P=0.2451  **WT - KI Session 10** P=0.1254  **WT - KI Session 11** P=0.9849 |
| Jackson | PR 2HR + ATO | Active Lever Presses | Genotype | GLMM with negative binomial regression | 0.4432 | 0.07946827 | 0.80693144 | 0.0169 | ✱ |  |
| Jackson | PR 2HR + ATO | Active Lever Presses | Drug | GLMM with negative binomial regression | 0.172 | -0.15294771 | 0.49695593 | 0.2995 | n.s. |  |
| Jackson | PR 2HR + ATO | Active Lever Presses | Dose | GLMM with negative binomial regression | -0.13928 | -0.26314643 | -0.01541909 | 0.0275 | & | **Saline - 1 mg/kg ATO** P=0.9721  **Saline - 3 mg/kg ATO** P=0.0009 (***)  **1 mg/kg - 3 mg/kg ATO** P=0.0023 (**) |
| Jackson | PR 2HR + ATO | Active Lever Presses | Genotype x Drug | GLMM with negative binomial regression | -0.0988 | -0.53253974 | 0.33493925 | 0.6553 | n.s. |  |
| Jackson | PR 2HR + ATO | Active Lever Presses | Genotype x Dose | GLMM with negative binomial regression | -0.00441 | -0.16957634 | 0.16075619 | 0.9583 | n.s. |  |
| Jackson | PR 2HR + ATO | Inactive Lever Presses | Genotype | GLMM with negative binomial regression | -0.79686 | -1.8141176 | 0.2203932 | 0.125 | n.s. |  |
| Jackson | PR 2HR + ATO | Inactive Lever Presses | Drug | GLMM with negative binomial regression | -0.21919 | -0.7287048 | 0.2903282 | 0.399 | n.s. |  |
| Jackson | PR 2HR + ATO | Inactive Lever Presses | Dose | GLMM with negative binomial regression | -0.08755 | -0.2824155 | 0.1073216 | 0.379 | n.s. |  |
| Jackson | PR 2HR + ATO | Inactive Lever Presses | Genotype x Drug | GLMM with negative binomial regression | 0.26655 | -0.5250733 | 1.0581679 | 0.509 | n.s. |  |
| Jackson | PR 2HR + ATO | Inactive Lever Presses | Genotype x Dose | GLMM with negative binomial regression | -0.04867 | -0.3551949 | 0.2578482 | 0.756 | n.s. |  |
| Jackson | FR10 | Active Lever Presses | Genotype | GLMM with negative binomial regression | -0.03658 | -0.5332629 | 0.4601062 | 0.885 | n.s. |  |
| Jackson | FR10 | Inactive Lever Presses | Genotype | GLMM with negative binomial regression | -1.1394 | -2.140146 | -0.1387252 | 0.0256 | ✱ |  |
| Jackson | FR20 | Active Lever Presses | Genotype | GLMM with negative binomial regression | 0.05398 | -0.4585541 | 0.5665126 | 0.836 | n.s. |  |
| Jackson | FR20 | Inactive Lever Presses | Genotype | GLMM with negative binomial regression | -0.7538 | -1.48967 | -0.0178737 | 0.0447 | ✱ |  |
| Jackson | FR40 | Active Lever Presses | Genotype | GLMM with negative binomial regression | -0.03649 | -0.8195749 | 0.7465984 | 0.927 | n.s. |  |
| Jackson | FR40 | Inactive Lever Presses | Genotype | GLMM with negative binomial regression | -0.4534 | -1.227956 | 0.3211687 | 0.251 | n.s. |  |

**Figure 6**.

| **Experimenter** | **Stage** | **Dependent Variable** | **Independent Variable** | **Stats Test** | **Coefficient** | **Lower 2.5% CI** | **Upper 97.5% CI** | **P-Value** | **Stats Summary** | **Pairwise Notes** |
| --- | --- | --- | --- | --- | --- | --- | --- | --- | --- | --- |
| Jackson | FR1 SL | Active Lever Presses | Genotype | GLMM with negative binomial regression | 0.28113 | -0.18984545 | 0.7521055 | 0.242 | n.s. |  |
| Jackson | FR1 SL | Active Lever Presses | Session | GLMM with negative binomial regression | 0.62143 | 0.50054085 | 0.7423279 | 2.00E-16 | $$$ |  |
| Jackson | FR1 SL | Active Lever Presses | Genotype x Session | GLMM with negative binomial regression | -0.05125 | -0.21881371 | 0.1163133 | 0.549 | n.s. |  |
| Jackson | FR1 DL | Active Lever Presses | Genotype | GLMM with poisson regression | 0.123253 | -0.06518941 | 0.31169528 | 0.2 | n.s. |  |
| Jackson | FR1 DL | Active Lever Presses | Session | GLMM with poisson regression | 0.057816 | 0.03567682 | 0.07995435 | 3.08E-07 | $$$ |  |
| Jackson | FR1 DL | Active Lever Presses | Genotype x Session | GLMM with poisson regression | -0.008903 | -0.03972778 | 0.02192128 | 0.571 | n.s. |  |
| Jackson | FR1 DL | Inactive Lever Presses | Genotype | GLMM with poisson regression | -0.51998 | -1.23656111 | 0.196598 | 0.154958 | n.s. |  |
| Jackson | FR1 DL | Inactive Lever Presses | Session | GLMM with poisson regression | -0.26873 | -0.34195105 | -0.1955165 | 6.30E-13 | $$$ |  |
| Jackson | FR1 DL | Inactive Lever Presses | Genotype x Session | GLMM with poisson regression | 0.1912 | 0.08930492 | 0.2931024 | 0.000235 | ### | **WT - KI Session 1** P=0.2751  **WT - KI Session 2** P=0.7825  **WT - KI Session 3** P=0.2608  **WT - KI Session 4** P=0.9958 |
| Jackson | FR3 | Active Lever Presses | Genotype | GLMM with poisson regression | 0.104488 | -0.12735378 | 0.336328997 | 0.377059 | n.s. |  |
| Jackson | FR3 | Active Lever Presses | Session | GLMM with poisson regression | -0.019333 | -0.03460454 | -0.004061102 | 0.013095 | $ |  |
| Jackson | FR3 | Active Lever Presses | Genotype x Session | GLMM with poisson regression | 0.036187 | 0.01567706 | 0.056696448 | 0.000544 | ### | **WT - KI Session 1** P=0.3138  **WT - KI Session 2** P=0.0902  **WT - KI Session 3** P=0.0396 (*)  **WT - KI Session 4** P=0.0558 |
| Jackson | FR3 | Inactive Lever Presses | Genotype | GLMM with poisson regression | -0.48659 | -1.28275145 | 0.3095719 | 0.231 | n.s. |  |
| Jackson | FR3 | Inactive Lever Presses | Session | GLMM with poisson regression | -0.03487 | -0.11887356 | 0.04912453 | 0.416 | n.s. |  |
| Jackson | FR3 | Inactive Lever Presses | Genotype x Session | GLMM with poisson regression | 0.04074 | -0.08602118 | 0.16750062 | 0.529 | n.s. |  |
| Jackson | FR5 ALL | Active Lever Presses | Genotype | GLMM with poisson regression | 0.1737432 | -0.076866235 | 0.424352569 | 0.174 | n.s. |  |
| Jackson | FR5 ALL | Active Lever Presses | Session | GLMM with poisson regression | 0.0093507 | 0.008491619 | 0.01020972 | 2.00E-16 | $$$ |  |
| Jackson | FR5 ALL | Active Lever Presses | Genotype x Session | GLMM with poisson regression | -0.0047228 | -0.005918802 | -0.003526814 | 9.97E-15 | ### | **WT - KI Session 1** P=0.0155 (*)  **WT - KI Session 2** P=0.0502  **WT - KI Session 3** P=0.1378  **WT - KI Session 4** P=0.2730  **WT - KI Session 5** P=0.222  **WT - KI Session 6** P=0.2877  **WT - KI Session 7** P=0.5253  **WT - KI Session 8** P=0.2403  **WT - KI Session 9** P=0.9703  **WT - KI Session 10** P=0.3403  **WT - KI Session 11** P=0.4183  **WT - KI Session 12** P=0.2967  **WT - KI Session 13** P=0.1298  **WT- KI Session 14** P=0.3988  **WT - KI Session 15** P=0.8412  **WT - KI Session 16** P=0.2582  **WT - KI Session 17** P=0.5017  **WT - KI Session 18** P=0.4588  **WT - KI Session 19** P=0.1359  **WT - KI Session 20** P=0.1492  **WT - KI Session 21** P=0.3556  **WT - KI Session 22** P=0.2209  **WT - KI Session 23** P=0.6616 |
| Jackson | FR5 ALL | Inactive Lever Presses | Genotype | GLMM with poisson regression | 3.79E-01 | -0.220208164 | 0.97845392 | 0.215 | n.s. |  |
| Jackson | FR5 ALL | Inactive Lever Presses | Session | GLMM with poisson regression | -7.93E-05 | -0.006678165 | 0.00651948 | 0.981 | n.s. |  |
| Jackson | FR5 ALL | Inactive Lever Presses | Genotype x Session | GLMM with poisson regression | -2.56E-02 | -0.035029129 | -0.01620245 | 9.63E-08 | ### | **WT - KI Session 1** P=0.6558  **WT - KI Session 2** P=0.3762  **WT - KI Session 3** P=0.0784  **WT - KI Session 4** P=0.0632  **WT - KI Session 5** P=0.4689  **WT - KI Session 6** P=0.4757  **WT - KI Session 7** P=0.3170  **WT - KI Session 8** P=0.7651  **WT - KI Session 9** P=0.0366 (*)  **WT - KI Session 10** P=0.8617  **WT - KI Session 11** P=0.1913  **WT - KI Session 12** P=0.4737  **WT - KI Session 13** P=0.2728  **WT - KI Session 14** P=0.7326  **WT - KI Session 15** P=0.9279  **WT - KI Session 16** P=0.9628  **WT - KI Session 17** P=0.9014  **WT - KI Session 18** P=0.9694  **WT - KI Session 19** P=0.0192 (*)  **WT - KI Session 20** P=0.8282  **WT - KI Session 21** P=0.6037  **WT - KI Session 22** P=0.2416  **WT - KI Session 23** P=0.2548 |
| Jackson | FR5 PRIOR TO DEVAL | Active Lever Presses | Genotype | GLMM with poisson regression | 0.342778 | 0.03245825 | 0.653098335 | 0.0304 | ✱ |  |
| Jackson | FR5 PRIOR TO DEVAL | Active Lever Presses | Session | GLMM with poisson regression | -0.006509 | -0.01901102 | 0.005992148 | 0.3075 | n.s. |  |
| Jackson | FR5 PRIOR TO DEVAL | Active Lever Presses | Genotype x Session | GLMM with poisson regression | -0.055227 | -0.07191324 | -0.038541228 | 8.75E-11 | ### | **WT - KI Session 1** P=0.0637  **WT - KI Session 2** P=0.1397  **WT - KI Session 3** P=0.2764  **WT - KI Session 4** P=0.4404 |
| Jackson | FR5 PRIOR TO DEVAL | Inactive Lever Presses | Genotype | GLMM with poisson regression | -0.42796 | -1.270217 | 0.41429114 | 0.319 | n.s. |  |
| Jackson | FR5 PRIOR TO DEVAL | Inactive Lever Presses | Session | GLMM with poisson regression | -0.18076 | -0.2694053 | -0.09210914 | 6.43E-05 | $$$ |  |
| Jackson | FR5 PRIOR TO DEVAL | Inactive Lever Presses | Genotype x Session | GLMM with poisson regression | 0.24455 | 0.1271765 | 0.36193173 | 4.44E-05 | ### | **WT - KI Session 1** P=0.4729  **WT - KI Session 2** P=0.7562  **WT - KI Session 3** P=0.3134  **WT - KI Session 4** P=0.2777 |
| Jackson | FR5 FROM DEVAL TO PR | Active Lever Presses | Genotype | GLMM with poisson regression | 0.034214 | -0.240348562 | 0.30877736 | 0.807 | n.s. |  |
| Jackson | FR5 FROM DEVAL TO PR | Active Lever Presses | Session | GLMM with poisson regression | 0.040554 | 0.034039438 | 0.04706806 | 2.00E-16 | $$$ |  |
| Jackson | FR5 FROM DEVAL TO PR | Active Lever Presses | Genotype x Session | GLMM with poisson regression | 0.007196 | -0.001875755 | 0.01626741 | 0.12 | n.s. |  |
| Jackson | FR5 FROM DEVAL TO PR | Inactive Lever Presses | Genotype | GLMM with poisson regression | 1.36539 | 0.408523077 | 2.3222477 | 0.005162 | ✱✱ |  |
| Jackson | FR5 FROM DEVAL TO PR | Inactive Lever Presses | Session | GLMM with poisson regression | 0.05883 | 0.007902583 | 0.1097646 | 0.023569 | $ |  |
| Jackson | FR5 FROM DEVAL TO PR | Inactive Lever Presses | Genotype x Session | GLMM with poisson regression | -0.1375 | -0.210276139 | -0.0647263 | 0.000213 | ### | **WT - KI Session 1** P=0.3897  **WT - KI Session 2** P=0.8261  **WT - KI Session 3** P=0.0624  **WT - KI Session 4** P=0.8299  **WT - KI Session 5** P=0.2511  **WT - KI Session 6** P=0.544 |
| Jackson | FR5 DURING PR | Active Lever Presses | Genotype | GLMM with poisson regression | 0.27834 | 0.003930566 | 0.5527488 | 0.0468 | ✱ |  |
| Jackson | FR5 DURING PR | Active Lever Presses | Session | GLMM with poisson regression | 0.01972 | 0.01705919 | 0.02238127 | 2.00E-16 | $$$ |  |
| Jackson | FR5 DURING PR | Active Lever Presses | Genotype x Session | GLMM with poisson regression | -0.022144 | -0.025769138 | -0.018518 | 2.00E-16 | ### | **WT - KI Session 1** P=0.0297 (*)  **WT - KI Session 2** P=0.0799  **WT - KI Session 3** P=0.188  **WT - KI Session 4** P=0.3366  **WT - KI Session 5** P=0.2819  **WT - KI Session 6** P=0.352  **WT - KI Session 7** P=0.6003  **WT - KI Session 8** P=0.302  **WT - KI Session 9** P=0.9366  **WT - KI Session 10** P=0.4068  **WT - KI Session 11** P=0.4856 |
| Jackson | FR5 DURING PR | Inactive Lever Presses | Genotype | GLMM with poisson regression | 0.33361 | -0.38446828 | 1.051691025 | 0.363 | n.s. |  |
| Jackson | FR5 DURING PR | Inactive Lever Presses | Session | GLMM with poisson regression | -0.02357 | -0.0434306 | -0.003705382 | 0.02 | $ |  |
| Jackson | FR5 DURING PR | Inactive Lever Presses | Genotype x Session | GLMM with poisson regression | -0.02801 | -0.05513931 | -0.000880108 | 0.043 | # | **WT - KI Session 1** P=0.5989  **WT - KI Session 2** P=0.5364  **WT - KI Session 3** P=0.1663  **WT - KI Session 4** P=0.1417  **WT - KI Session 5** P=0.6241  **WT - KI Session 6** P=0.6283  **WT - KI Session 7** P=0.466  **WT - KI Session 8** P=0.902  **WT - KI Session 9** P=0.0921  **WT - KI Session 10** P=0.7711  **WT - KI Session 11** P=0.2016 |
| Jackson | FR5 DEVAL | Active Lever Presses | Genotype | GLMM with poisson regression | 4.79E-01 | 0.12308661 | 0.83580439 | 0.00837 | ✱✱ |  |
| Jackson | FR5 DEVAL | Active Lever Presses | Session | GLMM with poisson regression | -7.93E-08 | -0.08223834 | 0.08223819 | 1 | n.s. |  |
| Jackson | FR5 DEVAL | Active Lever Presses | Genotype x Session | GLMM with poisson regression | -0.1561 | -0.2652383 | -0.04697713 | 0.00505 | ## | **WT - KI Session 1** P=0.0489 (*)  **WT - KI Session 2** P=0.3094 |
| Jackson | FR5 DEVAL | Inactive Lever Presses | Genotype | GLMM with poisson regression | 1.491816 | 0.4091054 | 2.5745273 | 0.006923 | ✱✱ |  |
| Jackson | FR5 DEVAL | Inactive Lever Presses | Session | GLMM with poisson regression | 0.653927 | 0.266992 | 1.0408612 | 0.000925 | $$$ |  |
| Jackson | FR5 DEVAL | Inactive Lever Presses | Genotype x Session | GLMM with poisson regression | -0.999672 | -1.5403458 | -0.4589989 | 0.00029 | ### | **WT - KI Session 1** P=0.1993  **WT - KI Session 2** P=0.1766 |
| Jackson | PR 2HR | Active Lever Presses | Genotype | GLMM with poisson regression | -0.059383 | -0.31004107 | 0.1912759 | 0.642 | n.s. |  |
| Jackson | PR 2HR | Active Lever Presses | Session | GLMM with poisson regression | 0.019184 | 0.01316859 | 0.02520032 | 4.10E-10 | $$$ |  |
| Jackson | PR 2HR | Active Lever Presses | Genotype x Session | GLMM with poisson regression | 0.036524 | 0.02800781 | 0.04504062 | 2.00E-16 | ### | **WT - KI Session 1** P=0.5351  **WT - KI Session 2** P=0.2808  **WT - KI Session 3** P=0.5534  **WT - KI Session 4** P=0.6175  **WT - KI Session 5** P=0.2204 |
| Jackson | PR 2HR | Inactive Lever Presses | Genotype | GLMM with poisson regression | 0.027377 | -0.64352385 | 0.69827691 | 0.9363 | n.s. |  |
| Jackson | PR 2HR | Inactive Lever Presses | Session | GLMM with poisson regression | -0.000757 | -0.03888833 | 0.03737433 | 0.969 | n.s. |  |
| Jackson | PR 2HR | Inactive Lever Presses | Genotype x Session | GLMM with poisson regression | 0.070636 | 0.01903279 | 0.12223825 | 0.0073 | ## | **WT - KI Session 1** P=0.9314  **WT - KI Session 2** P=0.6754  **WT - KI Session 3** P=0.0982  **WT - KI Session 4** P=0.3337  **WT - KI Session 5** P=0.4923 |
| Jackson | PR 2HR + ATO | Active Lever Presses | Genotype | GLMM with poisson regression | 0.039124 | -0.26270432 | 0.34095258 | 0.799 | n.s. |  |
| Jackson | PR 2HR + ATO | Active Lever Presses | Drug | GLMM with poisson regression | -0.112024 | -0.15189618 | -0.07215124 | 3.66E-08 | @@@ |  |
| Jackson | PR 2HR + ATO | Active Lever Presses | Dose | GLMM with poisson regression | -0.162157 | -0.17895639 | -0.14535681 | 2.00E-16 | &&& | **Saline - 1 mg/kg ATO** P<.0001 (***)  **Saline - 3 mg/kg ATO** P<.0001 (***)  **1 mg/kg - 3 mg/kg ATO** P<.0001 (***) |
| Jackson | PR 2HR + ATO | Active Lever Presses | Genotype x Drug | GLMM with poisson regression | -0.033667 | -0.08915404 | 0.02182002 | 0.234 | n.s. |  |
| Jackson | PR 2HR + ATO | Active Lever Presses | Genotype x Dose | GLMM with poisson regression | 0.095424 | 0.07260425 | 0.11824377 | 2.49E-16 | +++ | **WT - KI Saline** P=0.7995  **WT - KI 1 mg/kg ATO** P=0.5129  **WT - KI 3 mg/kg ATO** P=0.0588 |
| Jackson | PR 2HR + ATO | Inactive Lever Presses | Genotype | GLMM with negative binomial regression | 0.108 | -0.84194813 | 1.0580455 | 0.8236 | n.s. |  |
| Jackson | PR 2HR + ATO | Inactive Lever Presses | Drug | GLMM with negative binomial regression | -1.029 | -1.75496576 | -0.3030907 | 0.00546 | @@ |  |
| Jackson | PR 2HR + ATO | Inactive Lever Presses | Dose | GLMM with negative binomial regression | 0.1919 | -0.08443254 | 0.4682564 | 0.17347 | n.s. |  |
| Jackson | PR 2HR + ATO | Inactive Lever Presses | Genotype x Drug | GLMM with negative binomial regression | 0.7231 | -0.28116945 | 1.7273839 | 0.15818 | n.s. |  |
| Jackson | PR 2HR + ATO | Inactive Lever Presses | Genotype x Dose | GLMM with negative binomial regression | -0.2081 | -0.59064586 | 0.174408 | 0.28627 | n.s. |  |

**Figure 7**.

| **Experimenter** | **Stage** | **Dependent Variable** | **Independent Variable** | **Stats Test** | **Coefficient** | **Lower 2.5% CI** | **Upper 97.5% CI** | **P-Value** | **Stats Summary** | **Pairwise Notes** |
| --- | --- | --- | --- | --- | --- | --- | --- | --- | --- | --- |
| Jackson | HABITUATION | Total Distance | Genotype | Linear regression | 1089.6 | 122.8528 | 2056.392 | 0.0294 | ✱ |  |
| Jackson | HABITUATION | Time Preference | Genotype | Linear regression | 0.03322 | -0.1525383 | 0.2189827 | 0.711 | n.s. |  |
| Jackson | SALINE CONDITIONING | Total Distance | Genotype | Linear mixed effects regression | 797.5 | -719.6325 | 2314.72698 | 0.3193 | n.s. |  |
| Jackson | SALINE CONDITIONING | Total Distance | Session | Linear mixed effects regression | -479.2 | -913.5281 | -44.96612 | 0.0351 | $ |  |
| Jackson | SALINE CONDITIONING | Total Distance | Genotype x Session | Linear mixed effects regression | 116.9 | -481.7107 | 715.51934 | 0.7036 | n.s. |  |
| Jackson | COCAINE CONDITIONING | Total Distance | Genotype | Linear mixed effects regression | -1739.63 | -3642.1277 | 162.8667 | 0.09218 | n.s. |  |
| Jackson | COCAINE CONDITIONING | Total Distance | Session | Linear mixed effects regression | -29.01 | -947.8756 | 889.8517 | 0.95093 | n.s. |  |
| Jackson | COCAINE CONDITIONING | Total Distance | Genotype x Session | Linear mixed effects regression | -1749.55 | -3016.1119 | -482.9797 | 0.00908 | ## | **WT - KI Session 1** P=0.4779  **WT - KI Session 2** P=0.3166  **WT - KI Session 3** P=0.0612  **WT - KI Session 4** P=0.0169 (*) |
| Jackson | CPP | Total Distance | Genotype | Linear regression | 1424.1 | 282.7288 | 2565.56 | 0.0175 | ✱ |  |
| Jackson | CPP | Time Preference | Genotype | Linear regression | -0.1259 | -0.4646058 | 0.212828 | 0.444 | n.s. |  |
| Jackson | EPP ALL | Total Distance | Genotype | Linear mixed effects regression | 1354.17 | 246.4252 | 2526.2226 | 0.0369 | ✱ |  |
| Jackson | EPP ALL | Total Distance | Session | Linear mixed effects regression | 301.28 | -287.3187 | 873.4488 | 0.331 | n.s. |  |
| Jackson | EPP ALL | Total Distance | Genotype x Session | Linear mixed effects regression | -213.66 | -1129.008 | 659.5446 | 0.6503 | n.s. |  |
| Jackson | EPP ALL | Time Preference | Genotype | Linear mixed effects regression | -0.034402 | -0.5202614 | 0.43525538 | 0.8898 | n.s. |  |
| Jackson | EPP ALL | Time Preference | Session | Linear mixed effects regression | -0.243818 | -0.4216869 | 0.06284412 | 0.0277 | $ |  |
| Jackson | EPP ALL | Time Preference | Genotype x Session | Linear mixed effects regression | -0.005382 | -0.279263 | 0.30517969 | 0.9711 | n.s. |  |
| Jackson | EPP PROGRESSION | Total Distance | Genotype | Linear regression | 1496.2 | -11.85324 | 3004.353 | 0.0516 | n.s. |  |
| Jackson | EPP PROGRESSION | Time Preference | Genotype | Linear regression | 0.0725 | -0.1667566 | 0.3117566 | 0.53 | n.s. |  |
| Jackson | EXTINCTION | Number of Trials | Genotype | GLM with poisson regression | -0.1542 | -0.88751865 | 0.5854016 | 0.6783 | n.s. |  |
| Jackson | REINSTATEMENT | Total Distance | Genotype | Linear regression | 559.5 | -2486.086 | 3605.036 | 0.702 | n.s. |  |
| Jackson | REINSTATEMENT | Time Preference | Genotype | Linear regression | 0.2902 | -0.2924315 | 0.8729315 | 0.343 | n.s. |  |

**Figure 8**.

| **Experimenter** | **Stage** | **Dependent Variable** | **Independent Variable** | **Stats Test** | **Coefficient** | **Lower 2.5% CI** | **Upper 97.5% CI** | **P-Value** | **Stats Summary** | **Pairwise Notes** |
| --- | --- | --- | --- | --- | --- | --- | --- | --- | --- | --- |
| Riki | FR1 SL | Active Lever Presses | Genotype | GLMM with poisson regression | -0.10319 | -0.269779371 | 0.06339447 | 0.225 | n.s. |  |
| Riki | FR1 SL | Active Lever Presses | Lever Location | GLMM with poisson regression | -0.04033 | -0.206796891 | 0.12613009 | 0.635 | n.s. |  |
| Riki | FR1 SL | Active Lever Presses | Session | GLMM with poisson regression | -0.0112 | -0.032488224 | 0.01009652 | 0.303 | n.s. |  |
| Riki | FR1 SL | Active Lever Presses | Genotype x Lever Location | GLMM with poisson regression | 0.01521 | -0.220455922 | 0.25087935 | 0.899 | n.s. |  |
| Riki | FR1 SL | Active Lever Presses | Session x Lever Location | GLMM with poisson regression | 0.01062 | -0.014587916 | 0.03583647 | 0.409 | n.s. |  |
| Riki | FR1 SL | Active Lever Presses | Genotype x Session | GLMM with poisson regression | 0.01985 | -0.005391308 | 0.04508443 | 0.123 | n.s. |  |
| Riki | FR1 DL | Active Lever Presses | Genotype | GLMM with poisson regression | -0.084335 | -0.20224153 | 0.03357218 | 0.161 | n.s. |  |
| Riki | FR1 DL | Active Lever Presses | Lever Location | GLMM with poisson regression | -0.084956 | -0.20286885 | 0.0329563 | 0.158 | n.s. |  |
| Riki | FR1 DL | Active Lever Presses | Session | GLMM with poisson regression | 0.044719 | 0.02418684 | 0.06525091 | 1.97E-05 | $$$ |  |
| Riki | FR1 DL | Active Lever Presses | Genotype x Lever Location | GLMM with poisson regression | 0.044884 | -0.12207431 | 0.21184209 | 0.598 | n.s. |  |
| Riki | FR1 DL | Active Lever Presses | Session x Lever Location | GLMM with poisson regression | -0.014018 | -0.03834068 | 0.01030479 | 0.259 | n.s. |  |
| Riki | FR1 DL | Active Lever Presses | Genotype x Session | GLMM with poisson regression | -0.001164 | -0.02548734 | 0.02315945 | 0.925 | n.s. |  |
| Riki | FR1 DL | Inactive Lever Presses | Genotype | GLMM with poisson regression | -0.34963 | -1.97825782 | 1.27900217 | 0.673932 | n.s. |  |
| Riki | FR1 DL | Inactive Lever Presses | Lever Location | GLMM with poisson regression | -0.91785 | -2.55266521 | 0.71697141 | 0.27116 | n.s. |  |
| Riki | FR1 DL | Inactive Lever Presses | Session | GLMM with poisson regression | -0.4708 | -0.59543083 | -0.34616653 | 1.32E-13 | $$$ |  |
| Riki | FR1 DL | Inactive Lever Presses | Genotype x Lever Location | GLMM with poisson regression | 1.24289 | -1.06882524 | 3.55461291 | 0.291987 | n.s. |  |
| Riki | FR1 DL | Inactive Lever Presses | Session x Lever Location | GLMM with poisson regression | 0.21855 | 0.07658246 | 0.36051003 | 0.002551 | << | **Beside - Opposite Session 1** P=0.2883  **Beside - Opposite Session 2** P=0.8868  **Beside - Opposite Session 3** P=0.6207  **Beside - Opposite Session 4** P=0.8403 |
| Riki | FR1 DL | Inactive Lever Presses | Genotype x Session | GLMM with poisson regression | -0.09535 | -0.24184884 | 0.05114604 | 0.202065 | n.s. |  |
| Riki | FR3 | Active Lever Presses | Genotype | GLMM with poisson regression | -0.094359 | -0.245335554 | 0.05661779 | 0.2206 | n.s. |  |
| Riki | FR3 | Active Lever Presses | Lever Location | GLMM with poisson regression | -0.165191 | -0.316211609 | -0.01417035 | 0.032 | ~ |  |
| Riki | FR3 | Active Lever Presses | Session | GLMM with poisson regression | 0.043942 | 0.032706916 | 0.05517725 | 1.78E-14 | $$$ |  |
| Riki | FR3 | Active Lever Presses | Genotype x Lever Location | GLMM with poisson regression | 0.002114 | -0.21154527 | 0.21577353 | 0.9845 | n.s. |  |
| Riki | FR3 | Active Lever Presses | Session x Lever Location | GLMM with poisson regression | -0.011326 | -0.024902822 | 0.00225034 | 0.102 | n.s. |  |
| Riki | FR3 | Active Lever Presses | Genotype x Session | GLMM with poisson regression | 0.013799 | 0.000251539 | 0.02734716 | 0.0459 | # | **WT - KI Session 1** P=0.0387 (*)  **WT - KI Session 2** P=0.0827  **WT - KI Session 3** P=0.1418  **WT - KI Session 4** P=0.1559 |
| Riki | FR3 | Inactive Lever Presses | Genotype | GLMM with poisson regression | -0.34665 | -2.1198079 | 1.42650825 | 0.7016 | n.s. |  |
| Riki | FR3 | Inactive Lever Presses | Lever Location | GLMM with poisson regression | -1.11208 | -2.9306362 | 0.7064853 | 0.2307 | n.s. |  |
| Riki | FR3 | Inactive Lever Presses | Session | GLMM with poisson regression | 0.07374 | -0.1353367 | 0.28281574 | 0.4894 | n.s. |  |
| Riki | FR3 | Inactive Lever Presses | Genotype x Lever Location | GLMM with poisson regression | 1.28385 | -1.2608385 | 3.82853431 | 0.3227 | n.s. |  |
| Riki | FR3 | Inactive Lever Presses | Session x Lever Location | GLMM with poisson regression | -0.2139 | -0.476101 | 0.04830074 | 0.1098 | n.s. |  |
| Riki | FR3 | Inactive Lever Presses | Genotype x Session | GLMM with poisson regression | -0.23237 | -0.4926202 | 0.02788282 | 0.0801 | n.s. |  |
| Riki | FR5 | Active Lever Presses | Genotype | GLMM with poisson regression | -0.135313 | -0.33225349 | 0.061626952 | 0.178094 | n.s. |  |
| Riki | FR5 | Active Lever Presses | Lever Location | GLMM with poisson regression | -0.350503 | -0.54750982 | -0.153496494 | 0.000488 | ~~~ |  |
| Riki | FR5 | Active Lever Presses | Session | GLMM with poisson regression | 0.034046 | 0.02527435 | 0.042816855 | 2.79E-14 | $$$ |  |
| Riki | FR5 | Active Lever Presses | Genotype x Lever Location | GLMM with poisson regression | 0.202474 | -0.07614505 | 0.481092722 | 0.154355 | n.s. |  |
| Riki | FR5 | Active Lever Presses | Session x Lever Location | GLMM with poisson regression | -0.008387 | -0.01930023 | 0.002527095 | 0.132034 | n.s. |  |
| Riki | FR5 | Active Lever Presses | Genotype x Session | GLMM with poisson regression | -0.008367 | -0.01919499 | 0.002460072 | 0.129861 | n.s. |  |
| Riki | FR5 | Inactive Lever Presses | Genotype | GLMM with poisson regression | 0.4087 | -1.153796 | 1.9712031 | 0.608 | n.s. |  |
| Riki | FR5 | Inactive Lever Presses | Lever Location | GLMM with poisson regression | -0.5571 | -2.1787357 | 1.0645266 | 0.501 | n.s. |  |
| Riki | FR5 | Inactive Lever Presses | Session | GLMM with poisson regression | -0.1415 | -0.5154804 | 0.2325541 | 0.459 | n.s. |  |
| Riki | FR5 | Inactive Lever Presses | Genotype x Lever Location | GLMM with poisson regression | 0.3602 | -1.889574 | 2.6099344 | 0.754 | n.s. |  |
| Riki | FR5 | Inactive Lever Presses | Session x Lever Location | GLMM with poisson regression | -0.2025 | -0.6083153 | 0.2032266 | 0.328 | n.s. |  |
| Riki | FR5 | Inactive Lever Presses | Genotype x Session | GLMM with poisson regression | 0.3145 | -0.100299 | 0.72935 | 0.137 | n.s. |  |
| Riki | PR 2HR ALL | Active Lever Presses | Genotype | GLMM with poisson regression | -0.06682 | -0.33551349 | 0.20186545 | 0.626 | n.s. |  |
| Riki | PR 2HR ALL | Active Lever Presses | Lever Location | GLMM with poisson regression | -0.06971 | -0.33838966 | 0.19897219 | 0.611 | n.s. |  |
| Riki | PR 2HR ALL | Active Lever Presses | Session | GLMM with poisson regression | -0.08277 | -0.10321252 | -0.06232155 | 2.12E-15 | $$$ |  |
| Riki | PR 2HR ALL | Active Lever Presses | Genotype x Lever Location | GLMM with poisson regression | -0.12101 | -0.51285653 | 0.27083362 | 0.545 | n.s. |  |
| Riki | PR 2HR ALL | Active Lever Presses | Genotype x Session | GLMM with poisson regression | 0.09131 | 0.06321271 | 0.11940402 | 1.89E-10 | ### | **WT - KI Session 1** P=0.0527  **WT - KI Session 2** P=0.123  **WT - KI Session 3** P=0.8275 |
| Riki | PR 2HR ALL | Active Lever Presses | Session x Lever Location | GLMM with poisson regression | 0.09065 | 0.0617591 | 0.11953436 | 7.74E-10 | <<< | **Beside - Opposite Session 1** P=0.0225 (*)  **Beside - Opposite Session 2** P=0.4917  **Beside - Opposite Session 3** P=0.5051 |
| Riki | PR 2HR ALL | Active Lever Presses | Genotype x Session x Lever Location | GLMM with poisson regression | -0.01602 | -0.05545301 | 0.02341688 | 0.426 | n.s. |  |
| Riki | PR 2HR ALL | Inactive Lever Presses | Genotype | GLMM with poisson regression | -0.25887 | -1.930138506 | 1.4123958 | 0.7614 | n.s. |  |
| Riki | PR 2HR ALL | Inactive Lever Presses | Lever Location | GLMM with poisson regression | -1.1086 | -2.788679185 | 0.5714765 | 0.1959 | n.s. |  |
| Riki | PR 2HR ALL | Inactive Lever Presses | Session | GLMM with poisson regression | 0.27149 | 0.212419642 | 0.3305517 | 2.00E-16 | $$$ |  |
| Riki | PR 2HR ALL | Inactive Lever Presses | Genotype x Lever Location | GLMM with poisson regression | 0.37414 | -2.077690316 | 2.8259689 | 0.7649 | n.s. |  |
| Riki | PR 2HR ALL | Inactive Lever Presses | Genotype x Session | GLMM with poisson regression | 0.13589 | 0.020542354 | 0.2512316 | 0.0209 | # | **WT - KI Session 1** P=0.766  **WT - KI Session 2** P=0.6552  **WT - KI Session 3** P=0.394 |
| Riki | PR 2HR ALL | Inactive Lever Presses | Session x Lever Location | GLMM with poisson regression | -0.46342 | -0.549060562 | -0.377787 | 2.00E-16 | <<< | **Beside - Opposite Session 1** P=0.3707  **Beside - Opposite Session 2** P=0.1394  **Beside - Opposite Session 3** P=0.0091 (**) |
| Riki | PR 2HR ALL | Inactive Lever Presses | Genotype x Session x Lever Location | GLMM with poisson regression | 0.14754 | 0.004703451 | 0.2903789 | 0.0429 | } | Three-way interactions are difficult to interpret. |
| Riki | PR 2HR COLLAPSED | Active Lever Presses | Genotype | GLMM with poisson regression | -0.08836 | -0.3782669 | 0.2015563 | 0.55 | n.s. |  |
| Riki | PR 2HR COLLAPSED | Active Lever Presses | Lever Location | GLMM with poisson regression | -0.09108 | -0.3809773 | 0.1988229 | 0.538 | n.s. |  |
| Riki | PR 2HR COLLAPSED | Active Lever Presses | Genotype x Lever Location | GLMM with poisson regression | -0.09667 | -0.5194785 | 0.3261362 | 0.654 | n.s. |  |
| Riki | PR 2HR COLLAPSED | Inactive Lever Presses | Genotype | GLMM with poisson regression | -0.2621 | -1.8597577 | 1.3355566 | 0.748 | n.s. |  |
| Riki | PR 2HR COLLAPSED | Inactive Lever Presses | Lever Location | GLMM with poisson regression | -1.0196 | -2.6263135 | 0.5870439 | 0.214 | n.s. |  |
| Riki | PR 2HR COLLAPSED | Inactive Lever Presses | Genotype x Lever Location | GLMM with poisson regression | 0.3332 | -2.0115441 | 2.6780093 | 0.781 | n.s. |  |
| Riki | PR 6HR COLLAPSED | Active Lever Presses | Genotype | GLM with negative binomial regression | -0.11515 | -0.5398747 | 0.3095784 | 0.595 | n.s. |  |
| Riki | PR 6HR COLLAPSED | Active Lever Presses | Lever Location | GLM with negative binomial regression | -0.03846 | -0.4631552 | 0.3862428 | 0.859 | n.s. |  |
| Riki | PR 6HR COLLAPSED | Active Lever Presses | Genotype x Lever Location | GLM with negative binomial regression | 0.01921 | -0.5814572 | 0.6198796 | 0.95 | n.s. |  |
| Riki | PR 6HR COLLAPSED | Inactive Lever Presses | Genotype | GLM with negative binomial regression | -0.1652 | -1.451046 | 1.1205606 | 0.801 | n.s. |  |
| Riki | PR 6HR COLLAPSED | Inactive Lever Presses | Lever Location | GLM with negative binomial regression | -0.5684 | -1.854601 | 0.7177568 | 0.386 | n.s. |  |
| Riki | PR 6HR COLLAPSED | Inactive Lever Presses | Genotype x Lever Location | GLM with negative binomial regression | 0.5582 | -1.260592 | 2.3769175 | 0.548 | n.s. |  |

**Figures 9 and 10**.

| **Experimenter** | **Stage** | **Dependent Variable** | **Independent Variable** | **Stats Test** | **Coefficient** | **Lower 2.5% CI** | **Upper 97.5% CI** | **P-Value** | **Stats Summary** | **Pairwise Notes** |
| --- | --- | --- | --- | --- | --- | --- | --- | --- | --- | --- |
| Carlos | PR ALL | Blank Touch Rate | Genotype | GLMM with beta regression | -0.129108 | -0.5342833 | 0.2760666 | 0.532 | n.s. |  |
| Carlos | PR ALL | Blank Touch Rate | Session | GLMM with beta regression | 0.013661 | -0.08832535 | 0.1156482 | 0.793 | n.s. |  |
| Carlos | PR ALL | Blank Touch Rate | Genotype x Session | GLMM with beta regression | -0.002578 | -0.14615014 | 0.1409941 | 0.972 | n.s. |  |
| Carlos | PR + TIMEOUT | Blank Touch Rate | Genotype | GLMM with beta regression | -0.12146 | -0.4578294 | 0.21490894 | 0.479 | n.s. |  |
| Carlos | PR + TIMEOUT | Blank Touch Rate | Session | GLMM with beta regression | 0.09085 | -0.0457349 | 0.22742703 | 0.192 | n.s. |  |
| Carlos | PR + TIMEOUT | Blank Touch Rate | Genotype x Session | GLMM with beta regression | -0.12818 | -0.3158362 | 0.05946745 | 0.181 | n.s. |  |
| Carlos | PR - TIMEOUT | Blank Touch Rate | Genotype | GLMM with beta regression | -0.1398 | -0.6575933 | 0.3779964 | 0.597 | n.s. |  |
| Carlos | PR - TIMEOUT | Blank Touch Rate | Session | GLMM with beta regression | 0.04696 | -0.05671576 | 0.1506266 | 0.375 | n.s. |  |
| Carlos | PR - TIMEOUT | Blank Touch Rate | Genotype x Session | GLMM with beta regression | 0.05756 | -0.08887811 | 0.2040046 | 0.441 | n.s. |  |
| Carlos | FR1 | Blank Touch Rate | Genotype | GLMM with beta regression | -0.23912 | -0.49748864 | 0.0192427 | 0.0697 | n.s. |  |
| Carlos | FR1 | Blank Touch Rate | Session | GLMM with beta regression | 0.01581 | -0.08883435 | 0.12045713 | 0.7671 | n.s. |  |
| Carlos | FR1 | Blank Touch Rate | Genotype x Session | GLMM with beta regression | -0.07241 | -0.21985384 | 0.07502705 | 0.3357 | n.s. |  |
| Carlos | FR2 | Blank Touch Rate | Genotype | GLM with beta regression | -0.09451 | -0.4035349 | 0.2145164 | 0.549 | n.s. |  |
| Carlos | FR3 | Blank Touch Rate | Genotype | GLM with beta regression | -0.1662 | -0.5187544 | 0.1864166 | 0.356 | n.s. |  |
| Carlos | FR5 | Blank Touch Rate | Genotype | GLMM with beta regression | -0.29302 | -0.7946291 | 0.20859625 | 0.2522 | n.s. |  |
| Carlos | FR5 | Blank Touch Rate | Session | GLMM with beta regression | 0.10331 | 0.0141301 | 0.19248013 | 0.0232 | $ |  |
| Carlos | FR5 | Blank Touch Rate | Genotype x Session | GLMM with beta regression | -0.19736 | -0.3219344 | -0.07278398 | 0.0019 | ## | **WT - KI Saline** P=0.7225  **WT - KI ATO** P=0.0652 |
| Carlos | PR ALL | Blank Touches | Genotype | GLMM with negative binomial regression | -0.11221 | -0.6456797 | 0.4212561 | 0.68 | n.s. |  |
| Carlos | PR ALL | Blank Touches | Session | GLMM with negative binomial regression | 0.25498 | 0.1339909 | 0.375971 | 3.62E-05 | $$$ |  |
| Carlos | PR ALL | Blank Touches | Genotype x Session | GLMM with negative binomial regression | -0.01895 | -0.1780945 | 0.1401888 | 0.815 | n.s. |  |
| Carlos | PR + TIMEOUT | Blank Touches | Genotype | GLMM with negative binomial regression | -0.08506 | -0.6308324 | 0.4607148 | 0.76 | n.s. |  |
| Carlos | PR + TIMEOUT | Blank Touches | Session | GLMM with negative binomial regression | 0.15356 | -0.04059166 | 0.347703478 | 0.1211 | n.s. |  |
| Carlos | PR + TIMEOUT | Blank Touches | Genotype x Session | GLMM with negative binomial regression | -0.26124 | -0.52222119 | -0.000250967 | 0.0498 | # | **WT- KI Session 1** P=0.5731  **WT - KI Session 2** P=0.2641 |
| Carlos | PR - TIMEOUT | Blank Touches | Genotype | GLMM with negative binomial regression | -0.12562 | -0.70121906 | 0.4499711 | 0.669 | n.s. |  |
| Carlos | PR - TIMEOUT | Blank Touches | Session | GLMM with negative binomial regression | 0.02185 | -0.10259139 | 0.1462868 | 0.731 | n.s. |  |
| Carlos | PR - TIMEOUT | Blank Touches | Genotype x Session | GLMM with negative binomial regression | 0.08043 | -0.08532563 | 0.2461815 | 0.342 | n.s. |  |
| Carlos | FR1 | Blank Touches | Genotype | GLMM with negative binomial regression | -0.33634 | -0.7054878 | 0.0328116 | 0.0741 | n.s. |  |
| Carlos | FR1 | Blank Touches | Session | GLMM with negative binomial regression | -0.38773 | -0.5070997 | -0.26835974 | 1.94E-10 | $$$ |  |
| Carlos | FR1 | Blank Touches | Genotype x Session | GLMM with negative binomial regression | -0.17395 | -0.3371197 | -0.01078431 | 0.0367 | # | **WT - KI Session 1** P=0.7762  **WT - KI Session 2** P=0.5597  **WT - KI Session 3** P=0.0197 (*)  **WT - KI Session 4** P=0.0175 (*) |
| Carlos | FR2 | Blank Touches | Genotype | GLM with negative binomial regression | -0.5252 | -0.8859773 | -0.1645106 | 0.00432 | ✱✱ |  |
| Carlos | FR3 | Blank Touches | Genotype | GLM with negative binomial regression | -0.1906 | -0.5542255 | 0.173009 | 0.304 | n.s. |  |
| Carlos | FR5 | Blank Touches | Genotype | GLMM with negative binomial regression | -0.51386 | -0.93481153 | -0.09291518 | 0.0167 | ✱ |  |
| Carlos | FR5 | Blank Touches | Session | GLMM with negative binomial regression | 0.02418 | -0.09502945 | 0.14338538 | 0.691 | n.s. |  |
| Carlos | FR5 | Blank Touches | Genotype x Session | GLMM with negative binomial regression | -0.19568 | -0.36390611 | -0.0274507 | 0.0226 | # | **WT - KI Session 1** P=0.1709  **WT - KI Session 2** P=0.0023 (**) |
| Carlos | PR ALL | Breakpoint | Genotype | GLMM with negative binomial regression | -0.005597 | -0.217581556 | 0.20638829 | 0.9587 | n.s. |  |
| Carlos | PR ALL | Breakpoint | Session | GLMM with negative binomial regression | 0.054069 | -0.007158235 | 0.11529571 | 0.0835 | n.s. |  |
| Carlos | PR ALL | Breakpoint | Genotype x Session | GLMM with negative binomial regression | -0.040527 | -0.122138729 | 0.04108559 | 0.3304 | n.s. |  |
| Carlos | PR + TIMEOUT | Breakpoint | Genotype | GLMM with negative binomial regression | 0.07533 | -0.1491963 | 0.29985908 | 0.511 | n.s. |  |
| Carlos | PR + TIMEOUT | Breakpoint | Session | GLMM with negative binomial regression | -0.11873 | -0.2143783 | -0.02308259 | 0.015 | $ |  |
| Carlos | PR + TIMEOUT | Breakpoint | Genotype x Session | GLMM with negative binomial regression | -0.10246 | -0.2297341 | 0.02481313 | 0.115 | n.s. |  |
| Carlos | PR - TIMEOUT | Breakpoint | Genotype | GLMM with poisson regression | -0.05515 | -0.30780917 | 0.19750962 | 0.669 | n.s. |  |
| Carlos | PR - TIMEOUT | Breakpoint | Session | GLMM with poisson regression | 0.01988 | -0.03359835 | 0.07336281 | 0.466 | n.s. |  |
| Carlos | PR - TIMEOUT | Breakpoint | Genotype x Session | GLMM with poisson regression | 0.04769 | -0.02479939 | 0.1201842 | 0.197 | n.s. |  |
| Carlos | PR ALL | Discrimination Ratio | Genotype | GLMM with beta regression | -0.1017 | -0.51441801 | 0.3110233 | 0.629132 | n.s. |  |
| Carlos | PR ALL | Discrimination Ratio | Session | GLMM with beta regression | 0.1743 | 0.06290029 | 0.2856926 | 0.002165 | $$ |  |
| Carlos | PR ALL | Discrimination Ratio | Genotype x Session | GLMM with beta regression | 0.04709 | -0.10314479 | 0.1973201 | 0.539007 | n.s. |  |
| Carlos | PR + TIMEOUT | Discrimination Ratio | Genotype | GLMM with beta regression | -0.22689 | -0.6494138 | 0.1956248 | 0.293 | n.s. |  |
| Carlos | PR + TIMEOUT | Discrimination Ratio | Session | GLMM with beta regression | 0.35058 | 0.1897626 | 0.5113879 | 1.93E-05 | $$$ |  |
| Carlos | PR + TIMEOUT | Discrimination Ratio | Genotype x Session | GLMM with beta regression | -0.06811 | -0.288105 | 0.1518798 | 0.544 | n.s. |  |
| Carlos | PR - TIMEOUT | Discrimination Ratio | Genotype | GLMM with beta regression | -0.05737 | -0.503862 | 0.3891306 | 0.80118 | n.s. |  |
| Carlos | PR - TIMEOUT | Discrimination Ratio | Session | GLMM with beta regression | 0.0143 | -0.1178896 | 0.1464871 | 0.8321 | n.s. |  |
| Carlos | PR - TIMEOUT | Discrimination Ratio | Genotype x Session | GLMM with beta regression | 0.01016 | -0.1675644 | 0.1878926 | 0.91075 | n.s. |  |
| Carlos | FR1 | Discrimination Ratio | Genotype | GLMM with beta regression | -0.19834 | -0.693254 | 0.2965764 | 0.432183 | n.s. |  |
| Carlos | FR1 | Discrimination Ratio | Session | GLMM with beta regression | -0.49589 | -0.6172393 | -0.3745484 | 1.15E-15 | $$$ |  |
| Carlos | FR1 | Discrimination Ratio | Genotype x Session | GLMM with beta regression | -0.30079 | -0.4704487 | -0.1311363 | 0.000511 | ### | **WT - KI Session 1** P=0.4377  **WT - KI Session 2** P=0.6436  **WT - KI Session 3** P=0.1911  **WT - KI Session 4** P=0.184  **WT - KI Session 5** P=0.0542 |
| Carlos | FR2 | Discrimination Ratio | Genotype | GLM with beta regression | -0.4016 | -0.7539719 | -0.04915866 | 0.0255 | ✱ |  |
| Carlos | FR3 | Discrimination Ratio | Genotype | GLM with beta regression | -0.2104 | -0.5663632 | 0.1455805 | 0.247 | n.s. |  |
| Carlos | FR5 | Discrimination Ratio | Genotype | GLMM with beta regression | -0.47316 | -0.89516849 | -0.05114402 | 0.028 | ✱ |  |
| Carlos | FR5 | Discrimination Ratio | Session | GLMM with beta regression | 0.02736 | -0.08934864 | 0.14407345 | 0.6459 | n.s. |  |
| Carlos | FR5 | Discrimination Ratio | Genotype x Session | GLMM with beta regression | -0.18789 | -0.35783067 | -0.01794011 | 0.0302 | # | **WT - KI Session 1** P=0.2213  **WT - KI Session 2** P=0.0047 (***) |
| Carlos | PR ALL | Magazine Entry Rate | Genotype | GLMM with beta regression | 0.3329 | 0.01533296 | 0.65046744 | 0.0399 | ✱ |  |
| Carlos | PR ALL | Magazine Entry Rate | Session | GLMM with beta regression | -0.05685 | -0.16697001 | 0.05326024 | 0.3116 | n.s. |  |
| Carlos | PR ALL | Magazine Entry Rate | Genotype x Session | GLMM with beta regression | -0.15501 | -0.29305365 | -0.0169663 | 0.0277 | # | **WT - KI Session 1** P=0.0068 (**)  **WT - KI Session 2** P=0.1118   **WT - KI Session 3** P=0.0181 (*)  **WT - KI Session 4** P=0.2412  **WT - KI Session 5** P=0.4461  **WT - KI Session 6** P=0.7199 |
| Carlos | PR + TIMEOUT | Magazine Entry Rate | Genotype | GLMM with beta regression | 0.48681 | 0.11563244 | 0.85798752 | 0.0102 | ✱ |  |
| Carlos | PR + TIMEOUT | Magazine Entry Rate | Session | GLMM with beta regression | 0.05813 | -0.09634677 | 0.21260566 | 0.4608 | n.s. |  |
| Carlos | PR + TIMEOUT | Magazine Entry Rate | Genotype x Session | GLMM with beta regression | -0.14031 | -0.33071907 | 0.05009123 | 0.1486 | n.s. |  |
| Carlos | PR - TIMEOUT | Magazine Entry Rate | Genotype | GLMM with beta regression | 0.24747 | -0.1424296 | 0.63736724 | 0.2135 | n.s. |  |
| Carlos | PR - TIMEOUT | Magazine Entry Rate | Session | GLMM with beta regression | -0.02332 | -0.1357503 | 0.08911341 | 0.6844 | n.s. |  |
| Carlos | PR - TIMEOUT | Magazine Entry Rate | Genotype x Session | GLMM with beta regression | -0.19161 | -0.3368129 | -0.04640072 | 0.0097 | ## | **WT - KI Session 1** P=0.0123 (*)  **WT - KI Session 2** P=0.2695  **WT - KI Session 3** P=0.6003  **WT - KI Session 4** P=0.9257 |
| Carlos | FR1 | Magazine Entry Rate | Genotype | GLMM with beta regression | 0.19664 | -0.0464851 | 0.4397584 | 0.11292 | n.s. |  |
| Carlos | FR1 | Magazine Entry Rate | Session | GLMM with beta regression | 0.14619 | 0.05825353 | 0.2341322 | 0.00112 | $$ |  |
| Carlos | FR1 | Magazine Entry Rate | Genotype x Session | GLMM with beta regression | 0.05813 | -0.05765226 | 0.1739041 | 0.32512 | n.s. |  |
| Carlos | FR2 | Magazine Entry Rate | Genotype | GLM with beta regression | 0.35236 | 0.1128819 | 0.5918458 | 0.00393 | ✱✱ |  |
| Carlos | FR3 | Magazine Entry Rate | Genotype | GLM with beta regression | 0.1203 | -0.1793879 | 0.4199696 | 0.431 | n.s. |  |
| Carlos | FR5 | Magazine Entry Rate | Genotype | GLMM with beta regression | 0.235186 | -0.05070505 | 0.52107648 | 0.107 | n.s. |  |
| Carlos | FR5 | Magazine Entry Rate | Session | GLMM with beta regression | 0.012383 | -0.07434962 | 0.09911617 | 0.78 | n.s. |  |
| Carlos | FR5 | Magazine Entry Rate | Genotype x Session | GLMM with beta regression | -0.002715 | -0.11440807 | 0.10897882 | 0.962 | n.s. |  |
| Carlos | PR ALL | Magazine Entries | Genotype | GLMM with negative binomial regression | 0.37097 | 0.005221838 | 0.7367127 | 0.046818 | ✱ |  |
| Carlos | PR ALL | Magazine Entries | Session | GLMM with negative binomial regression | 0.23891 | 0.108529873 | 0.3692842 | 0.000329 | $$$ |  |
| Carlos | PR ALL | Magazine Entries | Genotype x Session | GLMM with negative binomial regression | -0.19529 | -0.36721952 | -0.02336217 | 0.025995 | # | **WT - KI Session 1** P=0.0017 (**)  **WT - KI Session 2** P=0.2909  **WT - KI Session 3** P=0.0137 (*)  **WT - KI Session 4** P=0.2547  **WT - KI Session 5** P=0.4244  **WT - KI Session 6** P=0.7068 |
| Carlos | PR + TIMEOUT | Magazine Entries | Genotype | GLMM with negative binomial regression | 0.5448 | 0.09725906 | 0.99233108 | 0.017 | ✱ |  |
| Carlos | PR + TIMEOUT | Magazine Entries | Session | GLMM with negative binomial regression | 0.09523 | -0.08129318 | 0.27174562 | 0.2904 | n.s. |  |
| Carlos | PR + TIMEOUT | Magazine Entries | Genotype x Session | GLMM with negative binomial regression | -0.24886 | -0.48009732 | -0.01761805 | 0.0349 | # | **WT - KI Session 1** P=0.002 (**)  **WT - KI Session 2** P=0.2448 |
| Carlos | PR - TIMEOUT | Magazine Entries | Genotype | GLMM with negative binomial regression | 0.29238 | -0.09377429 | 0.67852837 | 0.1378 | n.s. |  |
| Carlos | PR - TIMEOUT | Magazine Entries | Session | GLMM with negative binomial regression | -0.04161 | -0.1649783 | 0.08175712 | 0.5086 | n.s. |  |
| Carlos | PR - TIMEOUT | Magazine Entries | Genotype x Session | GLMM with negative binomial regression | -0.14488 | -0.30849361 | 0.01874111 | 0.0827 | n.s. |  |
| Carlos | FR1 | Magazine Entries | Genotype | GLMM with negative binomial regression | 0.05862 | -0.1230697 | 0.24031882 | 0.5271 | n.s. |  |
| Carlos | FR1 | Magazine Entries | Session | GLMM with negative binomial regression | -0.25975 | -0.3210751 | -0.1984292 | 2.00E-16 | $$$ |  |
| Carlos | FR1 | Magazine Entries | Genotype x Session | GLMM with negative binomial regression | -0.09657 | -0.1780589 | -0.01509016 | 0.0202 | # | **WT - KI Session 1** P=0.0403 (*)  **WT - KI Session 2** P=0.6775  **WT - KI Session 3** P=0.9383  **WT - KI Session 4** P=0.8902  **WT - KI Session 5** P=0.7994 |
| Carlos | FR2 | Magazine Entries | Genotype | GLM with negative binomial regression | -0.01729 | -0.1791641 | 0.1445811 | 0.834 | n.s. |  |
| Carlos | FR3 | Magazine Entries | Genotype | GLM with negative binomial regression | 0.05075 | -0.1022072 | 0.203713 | 0.515 | n.s. |  |
| Carlos | FR5 | Magazine Entries | Genotype | GLMM with negative binomial regression | 0.005312 | -0.2118006 | 0.22242506 | 0.9618 | n.s. |  |
| Carlos | FR5 | Magazine Entries | Session | GLMM with negative binomial regression | -0.071747 | -0.1333942 | -0.01009924 | 0.0225 | $ |  |
| Carlos | FR5 | Magazine Entries | Genotype x Session | GLMM with negative binomial regression | -0.029088 | -0.1131397 | 0.05496431 | 0.4976 | n.s. |  |
| Carlos | PR ALL | Reward Collection Latency | Genotype | GLMM with negative binomial regression | -0.03159 | -0.113022 | 0.04984099 | 0.447 | n.s. | **Note**: Outliers <1.5x IQR of Q1 and >1.5x IQR of Q3 removed as they were preventing convergence. |
| Carlos | PR ALL | Reward Collection Latency | Session | GLMM with negative binomial regression | -0.01308 | -0.03160734 | 0.00543653 | 0.166 | n.s. | **Note**: Outliers <1.5x IQR of Q1 and >1.5x IQR of Q3 removed as they were preventing convergence. |
| Carlos | PR ALL | Reward Collection Latency | Genotype x Session | GLMM with negative binomial regression | 0.0119 | -0.01260604 | 0.03640027 | 0.341 | n.s. | **Note**: Outliers <1.5x IQR of Q1 and >1.5x IQR of Q3 removed as they were preventing convergence. |
| Carlos | PR + TIMEOUT | Reward Collection Latency | Genotype | GLMM with log-adjusted inverse gaussian regression | -0.027177 | -0.1423063 | 0.08795305 | 0.64361 | n.s. |  |
| Carlos | PR + TIMEOUT | Reward Collection Latency | Session | GLMM with log-adjusted inverse gaussian regression | -0.008571 | -0.06285316 | 0.04571145 | 0.75697 | n.s. |  |
| Carlos | PR + TIMEOUT | Reward Collection Latency | Genotype x Session | GLMM with log-adjusted inverse gaussian regression | -0.006797 | -0.07977656 | 0.06618257 | 0.85516 | n.s. |  |
| Carlos | PR - TIMEOUT | Reward Collection Latency | Genotype | GLMM with log-adjusted inverse gaussian regression | -0.02631 | -0.101130409 | 0.048505063 | 0.491 | n.s. | **Note**: Outliers <1.5x IQR of Q1 and >1.5x IQR of Q3 removed as they were preventing convergence. |
| Carlos | PR - TIMEOUT | Reward Collection Latency | Session | GLMM with log-adjusted inverse gaussian regression | -0.01507 | -0.038303834 | 0.008172089 | 0.204 | n.s. | **Note**: Outliers <1.5x IQR of Q1 and >1.5x IQR of Q3 removed as they were preventing convergence. |
| Carlos | PR - TIMEOUT | Reward Collection Latency | Genotype x Session | GLMM with log-adjusted inverse gaussian regression | 0.02416 | -0.006984013 | 0.055304259 | 0.128 | n.s. | **Note**: Outliers <1.5x IQR of Q1 and >1.5x IQR of Q3 removed as they were preventing convergence. |
| Carlos | FR1 | Reward Collection Latency | Genotype | GLMM with log-adjusted inverse gaussian regression | -0.148714 | -0.25810167 | -0.03932551 | 0.00771 | ✱✱ |  |
| Carlos | FR1 | Reward Collection Latency | Session | GLMM with log-adjusted inverse gaussian regression | -0.150397 | -0.19304753 | -0.10774713 | 4.80E-12 | $$$ |  |
| Carlos | FR1 | Reward Collection Latency | Genotype x Session | GLMM with log-adjusted inverse gaussian regression | -0.005733 | -0.06268415 | 0.05121719 | 0.84358 | n.s. |  |
| Carlos | FR2 | Reward Collection Latency | Genotype | GLMM with log-adjusted inverse gaussian regression | -0.163103 | -0.3180798 | -0.008125915 | 0.0391 | ✱ |  |
| Carlos | FR3 | Reward Collection Latency | Genotype | GLMM with log-adjusted inverse gaussian regression | -0.064 | -0.1561451 | 0.02814938 | 0.173441 | n.s. |  |
| Carlos | FR5 | Reward Collection Latency | Genotype | GLMM with log-adjusted inverse gaussian regression | -0.09292 | -0.23389486 | 0.04806454 | 0.196 | n.s. |  |
| Carlos | FR5 | Reward Collection Latency | Session | GLMM with log-adjusted inverse gaussian regression | -0.02299 | -0.05722501 | 0.01125234 | 0.188 | n.s. |  |
| Carlos | FR5 | Reward Collection Latency | Genotype x Session | GLMM with log-adjusted inverse gaussian regression | 0.03113 | -0.01461883 | 0.07688016 | 0.182 | n.s. |  |
| Carlos | PR ALL | Schedule Length | Genotype | Cox proportional hazards model | -0.03802 | 0.57828 | 1.6027 | 0.884 | n.s. |  |
| Carlos | PR ALL | Schedule Length | Session | Cox proportional hazards model | -2.33014 | 0.06894 | 0.1373 | 2.00E-16 | $$$ |  |
| Carlos | PR ALL | Schedule Length | Genotype x Session | Cox proportional hazards model | 0.16643 | 0.72508 | 1.9239 | 0.504 | n.s. |  |
| Carlos | PR + TIMEOUT | Schedule Length | Genotype | Cox proportional hazards model | -0.1491 | 0.4366 | 1.7 | 0.667 | n.s. |  |
| Carlos | PR + TIMEOUT | Schedule Length | Session | Cox proportional hazards model | -0.05157 | 0.7162 | 1.26 | 0.72 | n.s. |  |
| Carlos | PR + TIMEOUT | Schedule Length | Genotype x Session | Cox proportional hazards model | 0.17241 | 0.767 | 1.841 | 0.44 | n.s. |  |
| Carlos | FR1 | Schedule Length | Genotype | Cox proportional hazards model | 0.149 | 0.6399 | 2.105 | 0.623821 | n.s. |  |
| Carlos | FR1 | Schedule Length | Session | Cox proportional hazards model | 0.5055 | 1.2608 | 2.18 | 0.000296 | $$$ |  |
| Carlos | FR1 | Schedule Length | Genotype x Session | Cox proportional hazards model | 0.3119 | 0.9686 | 1.926 | 0.075346 | n.s. |  |
| Carlos | FR2 | Schedule Length | Genotype | Cox proportional hazards model | 1.0417 | 1.161 | 6.917 | 0.0221 | ✱ |  |
| Carlos | FR3 | Schedule Length | Genotype | Cox proportional hazards model | 0.02555 | 0.4774 | 2.204 | 0.948 | n.s. |  |
| Carlos | FR5 | Schedule Length | Genotype | Cox proportional hazards model | 0.31343 | 0.7024 | 2.665 | 0.357 | n.s. |  |
| Carlos | FR5 | Schedule Length | Session | Cox proportional hazards model | 0.13748 | 0.9699 | 1.357 | 0.109 | n.s. |  |
| Carlos | FR5 | Schedule Length | Genotype x Session | Cox proportional hazards model | -0.11384 | 0.6352 | 1.254 | 0.512 | n.s. |  |
| Carlos | PR ALL | Target Touch Rate | Genotype | GLMM with beta regression | -0.01954 | -0.2927145 | 0.25362473 | 0.888476 | n.s. |  |
| Carlos | PR ALL | Target Touch Rate | Session | GLMM with beta regression | -0.20937 | -0.3227753 | -0.09595859 | 0.000296 | $$$ |  |
| Carlos | PR ALL | Target Touch Rate | Genotype x Session | GLMM with beta regression | -0.09803 | -0.2535504 | 0.05748333 | 0.216642 | n.s. |  |
| Carlos | PR + TIMEOUT | Target Touch Rate | Genotype | GLMM with beta regression | 0.14567 | -0.09635008 | 0.3876811 | 0.238 | n.s. |  |
| Carlos | PR + TIMEOUT | Target Touch Rate | Session | GLMM with beta regression | -0.2364 | -0.34089817 | -0.1319006 | 9.26E-06 | $$$ |  |
| Carlos | PR + TIMEOUT | Target Touch Rate | Genotype x Session | GLMM with beta regression | -0.07071 | -0.20737728 | 0.06595166 | 0.311 | n.s. |  |
| Carlos | PR - TIMEOUT | Target Touch Rate | Genotype | GLMM with beta regression | -0.08948 | -0.511257759 | 0.3323042 | 0.6776 | n.s. |  |
| Carlos | PR - TIMEOUT | Target Touch Rate | Session | GLMM with beta regression | 0.07884 | -0.003115953 | 0.1607862 | 0.0594 | n.s. |  |
| Carlos | PR - TIMEOUT | Target Touch Rate | Genotype x Session | GLMM with beta regression | 0.04049 | -0.070651775 | 0.1516286 | 0.4752 | n.s. |  |
| Carlos | FR1 | Target Touch Rate | Genotype | GLMM with beta regression | 0.05429 | -0.3095081 | 0.4180892 | 0.7699 | n.s. |  |
| Carlos | FR1 | Target Touch Rate | Session | GLMM with beta regression | 0.41791 | 0.30025145 | 0.5355706 | 3.37E-12 | $$$ |  |
| Carlos | FR1 | Target Touch Rate | Genotype x Session | GLMM with beta regression | 0.1952 | 0.03613106 | 0.3542705 | 0.0162 | # | **WT - KI Session 1** P=0.283  **WT - KI Session 2** P=0.8421  **WT - KI Session 3** P=0.6818  **WT - KI Session 4** P=0.2679  **WT - KI Session 5** P=0.246 |
| Carlos | FR2 | Target Touch Rate | Genotype | GLM with beta regression | 0.3427 | 0.06782964 | 0.6176361 | 0.0145 | ✱ |  |
| Carlos | FR3 | Target Touch Rate | Genotype | GLM with beta regression | 0.0568 | -0.3147971 | 0.4284048 | 0.764 | n.s. |  |
| Carlos | FR5 | Target Touch Rate | Genotype | GLMM with beta regression | 0.22645 | -0.17012298 | 0.6230295 | 0.2631 | n.s. |  |
| Carlos | FR5 | Target Touch Rate | Session | GLMM with beta regression | 0.09804 | -0.01239966 | 0.2084716 | 0.0819 | n.s. |  |
| Carlos | FR5 | Target Touch Rate | Genotype x Session | GLMM with beta regression | -0.0171 | -0.16081975 | 0.1266197 | 0.8156 | n.s. |  |
| Carlos | PR ALL | Target Touches | Genotype | GLMM with negative binomial regression | 0.01477 | -0.34011459 | 0.36964527 | 0.935 | n.s. |  |
| Carlos | PR ALL | Target Touches | Session | GLMM with negative binomial regression | 0.07659 | -0.02649512 | 0.17967163 | 0.145 | n.s. |  |
| Carlos | PR ALL | Target Touches | Genotype x Session | GLMM with negative binomial regression | -0.08519 | -0.22041732 | 0.05004711 | 0.217 | n.s. |  |
| Carlos | PR + TIMEOUT | Target Touches | Genotype | GLMM with negative binomial regression | 0.1662 | -0.2149593 | 0.54736596 | 0.3928 | n.s. |  |
| Carlos | PR + TIMEOUT | Target Touches | Session | GLMM with negative binomial regression | -0.20138 | -0.3601793 | -0.04258588 | 0.0129 | $ |  |
| Carlos | PR + TIMEOUT | Target Touches | Genotype x Session | GLMM with negative binomial regression | -0.15246 | -0.3661941 | 0.06126716 | 0.1621 | n.s. |  |
| Carlos | PR - TIMEOUT | Target Touches | Genotype | GLMM with negative binomial regression | -0.07771 | -0.50302663 | 0.3475991 | 0.72 | n.s. |  |
| Carlos | PR - TIMEOUT | Target Touches | Session | GLMM with negative binomial regression | 0.03468 | -0.05148642 | 0.1208485 | 0.43 | n.s. |  |
| Carlos | PR - TIMEOUT | Target Touches | Genotype x Session | GLMM with negative binomial regression | 0.08009 | -0.03678989 | 0.1969701 | 0.179 | n.s. |  |
| Carlos | FR1 | Target Touches | Genotype | GLMM with poisson regression | -0.07393 | -0.15832095 | 0.01046277 | 0.08598 | n.s. |  |
| Carlos | FR1 | Target Touches | Session | GLMM with poisson regression | 0.07426 | 0.02547377 | 0.12304624 | 0.00285 | $$ |  |
| Carlos | FR1 | Target Touches | Genotype x Session | GLMM with poisson regression | 0.08109 | 0.0136498 | 0.14852251 | 0.01844 | # | **WT - KI Session 1** P=0.283  **WT - KI Session 2** P=0.8421  **WT - KI Session 3** P=0.6818  **WT - KI Session 4** P=0.2679  **WT - KI Session 5** P=0.246 |
| Carlos | FR2 | Target Touches | Genotype | GLM with poisson regression | -6.46E-08 | -0.09799826 | 0.09799813 | 1 | n.s. |  |
| Carlos | FR3 | Target Touches | Genotype | GLM with poisson regression | -0.002782 | -0.08172476 | 0.07616147 | 0.945 | n.s. |  |
| Carlos | FR5 | Target Touches | Genotype | GLMM with poisson regression | -1.42E-02 | -0.0585441 | 0.03007455 | 0.529 | n.s. |  |
| Carlos | FR5 | Target Touches | Session | GLMM with poisson regression | -1.94E-12 | -0.03297279 | 0.03297279 | 1 | n.s. |  |
| Carlos | FR5 | Target Touches | Genotype x Session | GLMM with poisson regression | -1.41E-02 | -0.05882768 | 0.03062298 | 0.537 | n.s. |  |
